# Supplementary material for: Quorum Sensing and NF-κB Inhibition of Synthetic Coumaperine Derivatives from Piper nigrum
Source: Molecules. 2021 Apr 15;26(8):2293. doi: 10.3390/molecules26082293 (PMC8071387; doi:10.3390/molecules26082293)
Supplement: Supplementary file 1 [file molecules-26-02293-s001.zip › molecules-1159092-supplementary.pdf]

Supplementary Information

Quorum Sensing and NF-κB Inhibition of Synthetic Coumaperine Derivatives from Piper nigrum

Yael Kadosh1#, Subramani Muthuraman4#, Karin Yaniv1, Yifat Baruch1, Jacob Gopas2,3, Ariel Kushmaro1* and Rajendran Saravana Kumar4*

1 Avram and Stella Goldstein-Goren Department of Biotechnology Engineering, Ben Gurion University of the Negev, Be'er Sheva 8410501, Israel.
2 Department of Microbiology, Immunology and Genetics Faculty of Health Sciences, Ben Gurion University of the Negev, Be'er Sheva 8410501, Israel.
3 Department of Oncology, Soroka University Medical Center, Ben Gurion University of the Negev, Be'er Sheva 8410501, Israel.
4 Chemistry Division, (School of Advanced Sciences) VIT University Chennai Campus, Vandalur-Kelambakkam Road, Chennai-600127, India.
5 The Ilse Katz Center for Meso and Nanoscale Science and Technology, Ben-Gurion University of the Negev, Be'er Sheva 8410501, Israel.
# Equal contribution

Table of Contents

1) General methods -- 4
2) Synthesis of **CP-215** -- 5
3) Synthesis of **CP-237** -- 6
4) Synthesis of **CP-270** -- 7
5) Synthesis of **CP-273** -- 8
6) Synthesis of **CP-282** -- 9
7) Synthesis of **CP-286** -- 10
8) Synthesis of **CP-289** -- 12
9) Synthesis of **CP-291** -- 13
10) Synthesis of **CP-295** -- 14
11) Synthesis of **CP-296** -- 16
12) Synthesis of **CP-Coumaperine** -- 18
13) Synthesis of **CP-9** --- 19
14) Synthesis of **CP-10** -- 20
15) Synthesis of **CP-27** -- 21
16) Synthesis of **CP-32** -- 22
17) Synthesis of **CP-38** -- 23

18) Synthesis of **CP-50** ---------- 23
19) Synthesis of **CP-123** ---------- 24
20) Synthesis of **CP-147** ---------- 25
21) Synthesis of **CP-154**---------- 26
22) Synthesis of **CP-158**---------- 27
23) Synthesis of **CP-184** ---------- 27
24) Synthesis of **CP-193----------------** ---------- 28
25) Synthesis of **CP-194** ---------- 29
26) Synthesis of **CP-209** ---------- 30
27) Synthesis of **CP-262F1& CP-262F2** ---------- 31
28) Synthesis of **CP-281-F1** ---------- 33
29) Isolation of **piperine (PIP)** ---------- 34
30) Synthesis of **CP-102** ---------- 35
31) Synthesis of **CP-155** ---------- 35
32) Synthesis of **CP-239** ---------- 36
33) References ---------- 38
34) ^{1}H and ^{13}C NMR spectra of **CP-215----------**---------- 39
35) ^{1}H and ^{13}C NMR spectra of **CP-237** ---------- 40
36) ^{1}H and ^{13}C NMR spectra of **CP-270** ---------- 41
37) ^{1}H and ^{13}C NMR spectra of **CP-273** ---------- 42
38) ^{1}H and ^{13}C NMR spectra of **CP-282** ---------- 43
39) ^{1}H and ^{13}C NMR spectra of **CP-286** ---------- 44
40) ^{1}H and ^{13}C NMR spectra of **CP-289** ---------- 45
41) ^{1}H and ^{13}C NMR spectra of **CP-291** ---------- 46
42) ^{1}H and ^{13}C NMR spectra of **CP-295** ---------- 47
43) ^{1}H and ^{13}C NMR spectra of **CP-296** ---------- 48
44) ^{1}H and ^{13}C NMR spectra of **N-Crotonyl piperidine** ---------- 49
45) ^{1}H and ^{13}C NMR spectra of **CP-Coumaperine** ---------- 50
46) ^{1}H and ^{13}C NMR spectra of **CP-9** ---------- 51
47) ^{1}H and ^{13}C NMR spectra of **CP-10** ---------- 52
48) ^{1}H and ^{13}C NMR spectra of **CP-27** ---------- 53

49) ^{1}H and ^{13}C NMR spectra of **CP-32** -- 54
50) ^{1}H and ^{13}C NMR spectra of **CP-38** -- 55
51) ^{1}H and ^{13}C NMR spectra of **CP-50** -- 56
52) ^{1}H and ^{13}C NMR spectra of **CP-123** -- 57
53) ^{1}H and ^{13}C NMR spectra of **CP-147** -- 58
54) ^{1}H and ^{13}C NMR spectra of **CP-154** -- 59
55) ^{1}H and ^{13}C NMR spectra of **CP-158** -- 60
56) ^{1}H and ^{13}C NMR spectra of **CP-184** -- 61
57) ^{1}H and ^{13}C NMR spectra of **CP-193** -- 62
58) ^{1}H and ^{13}C NMR spectra of **CP-194** -- 63
59) ^{1}H and ^{13}C NMR spectra of **CP-209** -- 64
60) ^{1}H and ^{13}C NMR spectra of **CP-262F1** -- 65
61) ^{1}H and ^{13}C NMR spectra of **CP-262F2** -- 66
62) ^{1}H and ^{13}C NMR spectra of **CP-281-F1** -- 67
63) ^{1}H and ^{13}C NMR spectra of **CP-102** -- 68
64) ^{1}H and ^{13}C NMR spectra of **CP-155** -- 69
65) ^{1}H and ^{13}C NMR spectra of **CP-239** -- 70
66) Table 1 degree of QSI- low and inactive compounds------------------------------------ 71
66) Table 2 cell viability assay--- 74

1. General methods

All the commercially obtained reagents/solvents for the synthesis of coumaperine and its derivatives were used as received; chemicals were purchased from Spectrochem®, SRL®, Alfa Aesar®, RANKEM®, Fisher Scientific®, and used as received without further purification. Unless stated otherwise, the reactions were conducted in oven-dried glassware and under normal atmospheric conditions. ^{1}H NMR and ^{13}C NMR spectra were recorded on Bruker 500 MHz spectrometer operating with ^{13}C resonance frequency of 125 MHz and proton resonance frequency of 500 MHz or Bruker 400 MHz spectrometer operating with the ^{13}C resonance frequency of 100 MHz and proton resonance frequency of 400 MHz. DMSO-d6 or $CDCl_3$ with TMS as an internal standard was used as an NMR solvent. Data from the ^{1}H NMR spectroscopy are reported as chemical shift (δ ppm) with the corresponding integration values. Coupling constants (*J*) are reported in hertz (Hz). Standard abbreviations indicating multiplicity were used as follows: s (singlet), br (broad), d (doublet), t (triplet), q (quartet), and m (multiplet). Data from ^{13}C NMR spectra are reported in terms of chemical shift (δ ppm). IR spectra were recorded in Thermo Scientific Nicolet Nexus 470 FT-IR spectrometer and band positions are reported in reciprocal centimeters. Samples were made as pellets with KBr and recorded. High-resolution mass spectra were recorded with Agilent 6520 (Q-TOF) mass spectrometer by using ESI technique in positive ion mode. Melting points were recorded with REMI DDMS 2545. The instrument is calibrated with benzoic acid before the measurement.

Abbreviation used in this supporting information

DCE – Dichloroethane

DCM – Dichloromethane

DMSO – Dimethyl sulfoxide

DMF – Dimethylformamide

TEA – Triethylamine

EtOAc – Ethyl acetate

RT – Room temperature

Synthesis of monoconjugated coumaperine derivatives

Synthesis of (E)-3-(benzo[d][1,3]dioxol-5-yl)-1-(piperidin-1-yl)prop-2-en-1-one (CP-215).

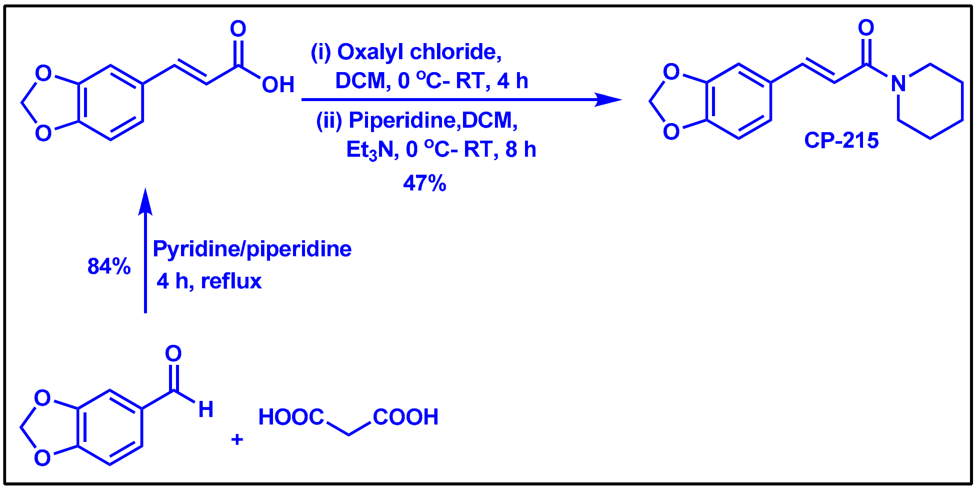

To a solution of piperonal (1.0 g, 6.66 mmol) in pyridine (10 ml) at room temperature, malonic acid (1.38 g, 13.3 mmol) was added and stirred for 10 minutes, to this 0.5 mL of piperidine was added and then the reaction mixture was refluxed for 4 h. The progress of the reaction was monitored by TLC (EtOAc). After completion, the reaction mixture was quenched with 1M HCl (20 mL). The product precipitated as white crystalline solid. It was washed with dil. HCl (25 mL), diethyl ether (10 mL) and dried under vacuum to get (E)-3,4-(methylenedioxy)cinnamic acid in 1.07 g (84% yield).

To a cold (0 ºC) solution of (E)-3,4-(methylenedioxy)cinnamic acid (1g, 5.2 mmol) in DCM under N_2 atmosphere, oxalyl chloride (1.3g, 10.3 mmol) was added slowly and the reaction mixture was slowly warmed to room temperature and stirred for 4 h. Excess oxalyl chloride was distilled off. Without further purification, the crude product was taken to the next step.

To a stirred solution of piperidine (0.44g, 5.1 mmol) and Et_3N (0.57 g, 5.6 mmol) in DCM (20 mL) at 0 °C, the above-synthesized acid chloride in DCM was added dropwise. After the addition, the reaction mass was slowly warmed to room temperature and stirred for 8 h. Progress of the reaction was monitored by TLC (hexane-EtOAc (60:40)). After completion, the DCM layer was washed with sat. $NaHCO_3$ solution (100 mL), water (2 x 75 mL), dried over anhyd. Na_2SO_4 and concentrated under reduced pressure. The obtained crude product was column purified to get the pure product as off-white solid in 0.63 g (yield 47%).

Physical Characteristics:

Colour and appearance: Off-white solid

M.pt: 88 $^{\circ}$C - 90 $^{\circ}$C

Spectral data:

^{1}H NMR (400 MHz, DMSO) δ: 1.62-1.68 (6H, m), 3.58-3.66 (4H, m), 5.99 (2H, s,), 6.74 (1H, d, *J* =16 Hz), 6.80 (1H, , *J* =8 Hz), 7.00 (1H, dd, *J* =12 Hz, *J* =4 Hz), 7.04 (1H, d, *J*), 7.57 (1H, d, *J* =12 Hz).

^{13}CNMR (100 MHz, DMSO) δ: 24.6, 43.3, 101.4, 106.3, 108.5, 115.6, 123.6, 129.9, 141.9, 147.9, 148.1, 148.6, 148.8.

IR (KBr) cm^{-1}: 1646, 1607, 1502, 1442, 1250, 1016, 854.

GC-MS (EI$^+$) ***m/z***: 259.21 (M$^+$).

Synthesis of (E)-3-(3,4-dihydroxyphenyl)-1-(piperidin-1-yl)prop-2-en-1-one (CP-237).

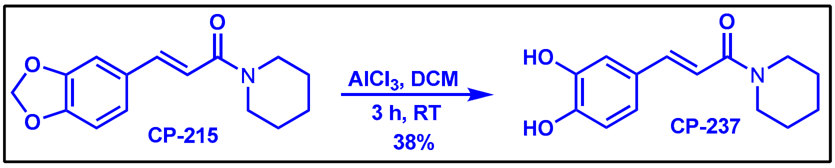

To a suspension of $AlCl_3$ (1.28g, 9.6 mmol) in DCM (10.0mL) at room temperature, a solution of **CP-215** (0.5g, 1.9 mmol) in DCM (10.0 mL) was added dropwise under N_2 atm. Stirring continued for 3 h at room temperature. After completion, the reaction mixture was cooled to 0° C, quenched with 1% HCl solution (50 mL) and poured into sat. brine solution (50 mL), extracted with EtOAc (2 x 100 mL), the combined organic layer was washed with sat. brine solution (50 mL), dried over Na_2SO_4 and concentrated under reduced pressure to get the crude product. The pure product was obtained after column purification as off-white solid in 0.18 g (yield 38%).

Physical Characteristics:

Colour and appearance: Off-white solid

M.pt: 215 $^{\circ}$C - 217 $^{\circ}$C.

Spectral data:

^{1}H NMR (400 MHz, DMSO) δ: 1.48-1.60 (6H, m), 3.58 (4H, br), 6.74 (1H, d, *J* = 10 Hz), 6.91 (1H, d, *J* = 12 Hz), 6.97 (1H, d, *J* = 10 Hz), 7.20 (1H, dd, *J* =15 Hz, *J* =10 Hz), 7.36 (2H, d, *J* = 10 Hz), 9.95 (1H, s).

^{13}CNMR (100 MHz, DMSO) δ: 24.6, 27.0, 43.0, 45.5, 46.5, 115.0, 115.1, 116.0, 120.9, 127.2, 142.3, 145.8, 147.6, 165.1.

IR (KBr) cm^{-1}: 3451, 3080, 1636, 1598, 1558, 1435, 1266, 1022, 865.

Elemental analysis calcd for $C_{14}H_{17}NO_3$: C = 68.00; H = 6.93; N = 5.63. Found: C = 67.59; H = 6.87; N = 5.49.

Synthesis of (E)-3-(benzo)-1-(piperidin-1-yl)prop-2-en-1-one (CP-270).

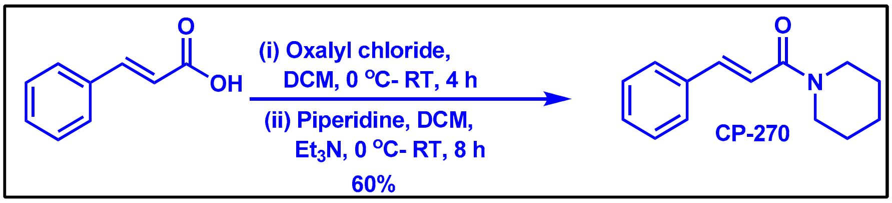

To a cold (0 ºC) solution of (E)-cinnamic acid (1g, 6.7 mmol) in DCM under N_2 atmosphere, oxalyl chloride (1.28g, 10.1 mmol) was added slowly and the reaction mixture was slowly warmed to room temperature and stirred for 4 h. Excess oxalyl chloride was distilled off. Without further purification, the crude product was taken to the next step.

To a stirred solution of piperidine (0.56g, 6.6 mmol) and Et_3N (0.73 g, 7.2 mmol) in DCM (20 mL) at 0 ˚C, the above-synthesized acid chloride in DCM was added dropwise. After the addition, the reaction mass was slowly warmed to room temperature and stirred for 8 h. Progress of the reaction was monitored by TLC (hexane-EtOAc (60:40)). After completion, the DCM layer was washed with sat. $NaHCO_3$ solution (100 mL), water (2 x 75 mL), dried over anhyd. Na_2SO_4 and concentrated under reduced pressure. The obtained crude product was column purified using hexane-EtOAc (90:10) to get the pure product as off-white solid in 0.87 g (yield 60%).

Physical Characteristics:

Colour and appearance: Off-white solid

M.pt: 117 ˚C - 118 ˚C (Lit. m.pt =118.9 °C -119.9 °C)[1]

Spectral data:

^{1}H NMR (400 MHz, DMSO) δ: 1.57-1.67 (6H, m), 3.61 (4H, br), 6.90 (1H, d, J =16 Hz), 7.30-7.38 (3H, m), 7.51 (2H, d, J =8 Hz), 7.63 (1H, d, J =16 Hz).

^{13}CNMR (100 MHz, DMSO) δ: 19.9, 20.9, 21.9, 38.6, 42.2, 113.0, 122.9, 124.0, 124.6, 130.6, 137.3, 160.6.

IR (KBr) cm^{-1}: 2934, 1644, 1441, 1248, 1224, 1018, 852, 763.

GC-MS (EI$^+$) ***m/z***: 215.05 (M$^+$).

Synthesis of (E)-3-(benzoyl 4-fluoro)-1-(piperidin-1-yl)prop-2-en-1-one (CP-273).

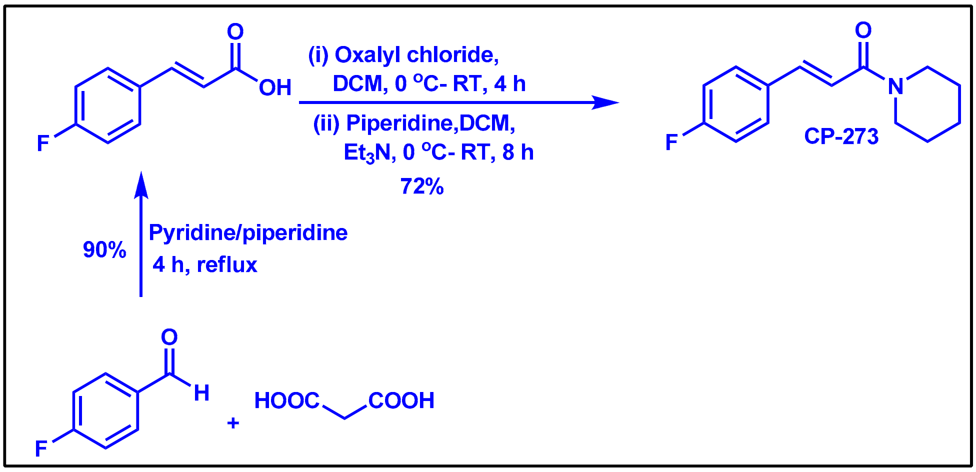

To a solution of 4-fluorobenzaldhyde (1.0 g, 8.06 mmol) in pyridine (10 ml) at room temperature, malonic acid (1.67 g, 16.1 mmol) was added and stirred for 10 minutes, to this 0.5ml of piperidine was added and then the reaction mixture was refluxed for 4 h. The progress of the reaction was monitored by TLC (EtOAc). After completion, the reaction mixture was quenched with 1M HCl (20 mL). The product precipitated as white crystalline solid. It was washed with dil. HCl (10 mL), diethyl ether (10 mL) and dried under vacuum to get (E)-4-fluorocinnamic acid in 1.20 g (90% yield).

To a cold (0 ºC) solution of 4-fluorocinnamic acid (1g, 6.0 mmol) in DCM under N_2 atmosphere, oxalyl chloride (1.28g, 10.1 mmol) was added slowly and the reaction mixture was slowly warmed to room temperature and stirred for 4 h. Excess oxalyl chloride was distilled off. Without further purification, the crude product was taken to the next step.

To a stirred solution of piperidine (0.46g, 5.4 mmol**)** and Et_3N (0.73 g, 7.2 mmol) in DCM (20 mL) at 0 $^{\circ}$C, the above-synthesized acid chloride in DCM was added dropwise. After the addition, the reaction mass was slowly warmed to room temperature and stirred for 8 h. Progress of the reaction was monitored by TLC (hexane-EtOAc (60:40)). After completion, the DCM layer was washed with sat. $NaHCO_3$ solution (100 mL), water (2 x 75 mL), dried over anhyd. Na_2SO_4 and concentrated under reduced pressure. The obtained crude product was column purified using hexane-EtOAc (90:10) to get the pure product as off-white solid in 1.01 g (yield 72%).

Physical Characteristics:

Colour and appearance: Off-white solid

M.pt: 136 °C - 137 °C

Spectral data:

^{1}**H NMR** (400 MHz, DMSO) δ: 1.58-1.71 (6H, m), 3.62 (4H, br), 6.83 (1H, d, *J* =16 Hz), 7.03-7.08 (2H, m), 7.48-7.52 (2H, m), 7.61 (1H, d, *J* =16 Hz).

^{13}**CNMR** (100 MHz, DMSO) δ: 24.6, 26.6, 26.8, 43.3, 46.7, 115.7, 115.9, 117.4, 117.5, 129.4, 131.7, 140.9, 162.1, 164.6, 165.1.

IR (KBr) cm^{-1}: 3042, 2936, 2851, 1648, 1584, 1345, 1248, 1013, 880.

GC-MS (EI$^+$) ***m/z***: 233.47 (M^+).

Synthesis of (E)-3-(benzoyl 4-methoxy)-1-(piperidin-1-yl)prop-2-en-1-one (CP-282).

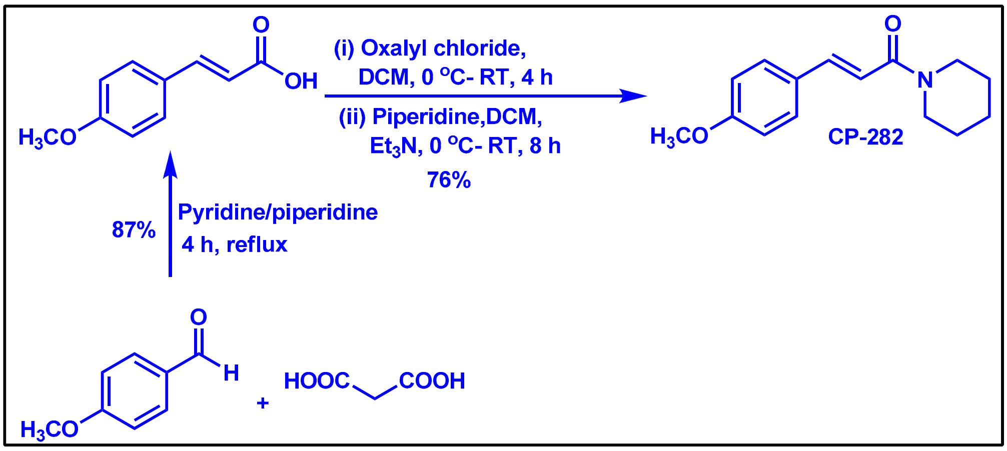

To a solution of 4-methoxybenzaldhyde (1.0 g, 7.34 mmol) in pyridine (10 mL) at room temperature, malonic acid (1.67 g, 16.1 mmol) was added and stirred for 10 minutes, to this 0.5ml of piperidine was added and then the reaction mixture was refluxed for 4 h. The progress of the reaction was monitored by TLC (EtOAc). After completion, the reaction mixture was quenched with 1M HCl (20 mL). The product precipitated as white crystalline solid. It was washed with dil. HCl (25 mL), diethyl ether (10 mL) and dried under vacuum to get (E)-4-methoxy cinnamic acid in 1.13 g (87% yield).

To a cold (0 °C) solution of 4-methoxy cinnamic acid (1g, 5.6 mmol) in DCM under N_2 atmosphere, oxalyl chloride (1.06g, 8.4 mmol) was added slowly and the reaction mixture was slowly warmed to room temperature and stirred for 4 h. Excess oxalyl chloride was distilled off. Without further purification, the crude product was taken to the next step.

To a stirred solution of piperidine (0.43g, 5.0 mmol) and Et_3N (0.62 g, 6.1 mmol) in DCM (20 mL) at 0 °C, the above-synthesized acid chloride in DCM was added dropwise. After the addition, the reaction mass was slowly warmed to room temperature and stirred for 8 h. Progress of the reaction was monitored by TLC (hexane-EtOAc (60:40)). After completion, the DCM layer was washed with sat. $NaHCO_3$ solution (100 mL), water (2 x 75 mL), dried over anhyd. Na_2SO_4 and concentrated under reduced pressure. The obtained crude product was column purified to get the pure product as off-white solid in 1.04 g (yield 76%).

<u>Physical Characteristics:</u>

Colour and appearance: Off-white solid

M.pt: 72 °C - 73 °C (Lit. m.pt = 71 °C -72 °C)[2]

<u>Spectral data:</u>

^{1}H NMR (400 MHz, DMSO) δ: 1.60-1.68 (6H, m), 3.59-3.64 (4H, br, d), 3.82 (3H, s) 6.78 (1H, d, J=12 Hz), 6.88 (1H, d, J=12 Hz), 7.47 (1H, d, J=12 Hz), 7.61 (1H, d, J=16 Hz).

^{13}CNMR (100 MHz, DMSO) δ: 24.6, 25.6, 26.7, 43.3, 55.3, 114.1, 115.2, 128.2, 129.1, 141.8, 160.7, 165.6.

IR (KBr) cm^{-1}: 3034, 2934, 2863, 1642, 1586, 1119, 1016, 877, 630.

GC-MS (EI$^+$) ***m/z***: 245.05 (M^+).

Synthesis of (E)-3-(benzoyl 4-nitro)-1-(piperidin-1-yl)prop-2-en-1-one (CP-286).

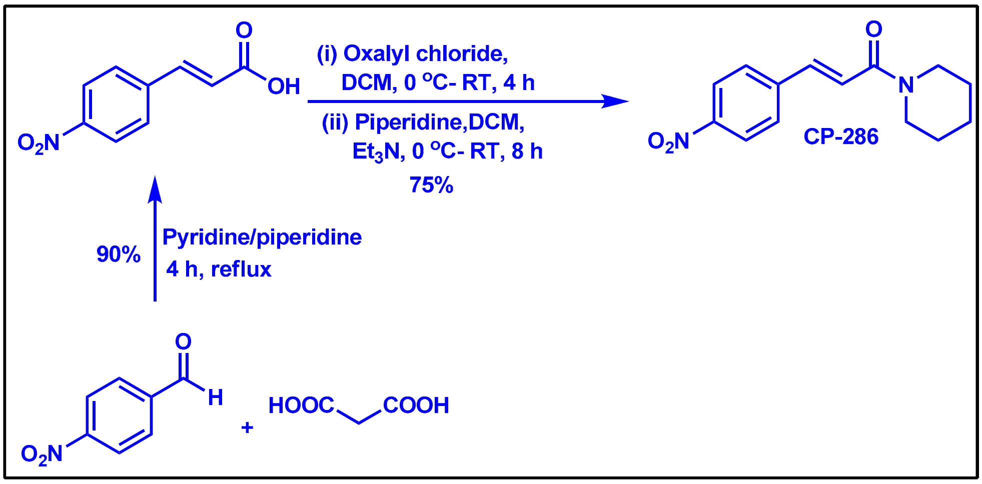

To a solution of 4-nitrobenzaldehyde (1.0 g, 7.34 mmol) in pyridine (10 mL) at room temperature, malonic acid (1.67 g, 16.1 mmol) was added and stirred for 10 minutes, to this 0.5ml of piperidine was added and then the reaction mixture was refluxed for 4 h. The progress of the reaction was monitored by TLC (EtOAc). After completion, the reaction mixture was quenched with 1M HCl

(20 mL). The product precipitated as white crystalline solid. It was washed with dil. HCl (25 mL), diethyl ether (10 mL) and dried under vacuum to get (E)-4-nitrocinnamic acid in 1.15 g (90% yield).

To a cold (0 ºC) solution of 4-nitrocinnamic acid (1g, 5.1 mmol) in DCM under N_2 atmosphere, oxalyl chloride (0.98g, 7.7 mmol) was added slowly and the reaction mixture was slowly warmed to room temperature and stirred for 4 h. Excess oxalyl chloride was distilled off. Without further purification, the crude product was taken to the next step.

To a stirred solution of piperidine (0.40g, 4.7 mmol**)** and Et_3N (0.72 g, 7.0 mmol) in DCM (20 mL) at 0 ˚C, the above-synthesized acid chloride in DCM was added dropwise. After the addition, the reaction mass was slowly warmed to room temperature and stirred for 8 h. Progress of the reaction was monitored by TLC (hexane-EtOAc (60:40)). After completion, the DCM layer was washed with sat. $NaHCO_3$ solution (100 mL), water (2 x 75 mL), dried over anhyd. Na_2SO_4 and concentrated under reduced pressure. The obtained crude product was column purified using hexane-EtOAc (90:10) to get the pure product as a white solid in 1.01 g (yield 75%).

<u>Physical Characteristics:</u>

Colour and appearance: White solid

M.pt: 171 ˚C - 172 ˚C (Lit. m.pt = 71 °C -72 °C)[3]

<u>Spectral data:</u>

^{1}H NMR (400 MHz, DMSO) δ: 1.64-1.71 (6H, m), 3.60-3.68 (4H, br, d), 7.05 (1H, d, *J* =16 Hz), 7.03-7.68 (3H, m), 8.23 (2H, d, *J* =8 Hz).

^{13}CNMR (100 MHz, DMSO) δ: 24.5, 25.5, 26.8, 43.5, 47.1, 122.1, 124.1, 128.2, 139.3, 141.7, 147.9, 164.3.

IR (KBr) cm^{-1}: 2951, 2854, 1648, 1511, 1468, 1341, 1216, 1137, 1018, 978, 866, 753.

GC-MS (EI$^+$) ***m/z***: 260.10 (M^+).

Synthesis of (E)-3-(benzoyl 3,4-dimethoxy)-1-(piperidin-1-yl)prop-2-en-1-one (CP-289).

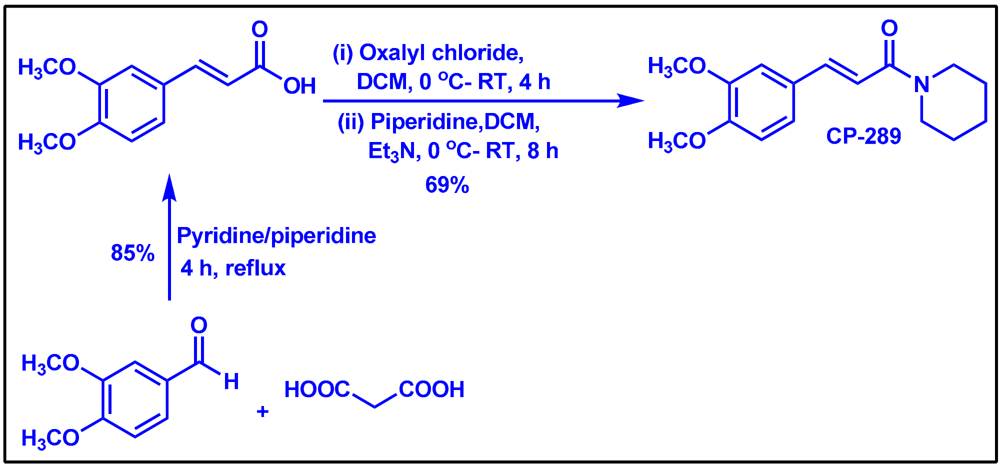

To a solution of 3,4-dimethoxybenzaldhyde (1.0 g, 7.34 mmol) in pyridine (10 ml) at room temperature, malonic acid (1.67 g, 16.1 mmol) was added and stirred for 10 minutes, to this 0.5ml of piperidine was added and then the reaction mixture was refluxed for 4 h. The progress of the reaction was monitored by TLC (EtOAc). After completion, the reaction mixture was quenched with 1M HCl (20 mL). The product precipitated as white crystalline solid. It was washed with dil. HCl (25 mL), diethyl ether (10 mL) and dried under vacuum to get (E)-3,4-dimethoxycinnamic acid in 1.06 g (85% yield).

To a cold (0 ºC) solution of 3,4-methoxy cinnamic acid (1g, 5.6 mmol) in DCM under N_2 atmosphere, oxalyl chloride (1.06 g, 8.4 mmol) was added slowly and the reaction mixture was slowly warmed to room temperature and stirred for 4 h. Excess oxalyl chloride was distilled off. Without further purification, the crude product was taken to the next step.

To a stirred solution of piperidine (0.38g, 4.4 mmol) and Et_3N (0.67 g, 6.6 mmol) in DCM (20 mL) at 0 °C, the above-synthesized acid chloride (1g, 4.4 mmol) in DCM was added dropwise. After the addition, the reaction mass was slowly warmed to room temperature and stirred for 8 h. Progress of the reaction was monitored by TLC (hexane-EtOAc (60:40)). After completion, the DCM layer was washed with sat. $NaHCO_3$ solution (100 mL), water (2 x 75 mL), dried over anhyd. Na_2SO_4 and concentrated under reduced pressure. The obtained crude product was column purified to get the pure product as yellow viscous oil in 0.91 g (yield 69%).

Physical Characteristics:

Colour and appearance: Viscous oil

Spectral data:

^{1}**H NMR** (400 MHz, DMSO) δ: 1.61-1.69 (6H, m), 3.60-3.66 (4H, br, d), 3.91 (6H, d, *J* =4 Hz) 6.76 (1H, d, *J* =16 Hz), 6.86 (1H, d, *J* =8 Hz), 7.03 (1H, s), 7.09-7.11 (1H, m), 7.59 (1H, d, *J* =16 Hz).

^{13}**CNMR** (100 MHz, DMSO) δ: 24.6, 26.8, 55.9, 109.8, 111.0, 115.4, 121.6, 128.5, 142.1, 149.1, 150.3, 165.8.

GC-MS (EI^{+}) ***m/z***: 275.15 (M^{+}).

Synthesis of (E)-3-(benzoyl 3-methoxy 4-acetoxy)-1-(piperidin-1-yl)prop-2-en-1-one (CP-291).

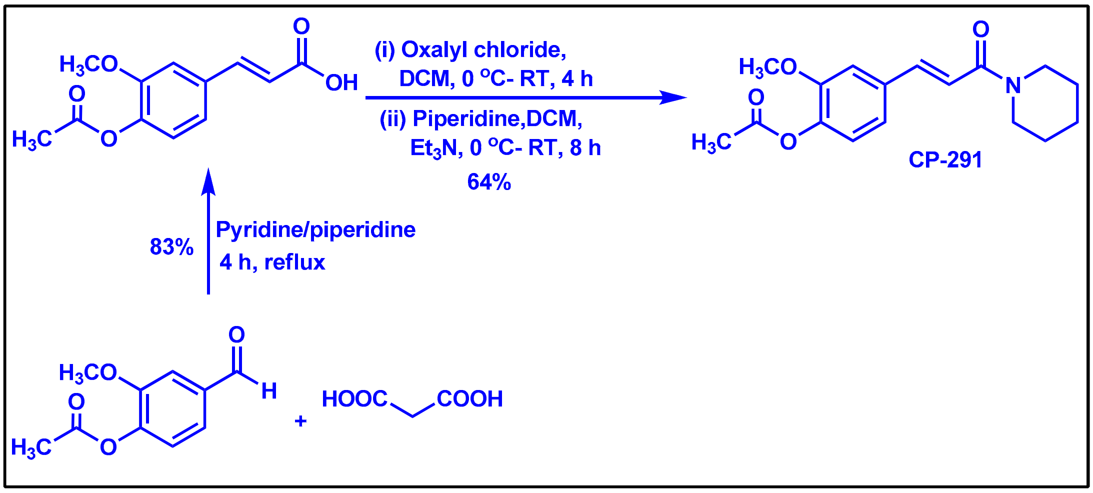

To a solution of 3-methoxy4-acetoxybenzaldhyde (1.0 g, 7.34 mmol) in pyridine (10 mL) at room temperature, malonic acid (1.67 g, 16.1 mmol) was added and stirred for 10 minutes, to this 0.5ml of piperidine was added and then the reaction mixture was refluxed for 4 h. The progress of the reaction was monitored by TLC (EtOAc). After completion, the reaction mixture was quenched with 1M HCl (20 mL). The product precipitated as white crystalline solid. It was washed with dil. HCl (25 mL), diethyl ether (10 mL) and dried under vacuum to get (E)- 3-methoxy4-acetoxycinnamic acid in 1.00 g (83% yield).

To a cold (0 ºC) solution of 3-methoxy 4-acetoxycinnamic acid (1g, 4.2 mmol) in DCM under N_2 atmosphere, oxalyl chloride (0.80g, 6.3 mmol) was added slowly and the reaction mixture was slowly warmed to room temperature and stirred for 4 h. Excess oxalyl chloride was distilled off. Without further purification, the crude product was taken to the next step.

To a stirred solution of piperidine (0.33g, 3.9 mmol**)** and Et_3N (0.60 g, 5.8 mmol) in DCM (20 mL) at 0 $^{\circ}$C, the above-synthesized acid chloride in DCM was added dropwise. After the addition, the reaction mass was slowly warmed to room temperature and stirred for 8 h. Progress of the

reaction was monitored by TLC (hexane-EtOAc (60:40)). After completion, the DCM layer was washed with sat. $NaHCO_3$ solution (100 mL), water (2 x 75 mL), dried over anhyd. Na_2SO_4 and concentrated under reduced pressure. The obtained crude product was column purified using hexane-EtOAc (90:10) to get the pure product as off-white solid in 0.82 g (yield 64%).

Physical Characteristics:

Colour and appearance: Off-white solid

M.pt: 123 $^{\circ}$C - 124 $^{\circ}$C

Spectral data:

^{1}H NMR (400 MHz, DMSO) δ: 1.62-1.69 (6H, m), 2.32 (3H, s), 3.58-3.66 (4H, br, d), 3.87 (3H, s) 6.83 (1H, d, J =16 Hz), 7.10 (2H, d, J =8 Hz), 7.07 (1H, s), 7.12-7.14 (1H, m), 7.59 (1H, d, J =16 Hz).

^{13}CNMR (100 MHz, DMSO) δ: 20.6, 24.6, 25.6, 26.7, 43.3, 47.0, 55.9, 111.5, 118.0, 120.2, 123.0, 128.6, 134.5, 141.5, 151.2, 165.1, 168.9.

IR (KBr) cm^{-1}: 3066, 2936, 2854, 1759, 1649, 1517, 1447, 1303, 1262, 1164, 1030, 830, 737.1.

GC-MS (EI^{+}) ***m/z***: 303.15 (M^{+}).

Synthesis of (E)-cyclohexyl-1-(piperidin-1-yl)prop-2-en-1-one (CP-295).

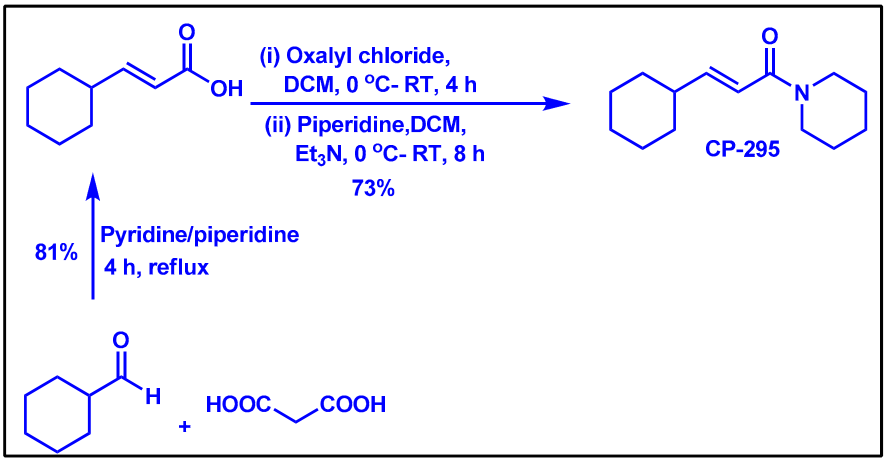

To a solution of cyclohexancarboxaldehyde (1.0 g, 8.91 mmol) in pyridine (10 mL) at room temperature, malonic acid (1.85 g, 17.8 mmol) was added and stirred for 10 minutes, to this 0.5ml of piperidine was added and then the reaction mixture was refluxed for 4 h. The progress of the reaction was monitored by TLC (EtOAc). After completion, the reaction mixture was quenched with 1M HCl (20 mL). The product precipitated as white crystalline solid. It was washed with dil.

HCl (25 mL), diethyl ether (10 mL) and dried under vacuum to get (E)-cyclohexylacrylic acid in 1.11 g (81% yield).

To a cold (0 ºC) solution of cyclohexylacrylic acid (1g, 6.4 mmol) in DCM under N_2 atmosphere, oxalyl chloride (1.23g, 9.7 mmol) was added slowly and the reaction mixture was slowly warmed to room temperature and stirred for 4 h. Excess oxalyl chloride was distilled off. Without further purification, the crude product was taken to the next step.

To a stirred solution of piperidine (0.49g, 5.7 mmol**)** and Et_3N (0.88 g, 8.6 mmol) in DCM (20 mL) at 0 °C, the above-synthesized acid chloride in DCM was added dropwise. After the addition, the reaction mass was slowly warmed to room temperature and stirred for 8 h. Progress of the reaction was monitored by TLC (hexane-EtOAc (60:40)). After completion, the DCM layer was washed with sat. $NaHCO_3$ solution (100 mL), water (2 x 75 mL), dried over anhyd. Na_2SO_4 and concentrated under reduced pressure. The obtained crude product was column purified to get the pure product as off-white solid in 1.04 g (yield 73%).

Physical Characteristics:

Colour and appearance: Off-white solid

M.pt: 66 °C - 67 °C

Spectral data:

^{1}H NMR (400 MHz, DMSO) δ: 1.16-1.31 (6H, m), 1.54-1.76 (10H, m), 2.09-2.16 (1H, m), 3.48-3.60 (4H, br) 6.19 (1H, d, *J* =16 Hz), 6.79 (1H, dd, *J* =16 Hz, *J* =8 Hz).

^{13}CNMR (100 MHz, DMSO) δ: 24.6, 25.5, 25.8, 26.0, 26.6, 32.0, 40.7, 43.1, 46.8, 117.4, 151.0, 165.9.

IR (KBr) cm^{-1}: 1650, 1613, 1502, 1442, 1349, 1272, 1123, 1020, 983, 852, 707.

GC-MS (EI^+) ***m/z***: 221.10 (M^+).

Synthesis of (E)-2-(nonenyl)-1-(piperidin-1-yl)prop-2-en-1-one (CP-296).

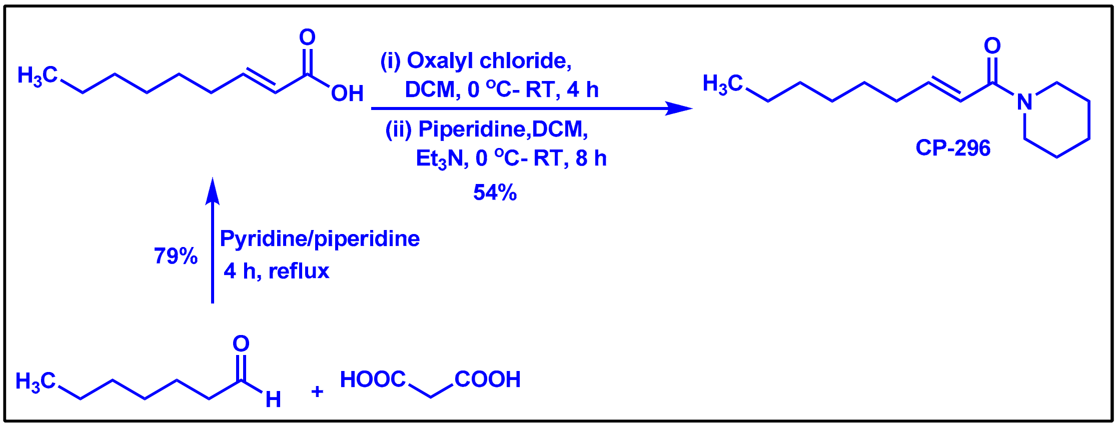

To a solution of heptanal (1.0 g, 8.75 mmol) in pyridine (10 ml) at room temperature, malonic acid (1.82 g, 17.5 mmol) was added and stirred for 10 minutes, to this 0.5ml of piperidine was added and then the reaction mixture was refluxed for 4 h. The progress of the reaction was monitored by TLC (EtOAc). After completion, the reaction mixture was quenched with 1M HCl (20 mL). The product precipitated as white crystalline solid. It was washed with dil. HCl (25 mL), diethyl ether (10 mL) and dried under vacuum to get (E)-cyclohexylacrylic acid in 1.08 g (79% yield).

To a cold (0 ºC) solution of 2-nonenoic acid (1g, 6.4 mmol) in DCM under N_2 atmosphere, oxalyl chloride (1.21g, 9.6 mmol) was added slowly and the reaction mixture was slowly warmed to room temperature and stirred for 4 h. Excess oxalyl chloride was distilled off. Without further purification, the crude product was taken to the next step.

To a stirred solution of piperidine (0.49g, 5.7 mmol**)** and Et_3N (0.87 g, 8.5 mmol) in DCM (20 mL) at 0 °C, the above-synthesized acid chloride in DCM was added dropwise. After the addition, the reaction mass was slowly warmed to room temperature and stirred for 8 h. Progress of the reaction was monitored by TLC (hexane-EtOAc (60:40)). After completion, the DCM layer was washed with sat. $NaHCO_3$ solution (100 mL), water (2 x 75 mL), dried over anhyd. Na_2SO_4 and concentrated under reduced pressure. The obtained crude product was column purified to get the pure product as a pale yellow liquid in 0.77 g (yield 54%).

Physical Characteristics:

Colour and appearance: Pale yellow liquid

Spectral data:

^{1}H NMR (400 MHz, DMSO) δ: 0.88 (3H, t, *J* =8 Hz), 1.26-1.33 (6H, m), 1.41-1.46 (2H, m), 1.54-1.63 (6H, m), 2.19 (2H, dd, *J* =16 Hz, *J* =8 Hz), 3.48-3.60 (4H, br), 6.24 (1H, d, *J* =16 Hz), 6.79-6.86 (1H, m).

^{13}CNMR (100 MHz, DMSO) δ: 14.0, 22.5, 24.6, 25.5, 26.6, 28.3, 28.8, 31.6, 32.5, 43.0, 46.8, 120.2, 145.9, 165.6.

GC-MS (EI$^+$) ***m/z***: 223.15 (M^+).

Synthesis of diconjugated coumaperine derivatives

Preparation of N-Crotonoylpiperidine

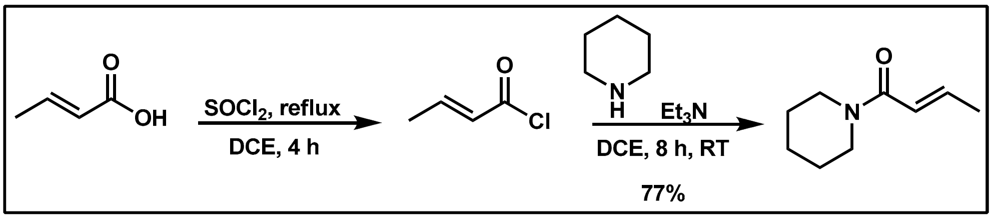

To a stirred solution of crotonic acid (50 g, 0.58 mol) in DCE (150 mL) at 0 °C, $SOCl_2$ (58 mL, 0.669 mol) was added dropwise and refluxed for 4 h. After completion, excess $SOCl_2$ was removed under vacuum. Without further purification, the above-synthesized crotonyl chloride in DCE was added dropwise to a stirred solution of piperidine (58 mL, 0.58 mol**)** in DCE (50 mL) and Et_3N (55 g, 0.4175 mol) at 0 °C. The reaction mass was slowly warmed to room temperature and stirred for 8 h. Progress of the reaction was monitored by TLC (hexane-EtOAc (60:40)). After completion, the DCE layer was washed with sat. $NaHCO_3$ solution (300 mL), water, dried over anhyd. Na_2SO_4 and concentrated under reduced pressure. A pale yellow viscous liquid was obtained in 68.5 g (yield 77%).

Colour and appearance: Pale yellow viscous liquid

Spectral data:

^{1}H NMR (500 MHz, $CDCl_3$) δ: 1.55-1.65 (6H, m), 1.87 (3H, d, *J* = 4 Hz), 3.48-3.59 (4H, m), 6.27 (1H, d, *J* = 16 Hz), 6.79-6.85 (1H, m),

^{13}CNMR (125 MHz, $CDCl_3$) δ: 15.1, 18.1, 24.4, 24.6, 25.5, 26.5, 42.1, 43.0, 47.2, 121.9, 140.6, 165.5.

GC-MS (EI$^+$) ***m/z***: 154.03054 (M^+).

Synthesis of 4-(tetrahydro-2H-pyran-2-yloxy)benzaldehyde

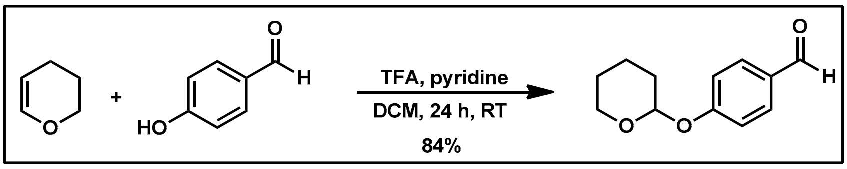

4-Tetrahydropyranyloxybenzaldehyde was prepared according to the literature reported procedure.[4]

To a solution of 4-hydroxybenzaldehyde (5.80 g, 47.5 mmol) and 3,4-dihydro-2H-pyran (6.40 g, 76.1 mmol) in DCM (100 mL), trifluoroacetic acid (0.430 g, 2.50 mmol) was added dropwise and stirred for 24 h. The progress of the reaction was monitored by TLC. After completion, the crude reaction mixture was washed with water (3 x 50 mL), dried over Na_2SO_4 and concentrated under reduced pressure. The residue was washed with 10% NaOH (2 x 50 mL) solution and water to remove any unreacted starting material (4-hydroxybenzaldehyde). The final product was obtained as a dark brown oil in 8.23 g (yield 84%).

Synthesis of (2E,4E)-5-(4-hydroxyphenyl)-1-(piperidin-1-yl)penta-2,4-dien-1-one (Coumaperine, CP).[5]

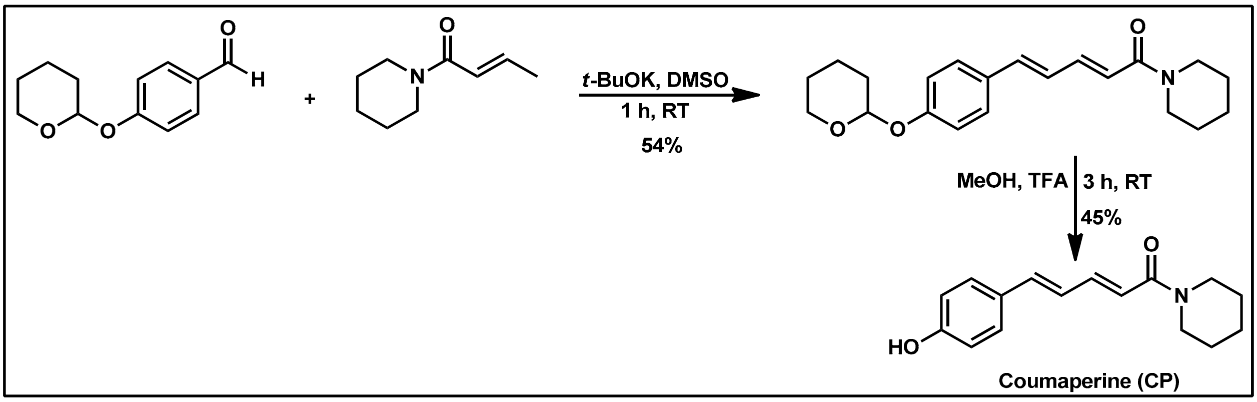

To a stirred solution of *N*-crotonyl piperidine (0.37 g, 2.4 mmol) in DMSO (2 mL), 4-(tetrahydro-2H-pyran-2-yloxy)benzaldehyde (0.5 g, 2.4 mmol) was added and then potassium *tert*-butoxide (0.5 g) in 1.5 mL of DMSO was added slowly and stirred for 1 h. The reaction was monitored by TLC (chloroform-methanol (96:4)). After completion, the reaction mass was quenched with ice-cooled water (100 mL), during which off-white solid precipitated, it was filtered, washed with water, and dried under vacuum to get the product in 0.6 g (yield 54%).

Deprotection[4]

To a solution of tetrahydropyran protected coumaperine (0.6 g, 1.7 mmol) in methanol (8 mL), TFA (0.1 mL) was added dropwise and stirred at room temperature for 3 h. The reaction was monitored by TLC (chloroform-methanol ((96:4)). As the reaction progress, off-white solid precipitated. The reaction mass was cooled to 5 °C, filtered and the off-white solid was washed with water (3 x 30 mL) and dried under vacuum. The obtained crude product was column purified (chloroform-methanol (95:5)) to get the pure product as off-white solid in 0.26 g (yield 45%). The overall yield of the reaction is 24%.

Physical Characteristics:

Colour and appearance: Off-white solid

M.pt: 208 °C - 210 °C (Lit. m.pt = 209 °C - 211 °C)[4]

Spectral data:

^{1}H NMR (500 MHz, DMSO) δ: 1.42-1.58 (6H, m), 3.47-3.50 (4H, m), 6.62 (1H, d, *J* = 15 Hz), 6.74-6.77 (2H, m), 6.83-6.90 (2H, m), 7.20 (1H, dd, *J* =15 Hz, *J* =10 Hz), 7.36 (2H, d, *J* = 10 Hz), 9.95 (1H, s).

^{13}CNMR (125 MHz, DMSO) δ: 24.5, 25.9, 26.9, 40.0, 43.1, 46.6, 116.2, 120.0, 124.6, 127.9, 129.0, 138.7, 142.9, 158.4, 165.1.

IR (KBr) cm^{-1}: 3214, 1632, 1613, 1602, 1513, 1441, 1253, 1005, 843.

GC-MS (EI^{+}) ***m/z***: 257.2132 (M^{+}).

Synthesis of (2E,4E)-5-phenyl-1-(piperidin-1-yl)penta-2,4-dien-1-one (CP-9)[5]

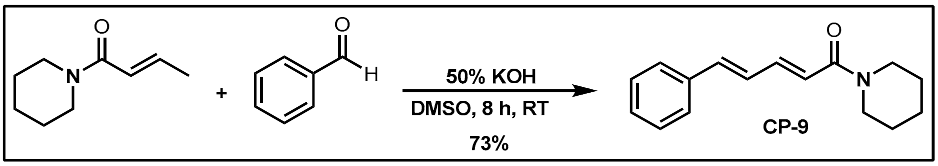

To a solution of *N*-crotonyl piperidine (1.2 g, 7.8 mmol) in DMSO (2 mL), benzaldehyde (0.68 g, 6.42 mmol) was added and then 50% aq. KOH (0.2 mL) solution was added dropwise and stirring continued for 8 h. The progress of the reaction was monitored by TLC (chloroform-methanol (96:4)). After completion, the reaction mixture was quenched with 5% aq. HCl (50 mL) and extracted with DCM (2 x 50 mL), the combined organic layer was washed with water (2 x 50 mL), dried over anhyd. Na_2SO_4 and concentrated under reduced pressure. The obtained crude product

was recrystallized from isopropyl alcohol to get pure product as pale yellow solid in 1.4 g (yield 73%).

Physical Characteristics:

Colour and appearance: Pale yellow solid

M.pt: 95 °C - 97 °C (Lit. m.pt = 95 °C)[5]

Retention factor (R_f) : 0.395 (chloroform-methanol (96:4))

Spectral data:

^{1}H NMR (500 MHz, $CDCl_3$) δ: 1.56-1.67 (6H, m), 3.51-3.64 (4H, m), 6.48 (1H, d, J = 10 Hz), 6.82-6.93 (2H, m), 7.25-7.29 (1H, m), 7.33 (2H, t, J = 10 Hz), 7.42-7.45 (3H, m).

^{13}C NMR (125 MHz, $CDCl_3$) δ: 24.7, 25.7, 26.8, 43.3, 47, 120.9, 127.0, 128.8, 136.5, 138.6, 142.4, 165.4.

IR (KBr) cm^{-1}: 3000, 1636, 1593, 1586, 1372, 1000, 857, 829.

GC-MS (EI^+) *m/z*: 241.2526 (M^+)

Synthesis of (2E,4E)-5-(3,4-dimethoxyphenyl)-1-(piperidin-1-yl)penta-2,4-dien-1-one (CP-10)[5]

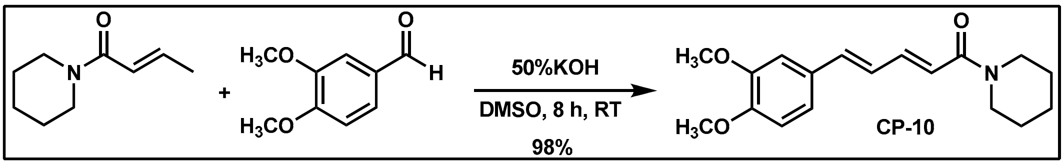

To a solution of *N*-crotonyl piperidine (0.5 g, 3.27 mmol) in DMSO (2 mL), 3,4-dimethoxy benzaldehyde (0.55 g, 3.3 mmol) was added and then 50% aq. KOH (0.2 mL) solution was added dropwise and stirring continued for 8 h. The reaction was monitored by TLC (chloroform-methanol (96:4)). After completion, the reaction mixture was quenched with 5% aq. HCl (50 mL) and extracted with DCM (2 x 50 mL), the combined organic layer was washed with water (2 x 50 mL), dried over anhyd. Na_2SO_4 and concentrated under reduced pressure. The obtained crude product was recrystallized from isopropyl alcohol to get a pure product as pale yellow solid in 0.98 g (yield 98%).

Physical Characteristics:

Colour and appearance: Pale yellow solid

M.pt: 114 °C - 116 °C (Lit. m.pt =115 °C)[6]

Retention factor (R_f) : 0.52 (chloroform/methanol (96:4))

Spectral data:

^{1}HNMR (500 MHz, $CDCl_3$) δ: 1.57-1.65 (6H, m), 3.57(4H, s), 3.87 (3H, s), 3.89 (3H, s), 6.43 (1H, d, J = 15 Hz), 6.77 (2H, d, J = 5 Hz), 6.81-6.83 (1H, m), 6.96-7.00 (2H, m), 7.39-7.44 (1H, m).

^{13}CNMR (125 MHz, $CDCl_3$) δ: 24.7, 26.3, 55.9, 56.0, 109.1, 111.2, 119.8, 120.8, 125.2, 129.7, 138.5, 142.7, 149.1, 149.8, 165.5.

IR (KBr) cm^{-1}: 1637, 1611, 1592, 1460, 1252, 1235.

GC-MS (EI$^+$) ***m/z***: 301.2985 (M^+)

Synthesis of (2E,4E)-1-(piperidin-1-yl)-5-(3,4,5-trimethoxyphenyl)penta-2,4-dien-1-one (CP-27)[5]

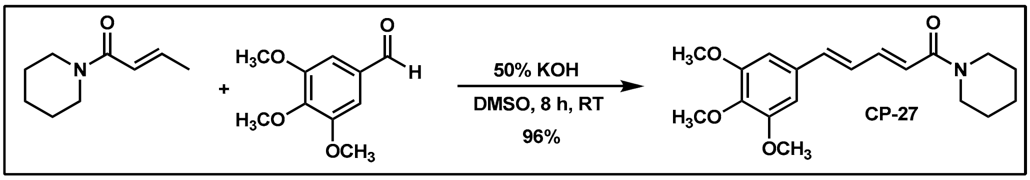

To a solution of *N*-crotonyl piperidine (0.5 g, 3.27 mmol) in DMSO (2 mL), 3,4,5-trimethoxy benzaldehyde (0.64 g, 3.27 mmol) was added and then 50% aq. KOH (0.2 mL) solution was added dropwise and stirring continued for 8 h. The reaction was monitored by TLC (chloroform-methanol (96:4)). After completion, the reaction mixture was quenched with 5% aq. HCl (50 mL) and extracted with DCM (2 x 50 mL), the combined organic layer was washed with water (2 x 50 mL), dried over anhyd. Na_2SO_4 and concentrated under reduced pressure. The obtained crude product was recrystallized from isopropyl alcohol to get the pure product as off-white solid in 1.04 g (yield 96%).

Physical Characteristics:

Colour and appearance: Off-white solid

M.pt: 156 °C - 160 °C

Spectral data:

^{1}HNMR (500 MHz, $CDCl_3$) δ: 1.56-1.68 (6H, m), 3.58 (4H, s), 3.85 (3H, s), 3.87 (6H, s), 6.48 (1H, d, J = 15 Hz), 6.66 (2H, s) 6.74-6.84 (2H, m), 7.39-7.44 (1H, m).

^{13}C NMR (125 MHz, $CDCl_3$) δ: 24.7, 26.3, 56.2, 61.0, 104.1, 120.7, 126.6, 132.2, 138.5, 138.8, 142.4, 153.5, 165.4.

IR (KBr) cm^{-1}: 1637, 1631, 1587, 1549, 1481, 1442, 1256, 1135, 1001.

HRMS (ESI-MS) *m/z*: Calculated for $C_{19}H_{26}NO_4$ $[M+H]^+$ 332.1862, found: 332.1863.

Synthesis of (2E,4E)-5-(4-(dimethylamino)phenyl)-1-(piperidin-1-yl)penta-2,4-dien-1-one (CP-32)[5]

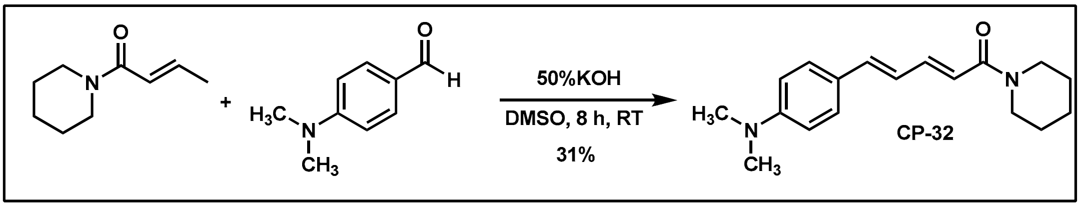

To a solution of *N*-crotonyl piperidine (1 g, 6.54 mmol) in DMSO (2 mL), *N,N*-dimethyl benzaldehyde (1 g, 6.7 mmol) was added and then 50% aq. KOH (0.2 mL) solution was added dropwise and stirring continued for 8 h. The reaction was monitored by TLC (chloroform-methanol (96:4)). After completion, the reaction mixture was quenched with 5% aq. HCl (50 mL) and extracted with DCM (2 x 75 mL), the combined organic layer was washed with water (2 x 100 mL), dried over anhyd. Na_2SO_4 and concentrated under reduced pressure. The obtained crude product was recrystallized from isopropyl alcohol to get the pure product as pale yellow solid in 0.58 g (yield 31%).

Physical Characteristics:

Colour and appearance: Pale yellow solid

M.pt: 129 °C - 131 °C

Spectral data:

^{1}HNMR (500 MHz, $CDCl_3$) δ: 1.56-1.66 (6H, m), 3.53-3.62 (4H, *br* d), 2.97 (6H, s), 6.37 (1H, d, J = 15 Hz), 6.66-6.80 (4H, m), 7.34 (2H, d, J = 15), 7.41-7.46 (1H, m).

^{13}CNMR (125, $CDCl_3$) δ: 24, 25.8, 26.8, 40.4, 43.3, 47, 112.3, 118.1, 122.8, 128.4, 139.3, 143.6, 150.7, 166.9.

IR (KBr) cm^{-1}: 1691, 1659, 1587, 1442, 1256, 1021, 1000, 856.

GC-MS (EI$^+$) ***m/z***: 284.1093 (M^+).

Synthesis of (2E,4E)-5-(4-methoxyphenyl)-1-(piperidin-1-yl)penta-2,4-dien-1-one (CP-38) [5]

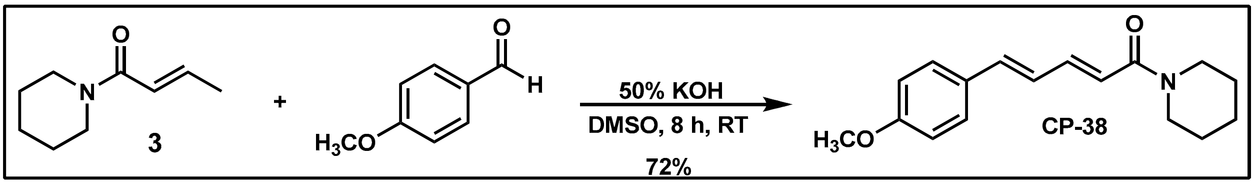

To a solution of *N*-crotonyl piperidine (1.2 g, 7.8 mmol) in DMSO (2 mL), *p*-anisaldehyde (0.96 g 7.1 mmol) was added and then 50% aq. KOH (0.2 mL) solution was added dropwise and stirring continued for 8 h at room temperature. The progress of the reaction was monitored by TLC (chloroform-methanol (96:4)). After completion, the reaction mixture was quenched with aq. HCl (50 mL) and extracted with dichloromethane (2 x 75 mL), the combined organic layer was washed with water (2 x 100 mL), dried over anhyd. Na_2SO_4 and concentrated under reduced pressure. The obtained crude product was recrystallized from isopropyl alcohol to get the pure product as pale yellow solid in 1.36 g (yield 72 %).

Physical Characteristics:

Colour and appearance: Pale yellow solid

M.pt: 95 °C - 97 °C (Lit. m.pt = 95 °C)[5]

Spectral data:

^{1}H NMR (500 MHz, $CDCl_3$) δ: 1.58-1.67 (6H, m), 3.58 (4H, s), 3.82 (3H, s), 6.43 (1H, d, *J* = 15 Hz), 6.78-6.80 (2H, m), 6.87 (2H, d, *J* = 5 Hz), 7.39 (2H, d, *J* = 10 Hz), 7.43-7.45 (1H, m)

^{13}C NMR (125 MHz, $CDCl_3$) δ: 24.8, 55.4, 114.3, 119.7, 125.1, 128.4, 129.4, 138.4, 143.0, 160.1, 165.7.

IR (KBr) cm^{-1}: 1659, 1690, 1587, 1512, 1431, 1257, 1002.

GC-MS (EI$^+$) *m/z*: 271.2821 (M^+)

Synthesis of (2E,4E)-1-(piperidin-1-yl)-5-(2,3,4-trimethoxyphenyl)penta-2,4-dien-1-one (CP-50)[5]

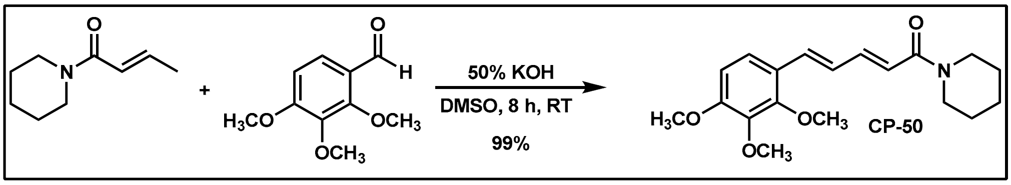

To a solution of *N*-crotonyl piperidine (0.5 g, 3.27 mmol) in DMSO (2 mL), 2,3,4-trimethoxy benzaldehyde (0.64 g, 3.27 mmol) was added and then 50% aq. KOH (0.2 mL) solution was added

dropwise and stirring continued for 8 h. The reaction was monitored by TLC (chloroform-methanol (96:4)). After completion, the reaction mixture was quenched with 5% aq. HCl (50 mL) and extracted with DCM (2 x 50 mL), the combined organic layer was washed with water (2 x 75 mL), dried over anhyd. Na_2SO_4 and concentrated under reduced pressure. The obtained crude product was recrystallized from isopropyl alcohol to get the pure product as pale yellow solid in 1.07 g (yield 99%).

Physical Characteristics:

Colour and appearance: Pale yellow solid

M.pt: 95 °C - 97 °C

Retention factor (R_f) : 0.6 (chloroform-methanol (96:4))

Spectral data:

^{1}HNMR (500 MHz, $CDCl_3$) δ: 1.57-1.67 (6H, m), 3.53-3.64 (4H, *br* d), 3.87 (6H,s),3.88 (3H,s) 6.42-6.45 (1H, m), 6.68 (1H, d, *J* = 10 Hz), 6.83-6.89 (1H, m,), 7.06 (1H, d, *J* = 15 Hz), 7.22 (1H, d, *J* = 10 Hz), 7.42-7.47 (1H, m)

^{13}C NMR (125 MHz, $CDCl_3$) δ: 24.7, 25.7, 26.8, 43.3, 47.0, 56.1, 61.0, 61.2, 107.8, 119.8, 121.5, 123.6, 126.3, 133.2, 143.3, 142.5, 152.2, 154.1, 165.6.

IR (KBr) cm^{-1}: 2935, 1634, 1627, 1589, 1571, 1443, 1293, 1274, 1032, 1011,839, 811.

HRMS (ESI-MS) *m/z*: Calculated for $C_{19}H_{26}NO_4$ $[M+H]^+$ 332.1862, found: 332.1861

Synthesis of (2E,4E)-5-(4-chlorophenyl)-1-(piperidin-1-yl)penta-2,4-dien-1-one (CP-123)[5]

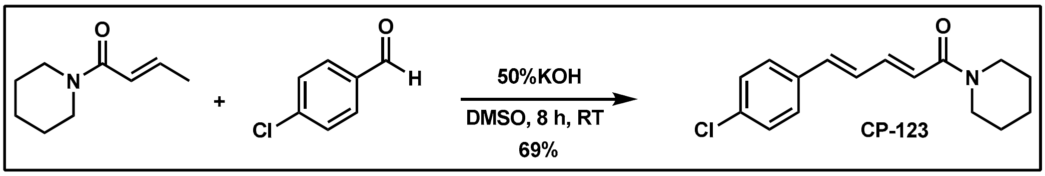

To a solution of *N*-crotonyl piperidine (1 g, 6.54 mmol) in DMSO (2 mL), 4-choloro benzaldehyde (0.94 g, 6.7 mmol) was added and then 50% aq. KOH (0.2 mL) solution was added dropwise and stirring continued for 8 h. The progress of the reaction was monitored by TLC (chloroform-methanol (96:4)). After completion, the reaction mixture was quenched with 5% aq. HCl (50 mL) and extracted with DCM (2 x 50 mL), the combined organic layer was washed with water (2 x 50 mL), dried over anhyd. Na_2SO_4 and concentrated under reduced pressure. The obtained crude product was recrystallized from isopropyl alcohol to get the pure product as pale yellow solid in 1.08 g (yield 69%).

Physical Characteristics:

Colour and appearance: Off-white solid

M.pt: 110 °C - 112 °C

Spectral data:

^{1}HNMR (500 MHz, $CDCl_3$) δ: 1.59-1.68 (6H, m), 3.54-3.65 (4H, *br* d), 6.50 (1H, d, *J* = 15 Hz), 6.79 (1H, d, *J* = 15), 6.87 (1H, d, *J* = 15), 7.30-7.38 (4H, m), 7.41 (1H, dd, *J* = 15, J=10).

^{13}CNMR (125, $CDCl_3$) δ: 24.6, 25.6, 26.7, 40.4, 43.26, 46.9, 121.5, 127.6, 128.0, 128.9, 134.1, 135.01, 136.9, 141.89, 165.23.

IR (KBr) cm^{-1}: 1637, 1580, 1442, 1265, 1022, 1003, 837.

GC-MS (EI^+) *m/z*: 275.1936 (M^+).

Synthesis of (2E,4E)-5-(4-(methylthio)phenyl)-1-(piperidin-1-yl)penta-2,4-dien-1-one (CP-147)[5]

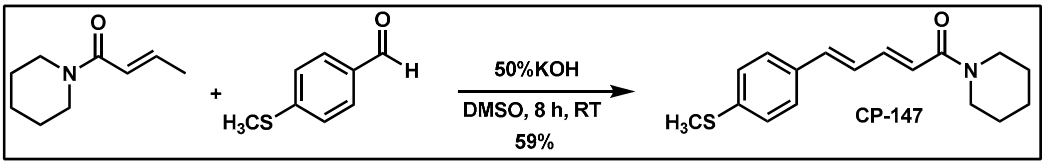

To a solution of *N*-crotonyl piperidine (1 g, 6.54 mmol) in DMSO (2 mL), 4-(methylthio)benzaldehyde (1.01 g, 6.7 mmol) was added and then 50% aq. KOH (0.2 mL) solution was added dropwise and stirring continued for 8 h. The reaction was monitored by TLC (chloroform-methanol (96:4)). After completion, the reaction mixture was quenched with 5% aq. HCl (50 mL) and extracted with DCM (2 x 50 mL), the combined organic layer was washed with water (2 x 50 mL), dried over anhyd. Na_2SO_4 and concentrated under reduced pressure. The obtained crude product was recrystallized from isopropyl alcohol to get the pure product as yellow solid in 1.1 g (yield 59 %).

Physical Characteristics:

Colour and appearance: Pale yellow solid

M.pt: 107.5 °C – 110.2 °C

Spectral data:

^{1}HNMR (500 MHz, $CDCl_3$) δ: 1.60-1.68 (6H, m), 2.5- (3H,s), 3.55-3.65 (4H, *br* d), 6.49 (1H, d, *J* = 15 Hz), 6.80 (1H, d, *J* = 15 Hz), 6.87 (1H, dd, *J* = 15 Hz, *J* = 10 Hz), 7.22 (2H, d, *J* = 5), 7.37 (2H, m, d, *J* = 5), 7.43 (1H, dd, *J* = 15 Hz, *J* = 10 Hz)

^{13}CNMR (125, $CDCl_3$) δ: 15.5, 24.6, 25.6, 43.2, 46.9, 126.3, 133.2, 137.9, 139.3, 142.4, 165.4.
IR (KBr) cm^{-1}: 1635, 1595, 1581, 1432, 1272, 1019, 995, 856.
GC-MS (EI^+) ***m/z***: 287.1532 (M^+).

Synthesis of (2E,4E)-5-(4-fluorophenyl)-1-(piperidin-1-yl)penta-2,4-dien-1-one (CP-154)[5]

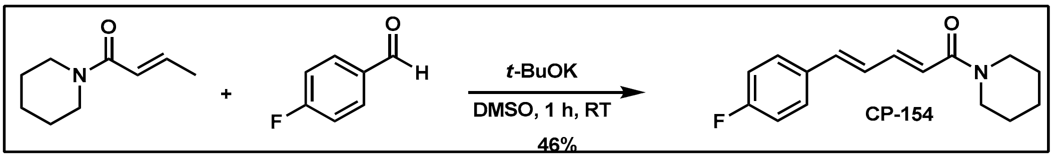

To a solution of *N*-crotonyl piperidine (0.5 g, 3.26 mmol) in DMSO (2 mL), 4-fluorobenzaldehyde (0.5 g 4.03 mmol) was added and then potassium *tert*-butoxide (0.5 g) in 1.5 mL of DMSO was added dropwise and stirred at room temperature for 1 h. The progress of the reaction was monitored by TLC (chloroform-methanol (96:4)). After completion, the reaction mass was quenched with ice-cooled water (50 mL) and extracted with EtOAc (2 x 50 mL), the combined organic layer was washed with 5% aq. HCl solution (50 mL), water (2 x 50 mL), dried over anhyd. Na_2SO_4 and concentrated under reduced pressure. The crude mass was column purified using hexane-EtOAc (90:10) to obtain the pure product as off-white solid in 0.39 g (46% yield).

Physical Characteristics:

Colour and appearance: Pale yellow solid
M.pt: 88.5°C – 91.3 °C

Spectral data:

^{1}HNMR (500 MHz, $CDCl_3$) δ: 1.55-1.66 (6H, m), 3.51-3.63 (4H, *br* d), 6.47 (1H, d, J = 15 Hz), 6.76-6.84 (2H, m), 7.01 (2H, t, J = 10), 7.37-7.42 (3H, m).
^{13}CNMR (125, $CDCl_3$) δ: 24.6, 25.6, 26.7, 43.2, 46.9, 115.6, 120.9, 126.7, 128.5, 132.6, 137.0, 142.1, 165.2
. **IR** (KBr) cm^{-1}: 1638, 1591, 1434, 1264, 1003, 998, 842.
GC-MS (EI^+) ***m/z***: 259.2019 (M^+).

Synthesis of 4-((1E,3E)-5-oxo-5-(piperidin-1-yl)penta-1,3-dienyl)phenyl acetate (CP-158).

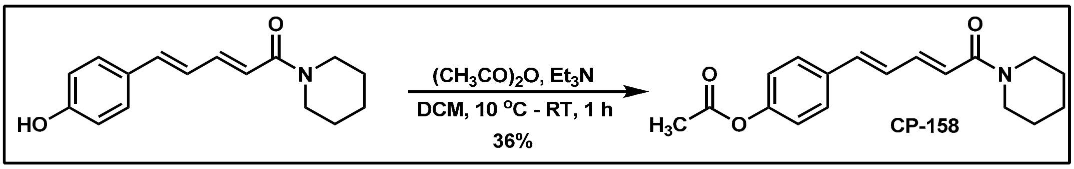

To a solution of coumaperine (0.257 g, 1 mmol) in DCM (10 mL), Et_3N (0.135 g, 1.3 mmol) was added and cooled to 10 °C. To this acetic anhydride (0.136 g, 1.3 mmol) was added slowly and the reaction mass was brought to room temperature and stirred for 1 h. The progress of the reaction was monitored by TLC (chloroform-methanol (96:4)). After completion, the reaction mass was quenched with ice-cooled water (50 mL), extracted with DCM (2 x 50 mL), the combined organic layer was washed with 5% $NaHCO_3$ solution (50 mL), water (2 x 50 mL), dried over anhyd. Na_2SO_4 and concentrated under reduced pressure. The obtained crude product was recrystallized from hexane to get the pure product as off-white solid in 0.11 g (yield 36%).

Physical Characteristics:

Colour and appearance: Off-white solid

M.pt: 133 °C - 135 °C (Lit. m.pt =127 °C -128 °C)[7]

Spectral data:

^{1}HNMR (500 MHz, $CDCl_3$) δ: 1.57-1.68 (6H, m), 3.53-3.64 (4H, *br* d), 2.30 (3H, s), 6.48(1H, d, *J* = 10 Hz), 6.80-6.88 (2H, m), 7.07 (2H, d, *J* = 10 Hz), 7.38-7.45 (3H, m).

^{13}CNMR (125 MHz, $CDCl_3$) δ: 21.2, 24.7, 25.7, 26.8, 43.4, 47.1, 121.2, 122.0, 127.3, 128.0, 134.4, 137.4, 142.2, 150.8, 165.4, 169.4.

IR (KBr) cm^{-1}:1749, 1630, 1593, 1253, 1188, 1011, 910.

HRMS (ESI-MS) *m/z*: Calculated for $C_{19}H_{26}NO_4$ $[M+H]^+$ 300.1599, found: 300.1583.

Synthesis of (2E,4E)-5-(4-(cyclopentyloxy)phenyl)-1-(piperidin-1-yl)penta-2,4-dien-1-one (CP-184)[5]

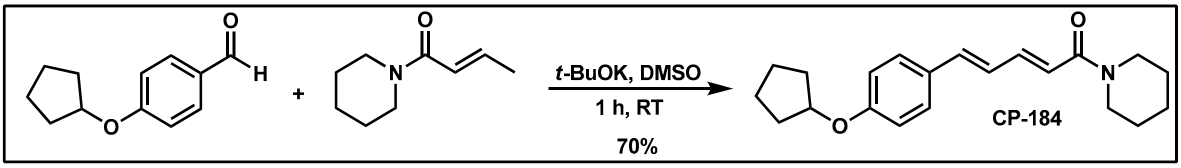

To a solution of *N*-crotonyl piperidine (0.402 g, 2.6 mmol) in DMSO (2 mL), 4-(cyclopentyloxy)benzaldehyde (0.5 g 2.6 mmol) was added and then potassium *tert*-butoxide (0.5 g) in 1.5 mL of DMSO was added dropwise and stirred for 1 h. The progress of the reaction was

monitored by TLC (chloroform-methanol (96:4)). After completion, the reaction mass was quenched with ice-cooled water (50 mL), off-white solid precipitated, it was filtered, washed with water (2 x 50 mL), and dried under vacuum to yield the pure product as off-white solid in 0.6 g (yield 70%).

Physical Characteristics:

Colour and appearance: Off-white solid

M.pt: 112-114 °C

Spectral data:

^{1}HNMR (400 MHz, $CDCl_3$) δ: 1.57-1.90 (14H, m), 3.63-3.54 (4H, *br* d), 4.77 (1H, s), 6.43 (1H, d, J=16 Hz), 6.76-6.84 (4H, m), 7.27-7.45 (3H, m).

^{13}CNMR (100 MHz, $CDCl_3$) δ: 24.1, 24.8, 25.7, 26.9, 32.9, 43.3, 46.9, 79.4, 115.9, 119.6, 124.8, 128.4, 128.9, 138.5, 143.0, 158.8.

IR (KBr) cm^{-1}: 2940, 1636, 1587, 1437, 1252, 1176, 1007, 839, 810.

HRMS (ESI-MS) *m/z*: Calculated for $C_{19}H_{26}NO_4$ $[M+H]^+$ 326.2120, found: 326.2100

Synthesis of (2E,4E)-5-(3,5-dimethoxyphenyl)-1-(piperidin-1-yl)penta-2,4-dien-1-one (CP-193)[5]

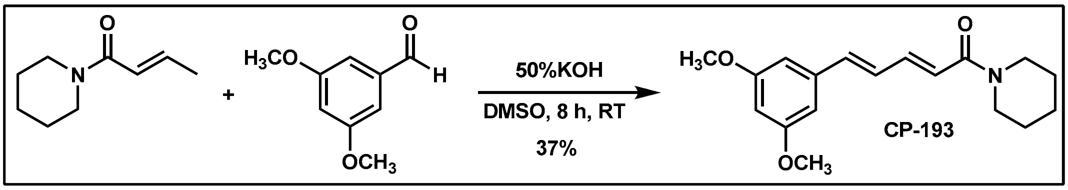

To a solution of *N*-crotonyl piperidine (0.5 g, 3.27 mmol) in DMSO (2 mL), 3,5-dimethoxybenzaldehyde (0.55 g, 3.3 mmol) was added and then 50% aq. KOH (0.2 mL) solution was added dropwise and stirring continued for 8 h. The reaction was monitored by TLC (chloroform-methanol (96:4). After completion, the reaction mixture was quenched with 5% aq. HCl (50 mL) and extracted with DCM (2 x 50 mL), the combined organic layer was washed with water (2 x 50 mL), dried over anhyd. Na_2SO_4 and concentrated under reduced pressure. The obtained crude product was recrystallized from isopropyl alcohol to get the pure product as pale yellow solid in 0.36 g (yield 37%).

<u>**Physical Characteristics:**</u>

Colour and appearance: Pale yellow solid

M.pt: 83 °C - 85 °C

<u>**Spectral data:**</u>

^{1}HNMR (500 MHz, $CDCl_3$) δ: 1.58-1.67 (6H, m), 3.58-3.63(4H, s), 3.81 (6H, s),6.41 (1H, t, *J* = Hz) 6.49 (1H, d, *J* = 16 Hz), 6.60 (2H, d, *J* = Hz), 6.77 (1H, d, *J* = 16 Hz), 6.88 (1H, dd, *J* = 16 Hz, *J* = 12 Hz), 7.41 (1H, dd, *J* = 16 Hz, *J* = 12 Hz) .

^{13}CNMR (125 MHz, $CDCl_3$) δ: 24.6, 101.0, 104.9, 121.2, 127.5, 138.4, 138.5, 142.1, 160.9, 165.3.

IR (KBr) cm^{-1}: 1638, 1591, 1458, 1256, 1155, 1064, 853.

GC-MS (EI^+) *m/z*: 301.220

Synthesis of (2E,4E)-1-(piperidin-1-yl)-5-(2,4,6-trimethoxyphenyl)penta-2,4-dien-1-one (CP-194)[5]

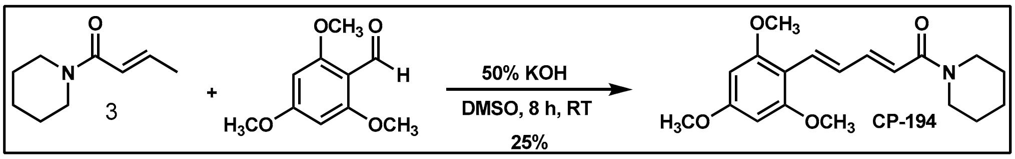

To a solution of *N*-crotonyl piperidine (0.5 g, 3.27 mmol) in DMSO (2 mL), 2,4,6-trimethoxybenzaldehyde (0.64 g, 3.27 mmol) was added and then 50% aq. KOH (0.2 mL) solution was added dropwise and stirring continued for 8 h. The reaction was monitored by TLC (chloroform-methanol (96:4)). After completion, the reaction mixture was quenched with 5% aq. HCl (50 mL) and extracted with DCM (2 x 50 mL), the combined organic layer was washed with water (2 x 50 mL), dried over anhyd. Na_2SO_4 and concentrated under reduced pressure. The crude mass was column purified using hexane-EtOAc (90:10) to get the pure product as pale yellow solid in 0.270 g (yield 25%).

<u>**Physical Characteristics:**</u>

Colour and appearance: Pale-yellow solid; **M.pt**: 110 °C - 112 °C.

<u>**Spectral data:**</u>

^{1}HNMR (500 MHz, $CDCl_3$) δ: 1.56-1.66 (6H, m), 3.53-3.63 (4H, br,d), 3.83 (3H, s), 3.86 (6H, s), 6.12 (2H, s), 6.36 (1H, d, *J* = 15 Hz), 7.19 (1H, d, *J* = 15 Hz), 7.27 (1H, dd, *J* = 10 Hz, *J* = 5 Hz), 7.44 (1H, dd, *J* = 15 Hz, *J* = 10 Hz)

^{13}**C NMR** (125 MHz, $CDCl_3$) δ: 24.7, 25.6, 26.7, 43.1, 46.8, 55.3, 55.7, 90.6, 107.5, 117.5, 128.1, 130.4, 145.9, 160.0, 161.2, 166.0.

IR (KBr) cm^{-1}: 1628, 1432, 1287, 1227, 1103, 1010, 878.

GC-MS (EI$^+$) ***m/z***: 331.1992 (M^+)

Elemental analysis calcd for $C_{19}H_{25}NO_4$: C = 68.66; H = 7.60; N = 4.23. Found: C= 68.61; H= 7.48; N= 4.07.

Synthesis of (2E,4E)-5-(3,4-dihydroxyphenyl)-1-(piperidin-1-yl)penta-2,4-dien-1-one (CP-209).

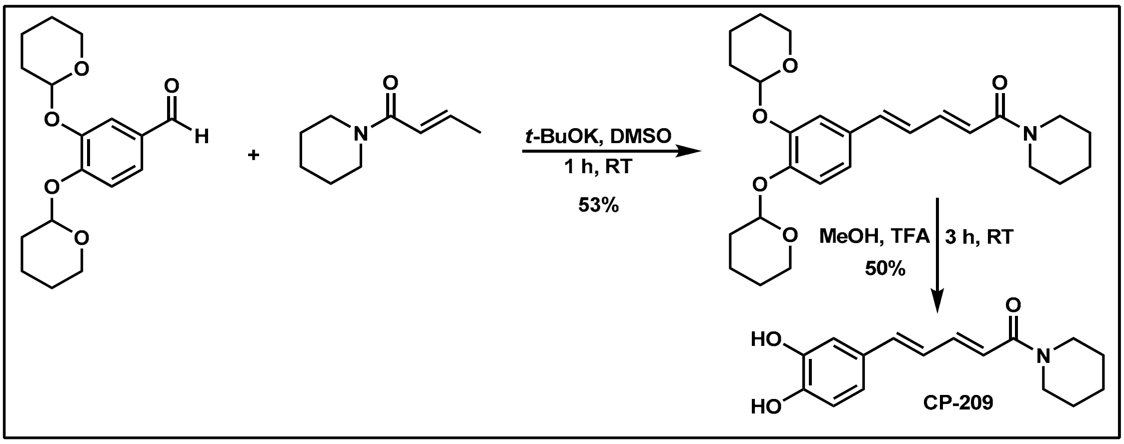

To a stirred solution of *N*-crotonyl piperidine (0.5 g, 3.26 mmol) in DMSO (2 mL), 3,4-bis(tetrahydro-2H-pyran-2-yloxy)benzaldehyde (1.1 g, 3.59 mmol) was added and then potassium *tert*-butoxide (0.54 g) in 1.5 mL of DMSO was added slowly and stirred for 1 h. The reaction was monitored by TLC (chloroform-methanol (96:4)). After completion, the reaction mass was quenched with ice-cooled water (50 mL), extracted with DCM (2 x 50 mL), washed with water (2 x 50 mL), dried over anhyd. Na_2SO_4 and concentrated under reduced pressure to yield the crude product as yellow solid in 0.763 g (yield 53%). Without further purification, it was taken to the next step.

Deprotection[4]

To a methanol solution (8 mL) of the above crude product (0.7 g, 1.58 mmol), TFA (0.1 mL) was added slowly and stirred at room temperature for 3 h. The reaction was monitored by TLC (chloroform-methanol ((96:4)). After completion, methanol was distilled off, the crude mixture was dissolved in EtOAc (50 mL), washed with 5% aq. HCl (50 mL) and then with 5% $NaHCO_3$ (50 mL), dried over anhyd. Na_2SO_4 and concentrated under reduced pressure. The obtained crude

product was column purified (chloroform-methanol (90:10) to get the pure product as yellow solid in 0.21 g (yield 50%). Overall yield 26%.

Deprotection of piperine by BBr_3 method [8]

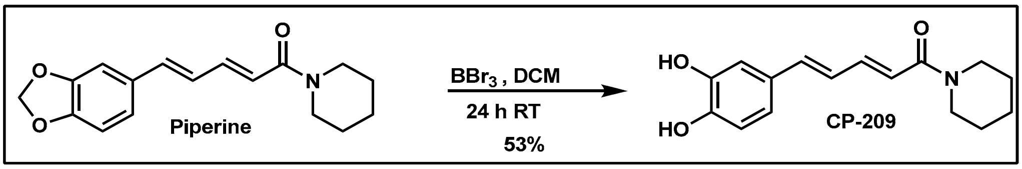

To a solution of piperine (500 mg, 1.75 mmol, 1 eq) in dichloromethane (DCM, 10 mL) at -15 °C, a dichloromethane solution of BBr_3 (0.7 mL, 8.7 mmol, 5 equiv) was added at and slowly warmed to room temperature and stirring continued for 24 h. After the completion, DCM was removed under vacuum to yield yellow solid. It was then with water, DCM, and dried. The pure product was obtained by column purification, 254 mg (53%).

Physical Characteristics:

Colour and appearance: Pale yellow solid

M.pt: 183 °C - 186 °C.

Spectral data:

^{1}H NMR (500 MHz, DMSO) δ: 1.47-1.60 (6H, m), 6.65 (1H, d, *J* = 15 Hz), 6.73 (1H, d, *J* = 10 Hz), 6.78-6.83 (3H, m), 6.93 (1H, s), 7.21 (1H, dd, *J* = 15 Hz, *J* = 10 Hz).

^{13}CNMR (125 MHz, DMSO) δ: 24.6, 25.8, 26.9, 42.9, 114.0, 116.2, 119.9, 120.1, 124.5, 128.4, 139.0, 142.7, 145.9, 147.0, 164.9.

IR (KBr) cm^{-1}: 3453, 3116, 1627, 1612, 1561, 1449, 1253, 1003, 854.

Elemental analysis calcd for $C_{16}H_{19}NO_3$: C = 70.31; H = 7.01; N = 5.12. Found: C = 70.23; H = 7.03; N = 4.77.

Synthesis of (2E,4E)-1-(piperidin-1-yl)-5-(3,4,5-trihydroxyphenyl)penta-2,4-dien-1-one (CP-262-F1, CP-262-F2).

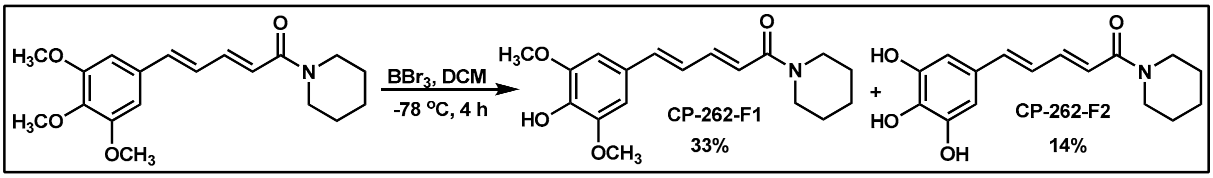

To a solution of **CP-27** 1g (3.0 mmol) in DCM (30 mL) at -78 °C, BBr_3 (26 mmol, 9 mL, 1M DCM solution) was added dropwise under N_2 atm. After the addition, the reaction mixture was slowly warmed to room temperature over 4 h. The progress of the reaction was monitored by TLC. After completion, the reaction mixture was cooled to -20 °C and quenched slowly with ice-cold water (*caution: during quenching violent reaction occurs*). The aqueous phase was extracted with EtOAc (2 x 50 mL), the combined organic layer was dried over Na_2SO_4 and removed under reduced pressure to get the crude product as dark brown solid. The pure product was obtained by column purification as off-white solid, **CP-262-F1** in 0.31 g (33% yield) and as yellow solid, **CP-262-F2** in 0.12 g (14% yield).

Physical Characteristics: (CP-262-F1)

Colour and appearance: Off-white solid

M.pt: 149 °C - 151 °C

Spectral data: (CP-262-F1)

^{1}H NMR (400 MHz, DMSO) δ: 1.42-1.64 (6H, m), 3.51(4H.s, br), 3.79 (6H, s), 6.67 (1H, d, *J* = 16 Hz), 6.81-6.84 (3H, m), 6.99 (1H, dd, *J* = 12 Hz , *J* = 8 Hz), 7.21 (1H, dd, *J* =12 Hz, *J* =8 Hz), 8.71 (1H, br,s).

^{13}CNMR (100 MHz, DMSO) δ: 29.3, 61.5, 109.7, 125.2, 130.1, 132.0, 141.8, 143.7, 147.2, 153.2, 169.5.

IR (KBr) cm^{-1}: 3318, 1637, 1614, 1580, 1515, 1446, 1254, 1022, 858.

GC-MS (EI$^+$) *m/z*: 317.1802 (M$^+$)

Physical Characteristics: (CP-262-F2)

Colour and appearance: yellow solid

M.pt: 190 °C - 192 °C

Spectral data: (**CP-262-F2**)

^{1}H NMR (400 MHz, DMSO) δ: 1.42-1.60 (6H, m), 3.52(4H.s, br), 6.46 (2H, s), 6.65-6.73 (3H, m), 7.17-7.23 (1H, m), 8.50 (1H, s), 9.02 (2H, s).

^{13}CNMR (100 MHz, DMSO) δ: 24.6, 106.5, 120.0, 124.5, 127.3, 135.0, 139.4, 142.7, 146.5, 164.9.

IR (KBr) cm^{-1}: 3514, 3479, 1618, 1572, 1446, 1250, 1028, 854, 836.

Elemental analysis calcd for $C_{16}H_{19}NO_4$: C = 66.42; H = 6.62; N = 4.84. Found: C = 66.55; H= 6.79; N= 4.67.

Synthesis of 4-((1E,3E)-5-oxo-5-(piperidin-1-yl)penta-1,3-dienyl)phenyl mono and di acetate (CP-281-F1)

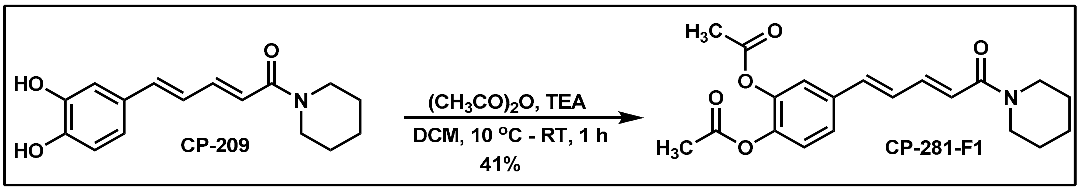

To a solution of CP-209 (0.250 g, 0.9 mmol) in DCM (10 mL), TEA (0.186 g, 1.8 mmol) was added and cooled to 10 °C. To this acetic anhydride (0.186 g, 1.8 mmol) was added slowly and the reaction mass was brought to room temperature and stirred for 1 h. The progress of the reaction was monitored by TLC (chloroform-methanol (96:4)). After completion, the reaction mass was quenched with ice-cooled water (50 mL), extracted with DCM (2 x 50 mL), the combined organic layer was washed with 5% $NaHCO_3$ solution (50 mL), water (2 x 50 mL), dried over anhyd. Na_2SO_4 and concentrated under reduced pressure. The obtained crude product was column purified using hexane-EtOAc (80:20) to obtain the pure product as pale yellow solid (CP-281-F1) in 0.134 g (41% yield).

CP-281-F1

Physical Characteristics:

Colour and appearance: Pale yellow solid

M.pt: 124 °C - 125 °C

Spectral data:

^{1}HNMR (500 MHz, $CDCl_3$) δ: 1.59-1.67 (6H, m), 2.29(6H, d, *J* = 4 Hz), 3.52-3.63 (4H, *br* d), 6.48(1H, d, *J* = 12 Hz), 6.75-6.83 (2H, m), 7.16 (1H, d, *J* = 8 Hz), 7.27-7.31 (2H, m), 7.38 (1H, dd, *J* =16 Hz, *J* =8 Hz),

^{13}CNMR (125 MHz, $CDCl_3$) δ: 20.6, 24.6, 25.6, 26.7, 43.2, 46.9, 121.4, 121.8, 123.6, 125.1, 128.2, 135.5, 136.4, 141.7, 142.0, 142.3, 165.1, 168.4.

IR (KBr) cm^{-1}:3511, 2940, 2857, 1758, 1635, 1566, 1256, 1106, 1013, 912, 849.

GC-MS (EI^+) ***m/z***: 357.10 (M^+).

Isolation of piperine (PIP) from black pepper

Piperine was isolated from black pepper following the literature reported procedure[9]

Black pepper (25 g) was powdered and extracted with hexane (2 x 75 mL) and the hexane layer was kept off. The residue was extracted with $CHCl_3$ (3 x 75 mL). The combined chloroform layer was concentrated under reduced pressure. To the resulted residue, 5N ethanolic KOH solution was added and refluxed for 2 h. The ethanol layer was removed under reduced pressure, the resulted pasty mass was extracted with $CHCl_3$, the $CHCl_3$ layer was washed with water (100 mL), 5% $NaHCO_3$ (50 mL), 5% HCl (50 mL), dried over Na_2SO_4 and concentrated under pressure to get the crude product. The crude product was dissolved in acetone and then hexane was slowly added, during which a white solid precipitated. It was filtered, washed with hexane (3 x 30 mL), and recrystallized from acetone to give to get the pure product as colourless crystals in 0.9 g.

Melting point (129-131°C) and FT-IR spectra of the isolated piperine matched with literature reported value.

Synthesis of triconjugated coumaperine derivatives

Synthesis of N-Sorbyl Piperidine

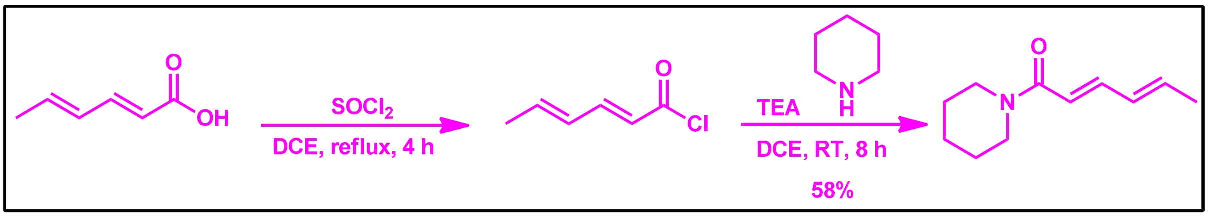

To a solution of sorbic acid (15 g, 0.1339 mol) in DCE (50 mL) at 0 °C, thionyl chloride (14.5 mL, 0.20 mol) was added dropwise and then slowly warmed to room temperature and refluxed for 4 h. Excess thionyl chloride was removed under vacuum. Without further purification, the above-synthesized sorbyl chloride in DCE was added dropwise to a stirred solution of piperidine (13.2 mL, 0.1320 mol) and Et_3N (27 g, 0.2678mol) in DCE (50 mL) at 0 °C. After the addition, the reaction mass was slowly warmed to room temperature and stirred at that temperature for 8 h. The reaction was monitored by TLC (hexane-EtOAc (60:40). After completion, the reaction mass was washed with sat. $NaHCO_3$ solution, water, dried over anhyd. Na_2SO_4 and concentrated under reduced pressure. The crude mass was column purified (using hexane-EtOAc (60:40) as eluent) to get the pure product in 13.9 g (58% yield).

Synthesis of (2E,4E,6E)-7-(4-methoxyphenyl)-1-(piperidin-1-yl)hepta-2,4,6-trien-1-one (CP-102)[5]

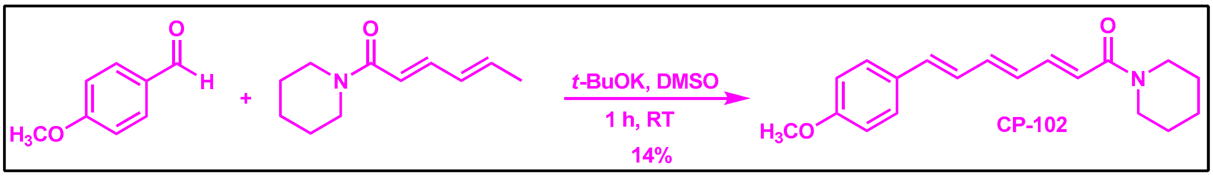

To a solution of *N*-sorbyl piperidine (0.5 g, 2.7 mmol)) in DMSO (2 mL), 4-methoxy benzaldehyde (0.4 g, 2.7 mmol) is added and then potassium *t*ert-butoxide (0.375 g) in 1.5 mL of DMSO is added slowly and stirred for 1 h at room temperature. The reaction is monitored by TLC (chloroform-methanol (96:4)). After completion, the reaction mass is quenched with ice-cooled water and extracted with EtOAc, washed with 5% aq. HCl solution, water, dried over anhyd. Na_2SO_4 and concentrated under reduced pressure. The crude mass is column purified using hexane-EtOAc (90:10) to get the pure product as pale yellow solid in 0.13 g (yield 14%).

Physical Characteristics:

Colour and appearance: Pale yellow solid

M.pt: 131 °C - 133 °C (Lit. m.pt =127 °C -128 °C)

Spectral data:

^{1}H NMR (500 MHz, $CDCl_3$) δ: 1.57-1.65 (6H, m), 3.55 (4H, *br*), 3.80 (3H, s), 6.34-6.42 (2H, m), 6.61 -6.74 (3H, m), 6.85 (2H, d, *J* =10 Hz), 7.35 (3H, q, *J* = 10 Hz).

^{13}C NMR (125 MHz, $CDCl_3$) δ: 24.8, 26.2, 43.5, 46.8, 55.4, 114.2, 119.8, 126.3, 128.1, 129.8, 130.1, 135.3, 139.6, 142.6, 159.8, 165.5.

IR (KBr) cm^{-1}: 3007, 1632, 1452, 1302, 1131, 1116, 1008, 852, 819.

GC-MS (EI$^+$) ***m/z***: 297.2940 (M^+)

Synthesis of (2E,4E,6E)-7-(benzo[d][1,3]dioxol-5-yl)-1-(piperidin-1-yl)hepta-2,4,6-trien-1-one (Pipertine, CP-155)[5]

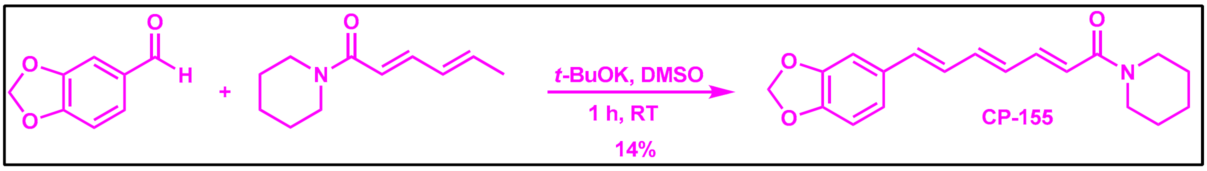

To a solution of *N*-sorbyl piperidine (0.5 g, 2.7 mmol) in DMSO (2 mL), benzo[d][1,3]dioxole-5-carbaldehyde (0.418 g, 2.7 mmol) was added and then potassium *t*ert-butoxide (0.375 g) in 1.5 mL of DMSO was added slowly and stirred for 1 h at room temperature. The reaction progress was monitored by TLC (chloroform-methanol (96:4)). After completion, the reaction mass was quenched with ice-cooled water (50 mL) and extracted with EtOAc (2 x 50 mL), the combined organic layer was washed with 5% aq. HCl (2 x 30 mL), water (2 x 50 mL), dried over anhyd. Na_2SO_4 and concentrated under reduced pressure. The crude mass was column purified using hexane-EtOAc (95:5) to get the pure product as pale yellow solid in 0.12 g (yield 14%).

Physical Characteristics:

Colour and appearance: Pale yellow solid

M.pt: 145 °C - 147 °C (Lit. m.pt = 146 °C -149 °C)[8]

Spectral data:

^{1}HNMR (500 MHz, $CDCl_3$) δ: 1.55-1.67 (6H, m), 3.51-3.62 (4H, *br* d), 5.96 (2H, s), 6.36-6.43(2H, m), 6.58-6.70 (3H, m), 6.76 (1H, d, J = 10 Hz), 6.84-6.86 (1H, m) 6.95 (1H, d, J = 5 Hz), 7.35 (1H, dd, J =15 Hz, J =10 Hz).

^{13}CNMR (125 MHz, $CDCl_3$) δ: 24.7, 25.7, 26.8, 43.4, 46.8, 101.2, 105.5, 108.5, 120.1, 122.1, 126.7, 130.5, 131.5, 135.3, 139.2, 142.4, 147.8, 148.2, 165.5.

IR (KBr) cm^{-1}: 3011, 1632, 1508, 1361, 1223, 1188, 1008, 954, 857, 801.

GC-MS (EI$^+$) ***m/z***: 311.3261 (M^+)

Synthesis of (2E,4E,6E)-7-(3,4-dihydroxyphenyl)-1-(piperidin-1-yl)hepta-2,4,6-trien-1-one (CP-239).

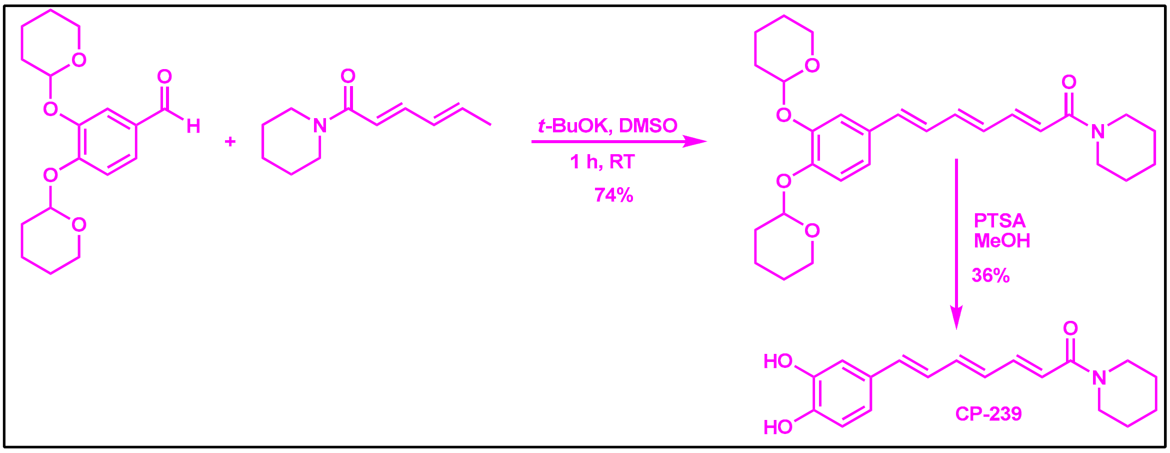

To a stirred solution of *N*-sorbyl piperidine (0.5 g, 2.8 mmol) in DMSO (2 mL), 3,4-bis(tetrahydro-2H-pyran-2-yloxy)benzaldehyde (1.1 g, 3.5 mmol) was added and then potassium *tert*-butoxide (0.54 g) in 1.5 mL of DMSO was added slowly and stirred at room temperature for 1 h. The reaction was monitored by TLC (chloroform-methanol (96:4)). After completion, the reaction mass was quenched with ice-cooled water (50 mL), extracted with DCM (2 x 50 mL), the combined organic layer was washed with water (2 x 50 mL), dried over anhyd. Na_2SO_4 and concentrated under reduced pressure to get the bistetrahydropyran protected **CP-239** in 0.96 g (yield 74 %).

Deprotection[4]

To a solution of bistetrahydropyran protected **CP-239** (0.96 g, 2.0 mmol) in methanol (8 mL), TFA (0.1 mL) was added slowly and stirred at room temperature for 3 h. The reaction was monitored by TLC (chloroform-methanol ((96:4)). After completion, methanol was distilled off, the crude mixture was dissolved in EtOAc (50 mL), washed with 5% aq. HCl (50 mL) and then with 5% $NaHCO_3$ (50 mL), dried over anhyd. Na_2SO_4 and concentrated under reduced pressure to get the crude product. The pure product was obtained after column purification (chloroform-methanol (90:10) as yellow solid in 0.22 g (yield 36%). Overall yield 26%.

Physical Characteristics:

Colour and appearance: Yellow solid

M.pt: 181 °C - 183 °C

Spectral data:

^{1}H NMR (400 MHz, DMSO) δ: 1.43-1.6 (6H, m), 6.46-6.53 (1H, m), 6.59-6.64 (2H, m), 6.74-6.68-6.76 (3H, m), 6.80 (1H, d, J = 8 Hz), 7.16 (1H, dd, J =16 Hz, J =12 Hz), 9.27 (2H, br).

^{13}CNMR (100 MHz, DMSO) δ: 24.5, 113.8, 114.4, 118.7, 119.6, 120.1, 120.5, 122.3, 125.8, 127.2, 128.8, 129.7, 130.0, 164.6, 164.9.

IR (KBr) cm^{-1}: 3462, 3106, 1632, 1613, 1584, 1561, 1439, 1264, 1021, 803.

Elemental analysis calcd for $C_{18}H_{21}NO_3$: C = 72.22; H = 7.07; N = 4.68. Found: C = 72.26; H = 7.23; N = 4.52.

References

[1] Huang H, Morgan CM, Asolkar RN, Koivunen ME, Marrone PG. *J. Agr. Food Chem*. **2010**, *17,* 9994.

[2] Fang F, Li Y, Tian SK. *Eur. J. Org. Chem*. **2011**, *6,* 1084.

[3] Pardin C, Pelletier JN, Lubell WD, Keillor JW. *J. Org. Chem*. **2008**, *73,* 5766.

[4] Bodipati N, Palla SR, Peddinti RK. *Indian. J. Chem. Sect. B*. **2012**, *51B*, 356-361.

[5] Dehmlow EV, Shamout AR. *J. Chem. Res. Mini*. **1981**, *4*, 1178.

[6] Banerji A, Banerjee T, et al. *J. Indian. Chem. Soc.* **2002**, *79*, 876.

[7] Nakatani N, Inatani R, Fuwa H. *Agric. Biol. Chem*. **1980**, *44*, 2831.

[8] Venkatasamy R, Faas L, Young AR, Raman A, Hider RC. *Bioorg. Med. Chem*. **2004**. *12,* 1905.

[9] Shingate PN, Dongre PP, Kannur DM. *Int. J. Pharm. Sci. Res.* **2013***,* 4, 3165.

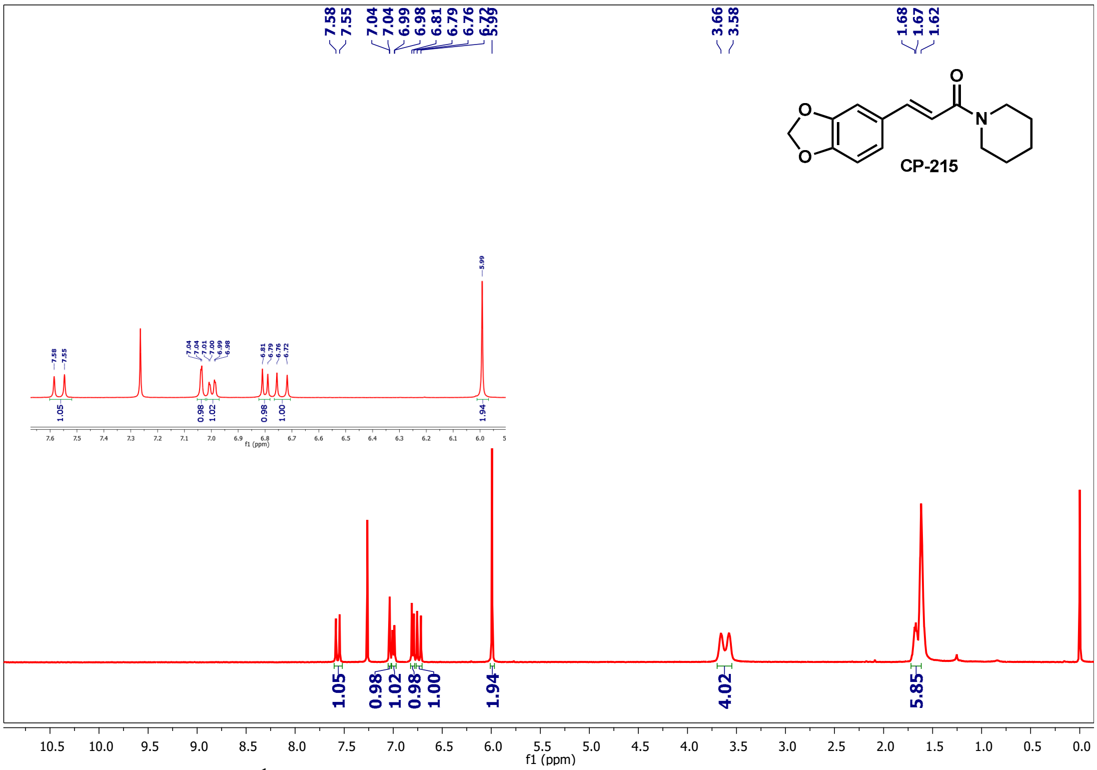

Figure 1: 400 MHz ^{1}H NMR spectrum of **CP-215** in $CDCl_3$

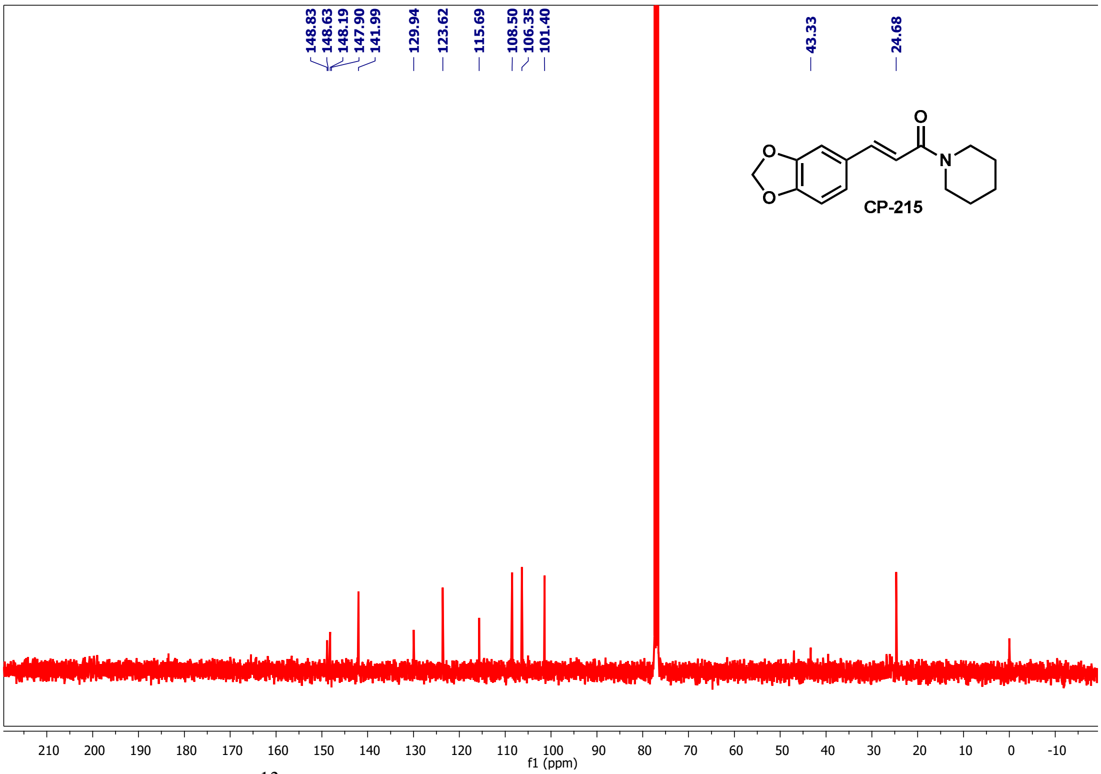

Figure 2: 100 MHz ^{13}C NMR spectrum of **CP-215** in $CDCl_3$

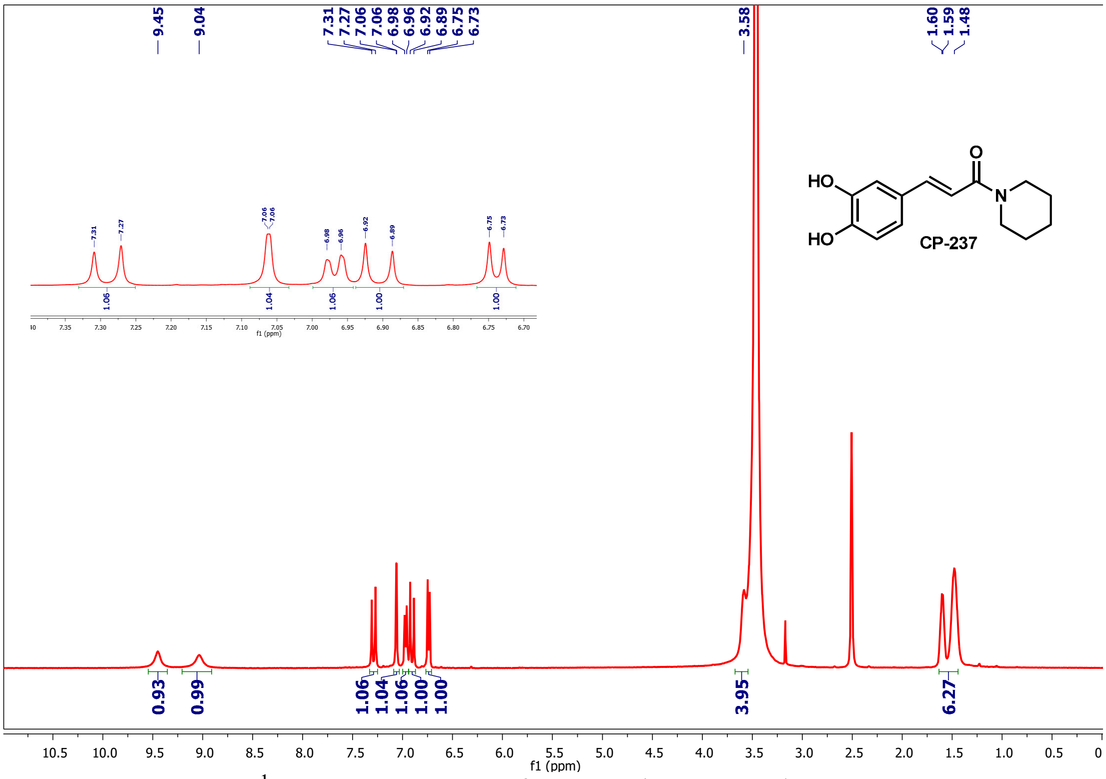

Figure 3: 400 MHz ^{1}H NMR spectrum of **CP-237** in DMSO-d_6

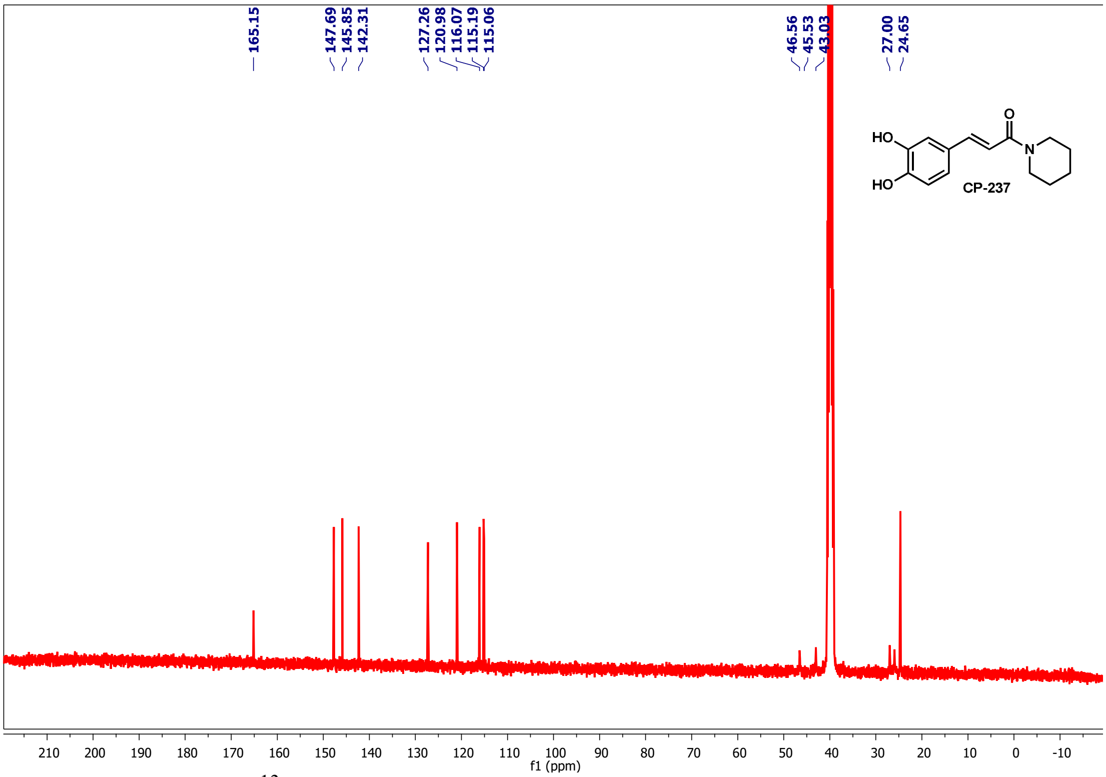

Figure 4: 100 MHz ^{13}C NMR spectrum of **CP-237** in DMSO-d6

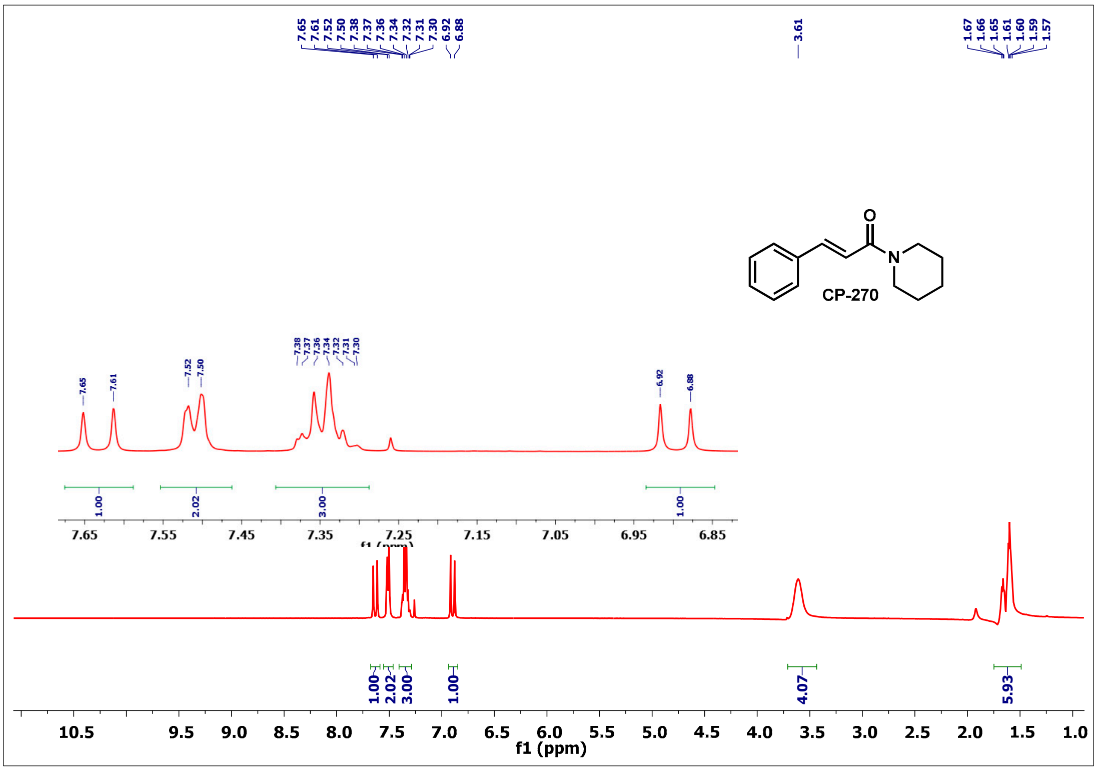

Figure 5: 400 MHz ^{1}H NMR spectrum of **CP-270** in $CDCl_3$

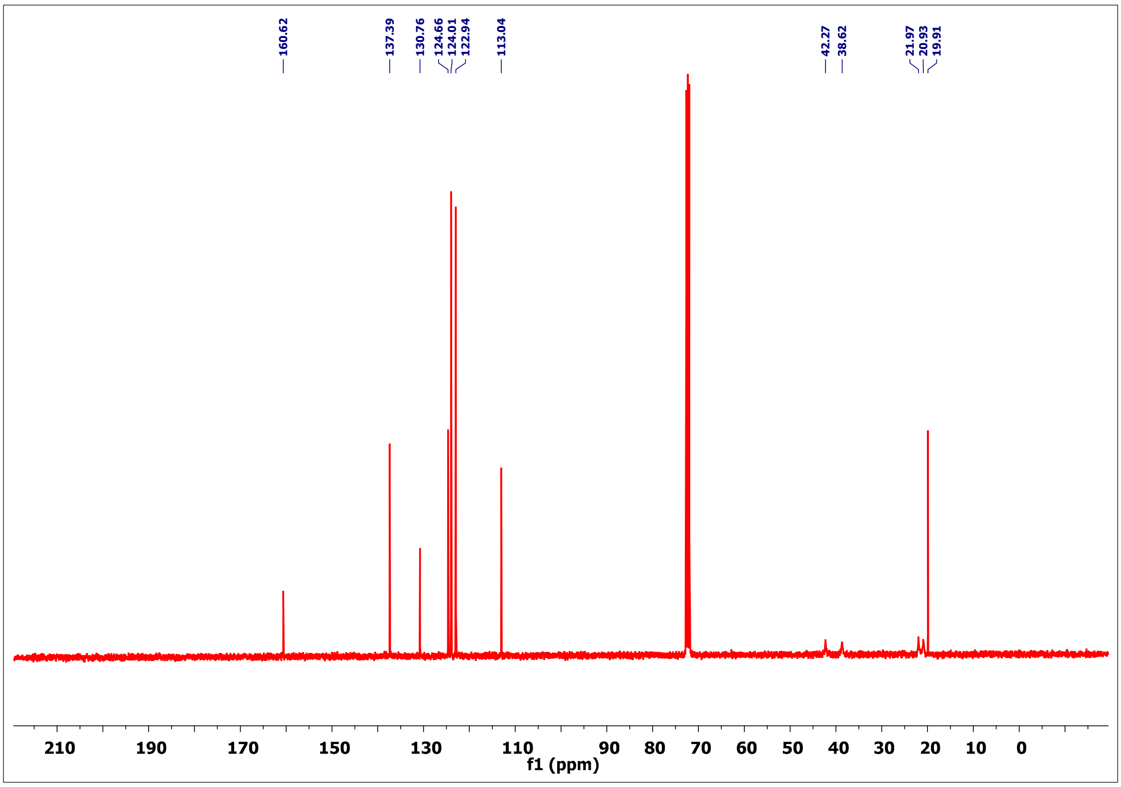

Figure 6: 100 MHz ^{13}C NMR spectrum of **CP-270** in $CDCl_3$

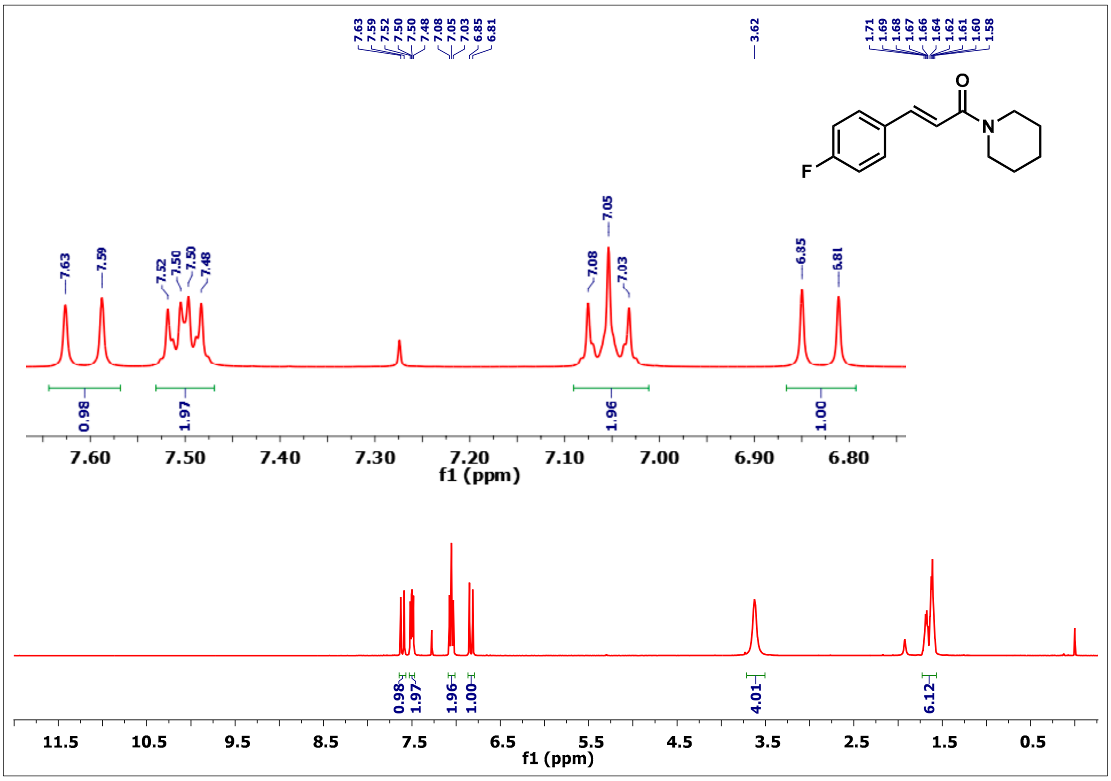

Figure 7: 400 MHz ^{1}H NMR spectrum of **CP-273** in $CDCl_3$

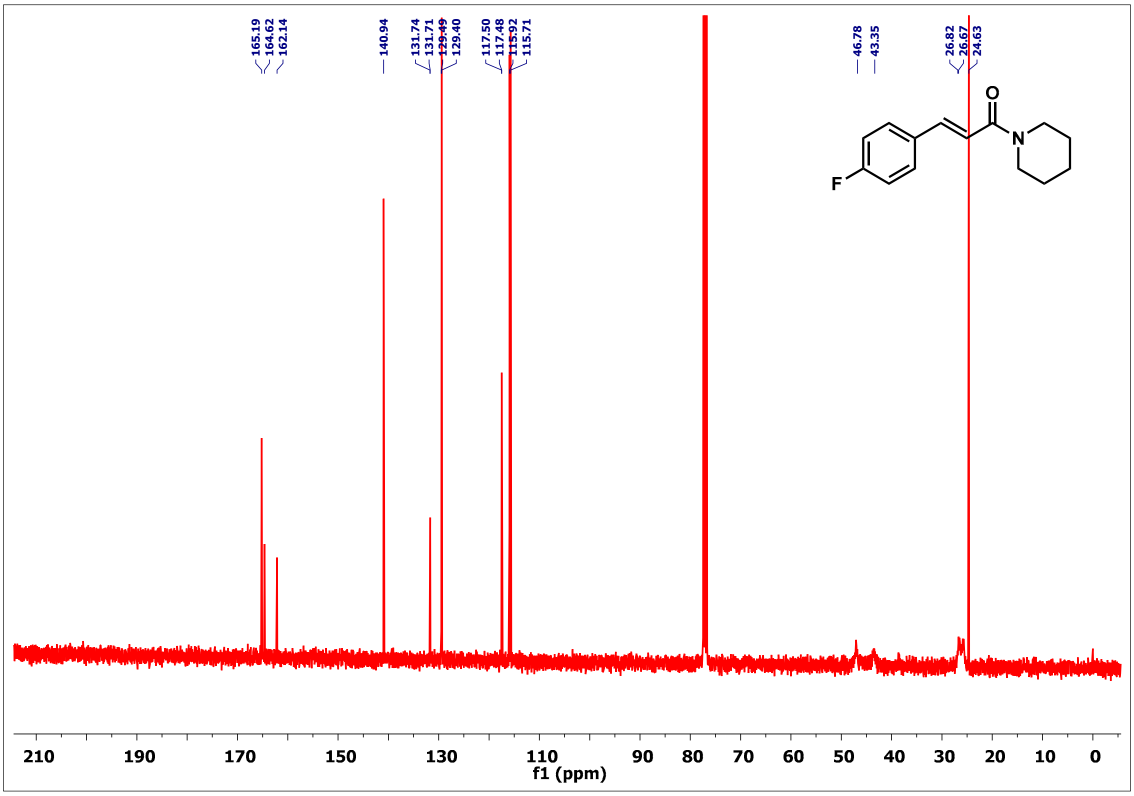

Figure 8: 100 MHz ^{13}C NMR spectrum of **CP-273** in $CDCl_3$

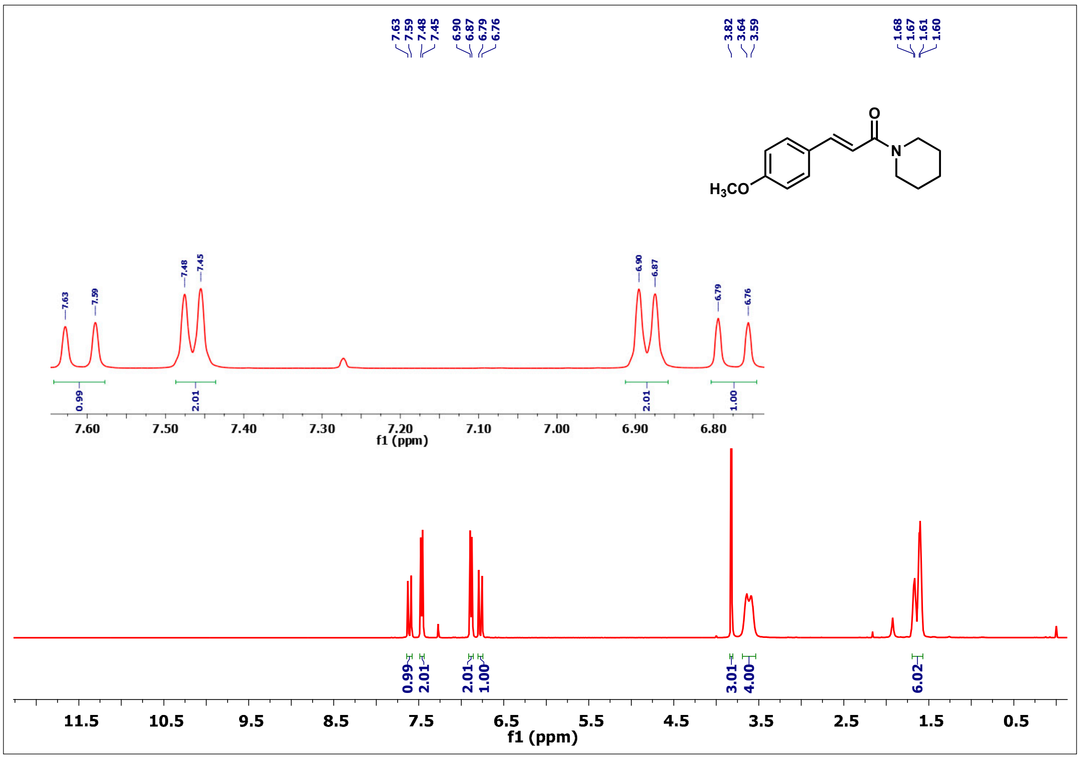

Figure 9: 400 MHz ^{1}H NMR spectrum of **CP-282** in $CDCl_3$

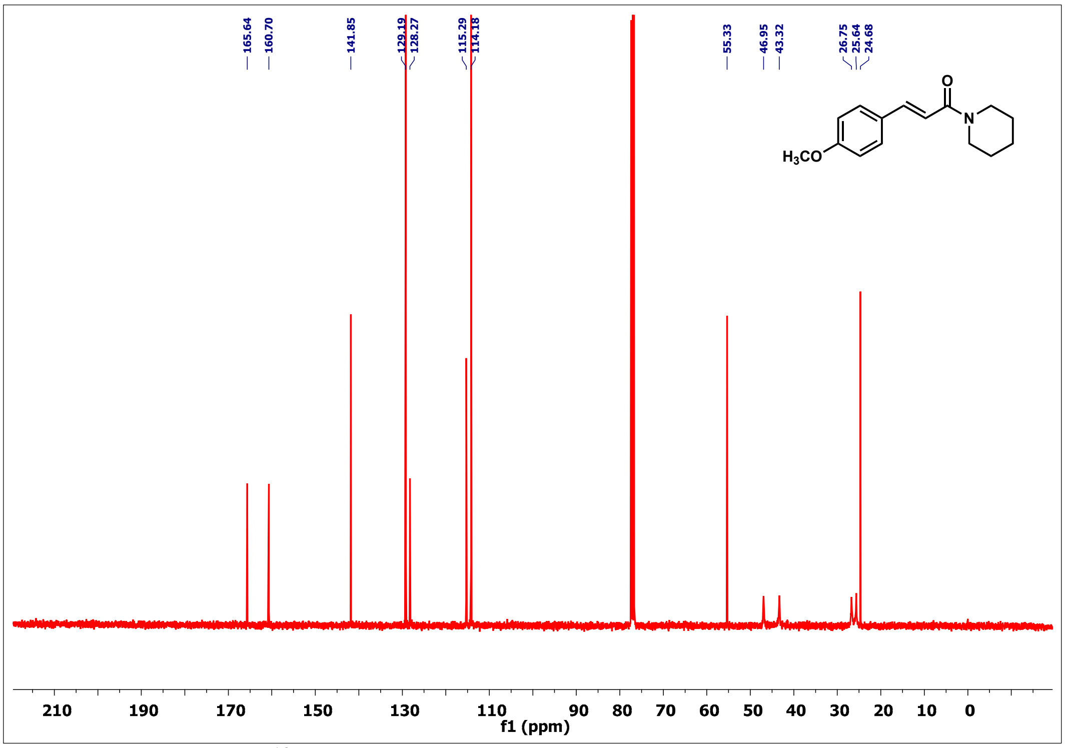

Figure 10: 100 MHz ^{13}C NMR spectrum of **CP-282** in $CDCl_3$

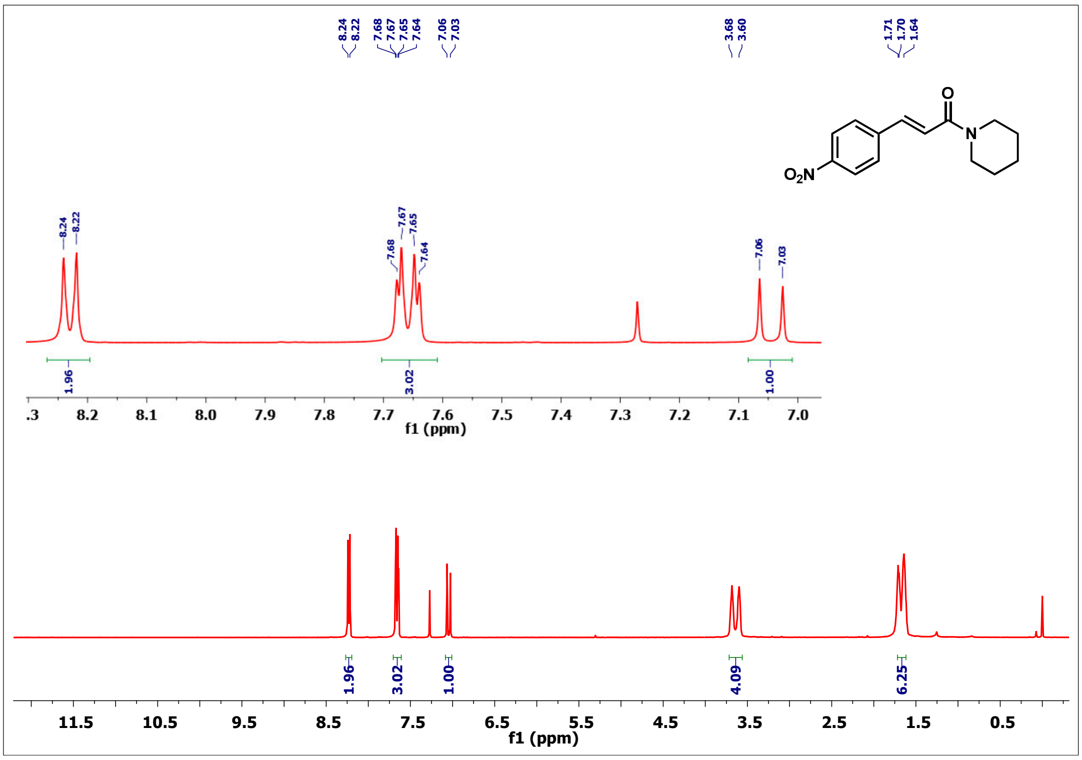

Figure 11: 400 MHz ¹H NMR spectrum of **CP-286** in $CDCl_3$

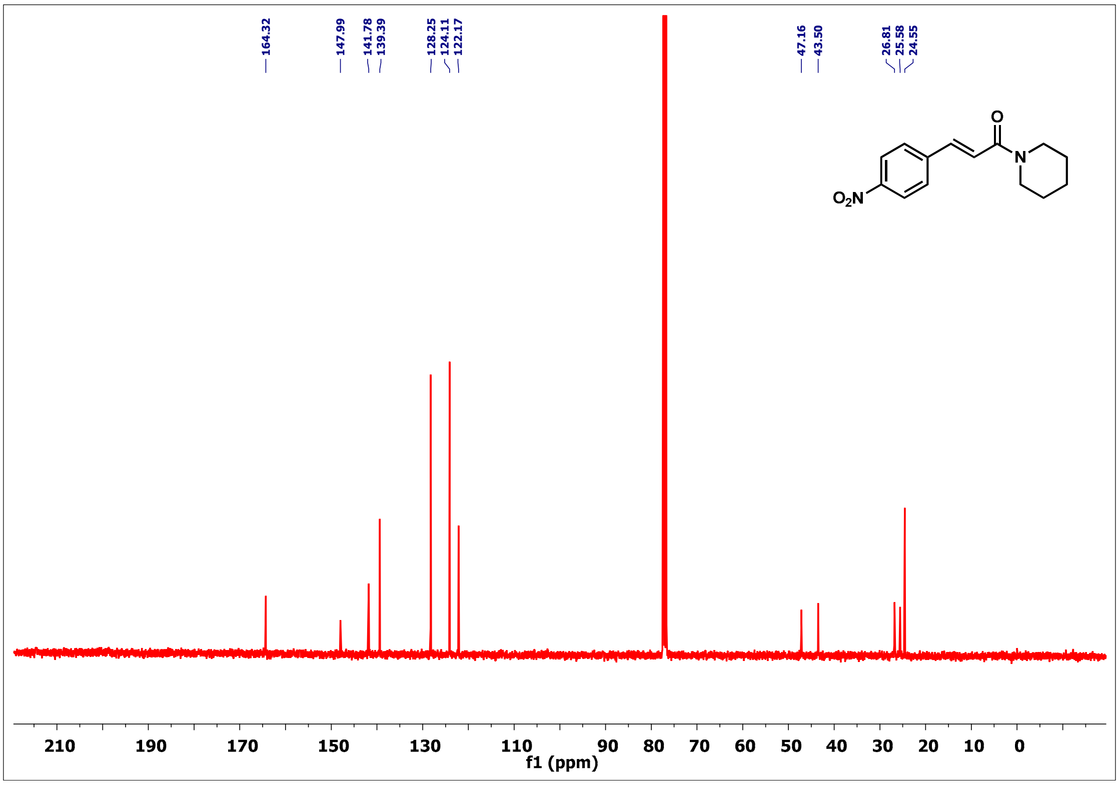

Figure 12: 100 MHz ¹³C NMR spectrum of **CP-286** in $CDCl_3$

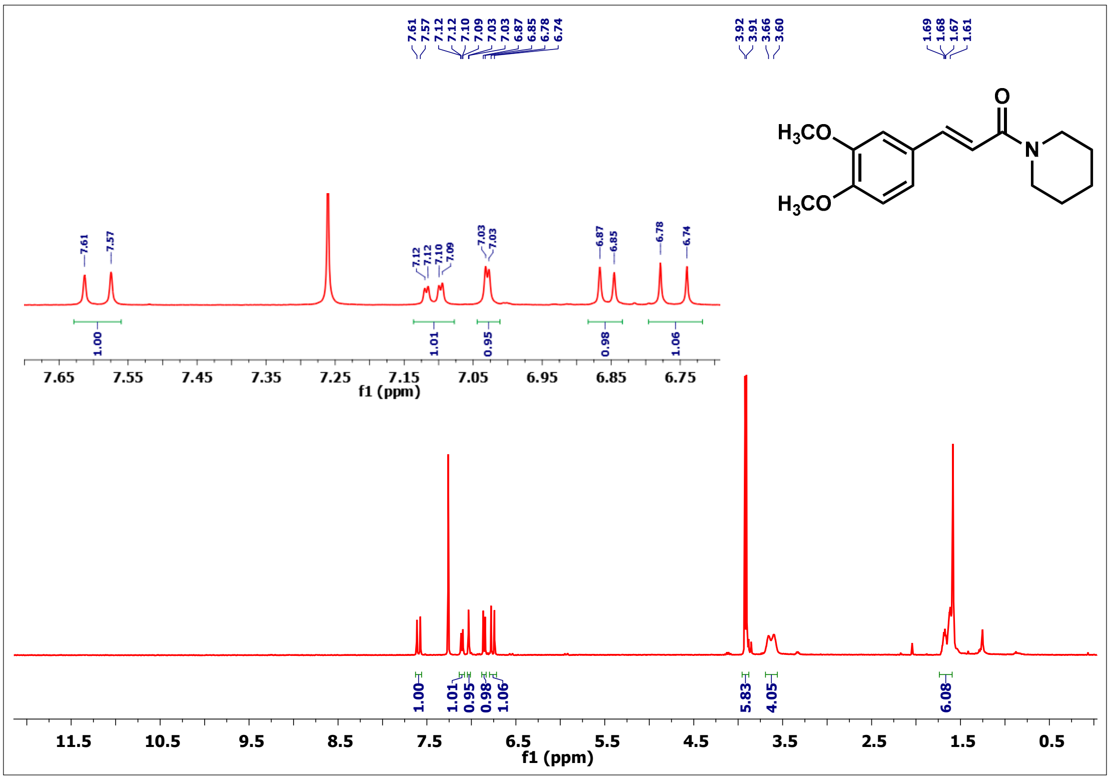

Figure 13: 400 MHz ^{1}H NMR spectrum of **CP-289** in $CDCl_3$

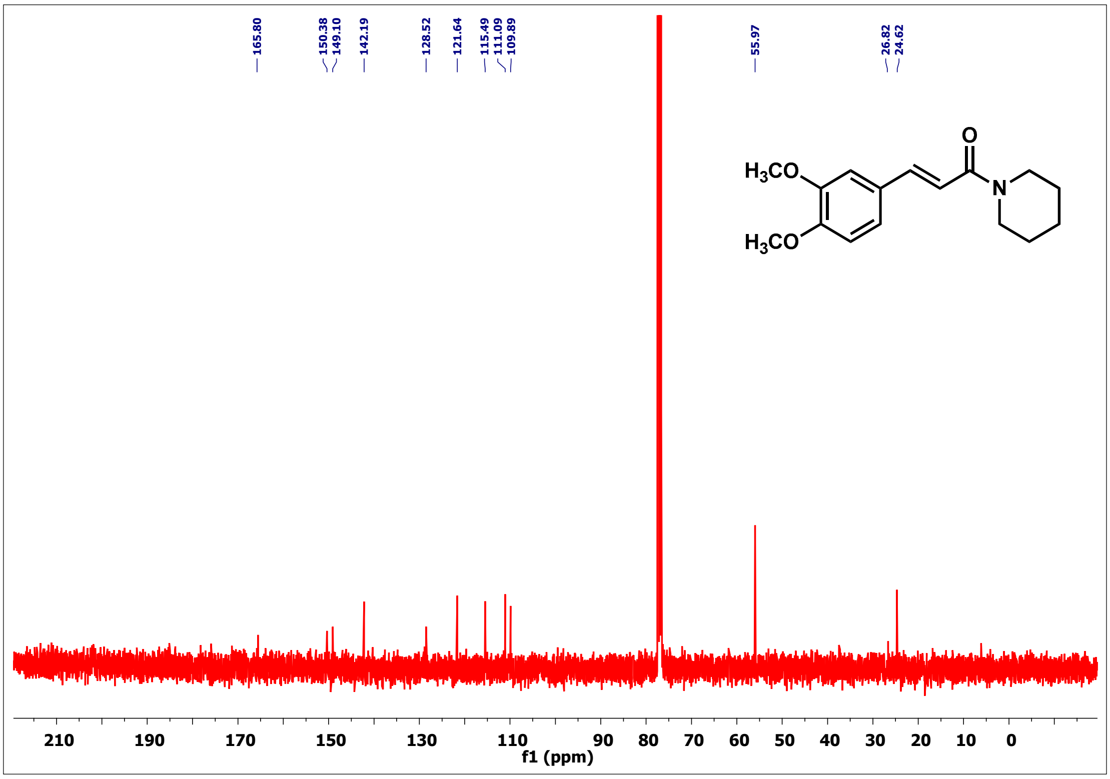

Figure 14: 100 MHz ^{13}C NMR spectrum of **CP-289** in $CDCl_3$

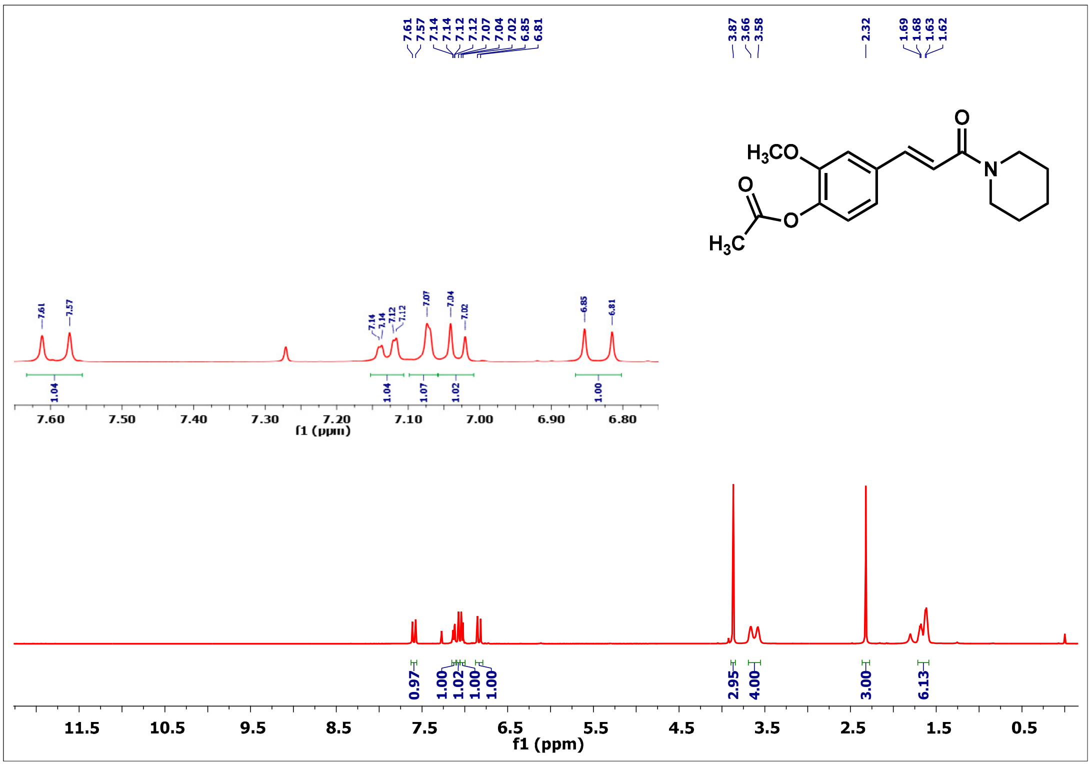

Figure 15: 400 MHz ^{1}H NMR spectrum of **CP-291** in $CDCl_3$

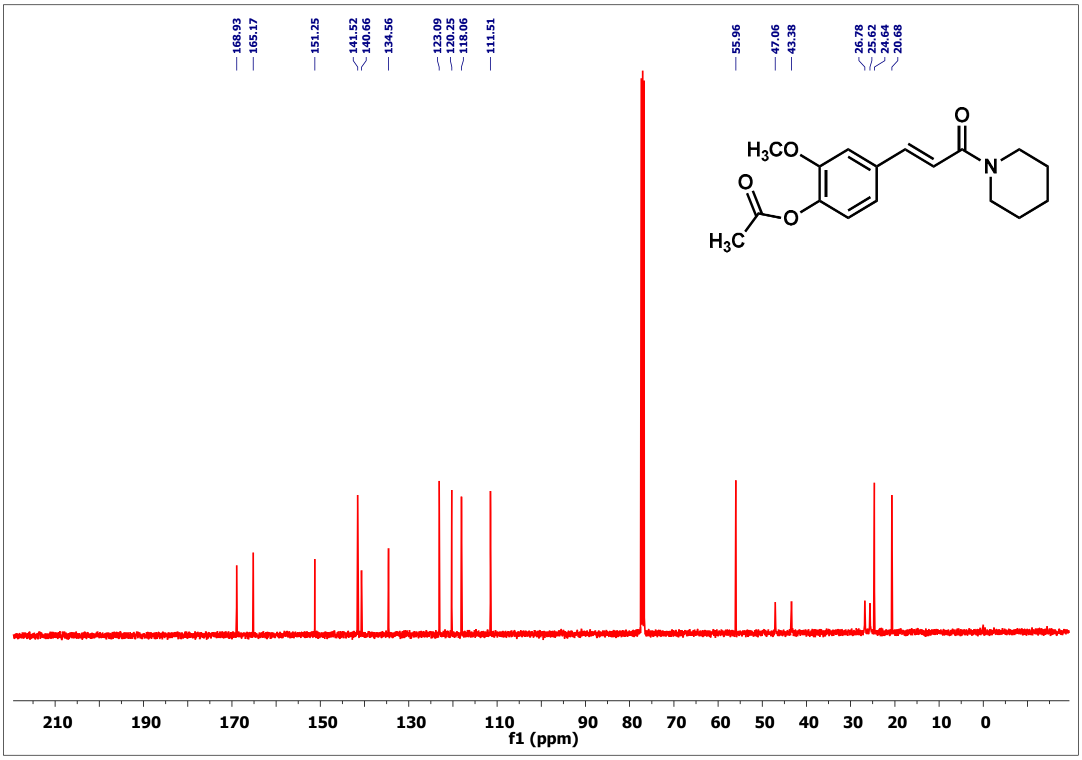

Figure 16: 100 MHz ^{13}C NMR spectrum of **CP-291** in $CDCl_3$

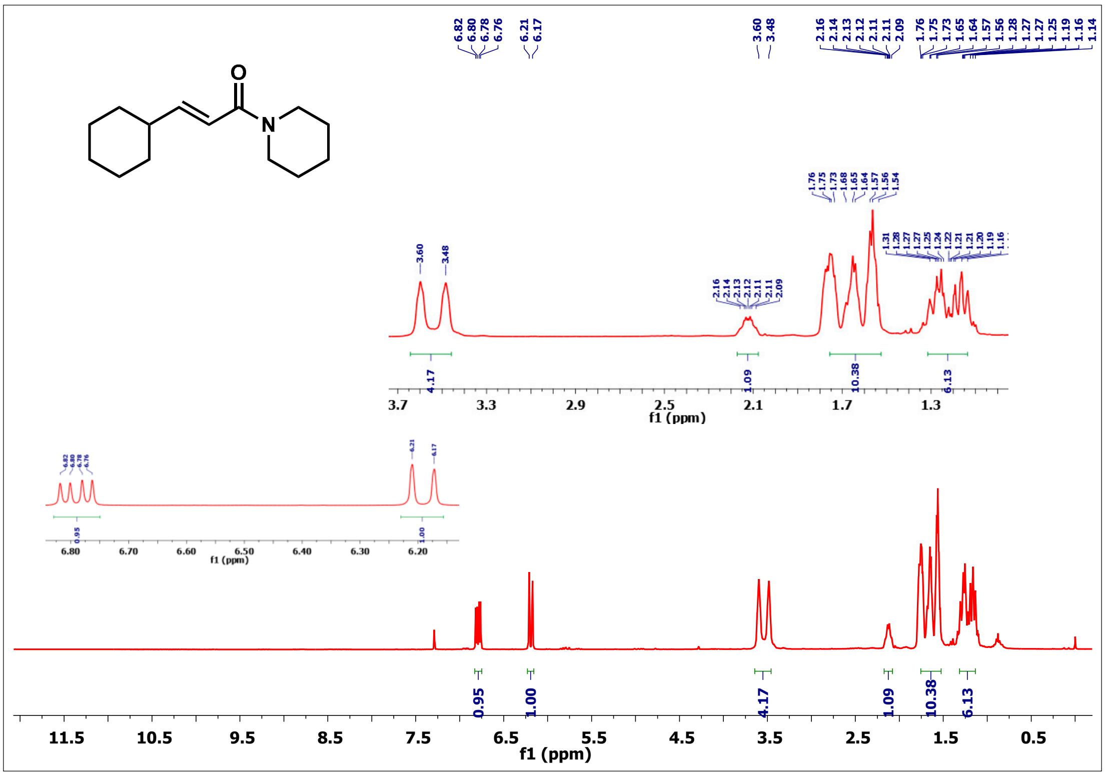

Figure 17: 400 MHz ^{1}H NMR spectrum of **CP-295** in $CDCl_3$

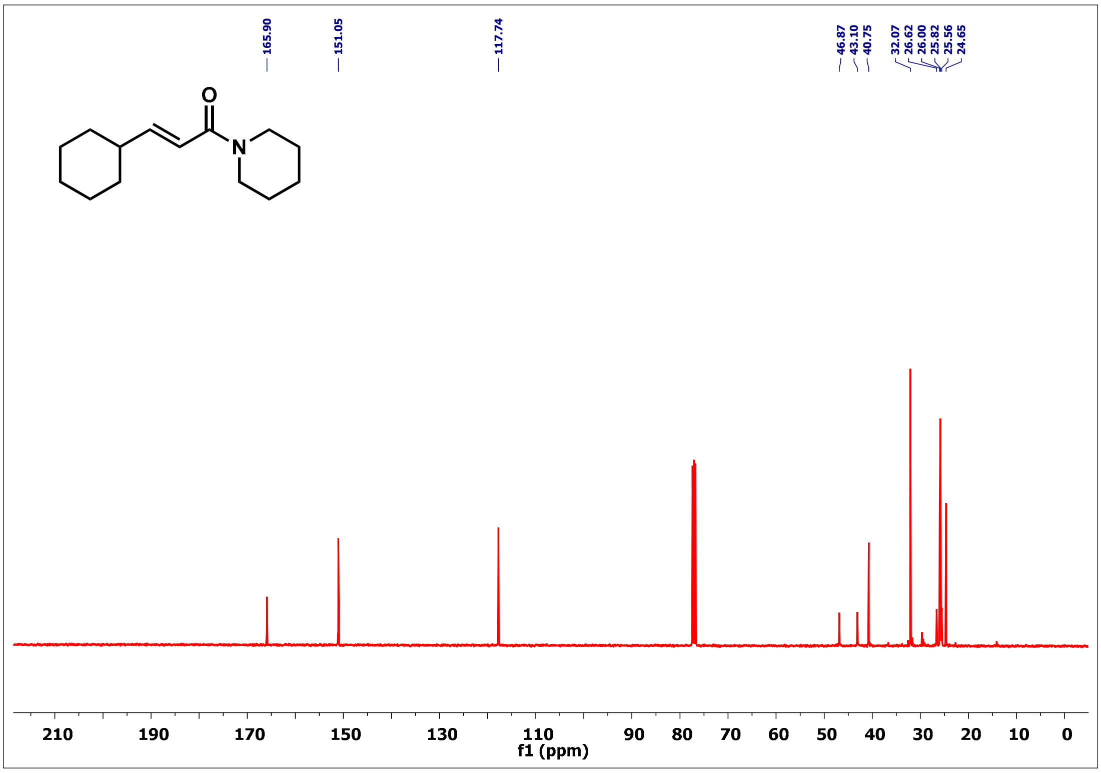

Figure 18: 100 MHz ^{13}C NMR spectrum of **CP-295** in $CDCl_3$

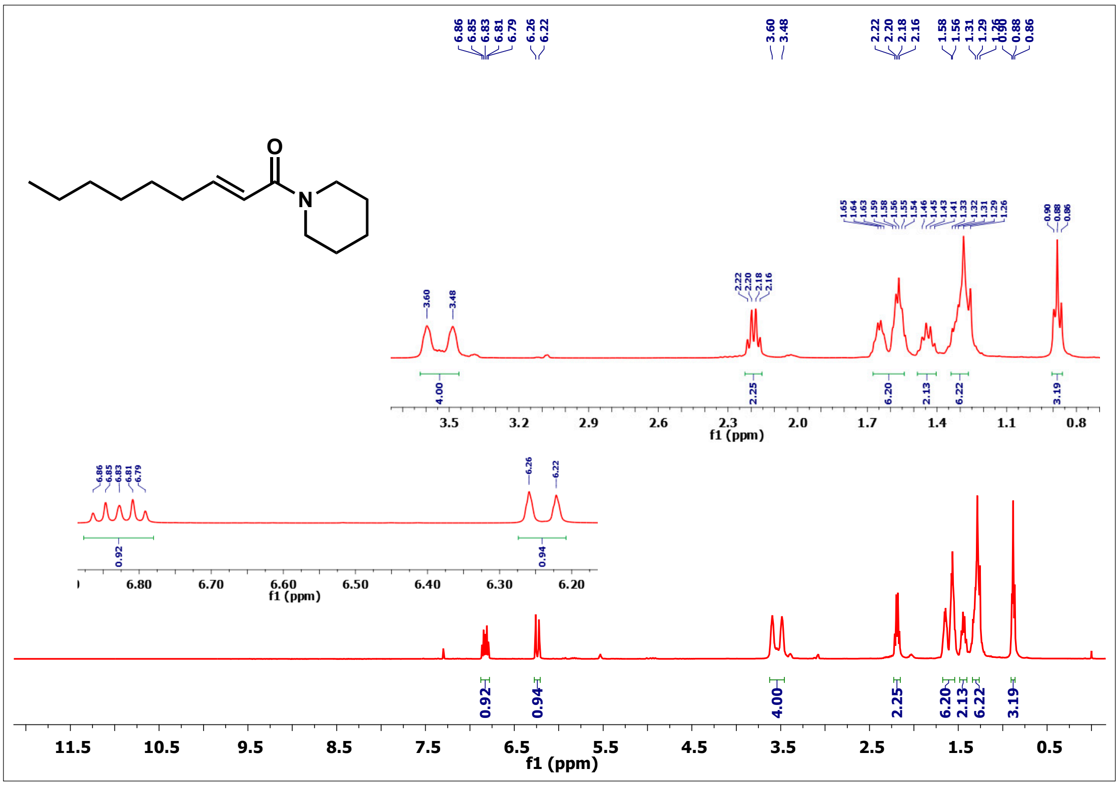

Figure19: 400 MHz ^{1}H NMR spectrum of **CP-296** in $CDCl_3$

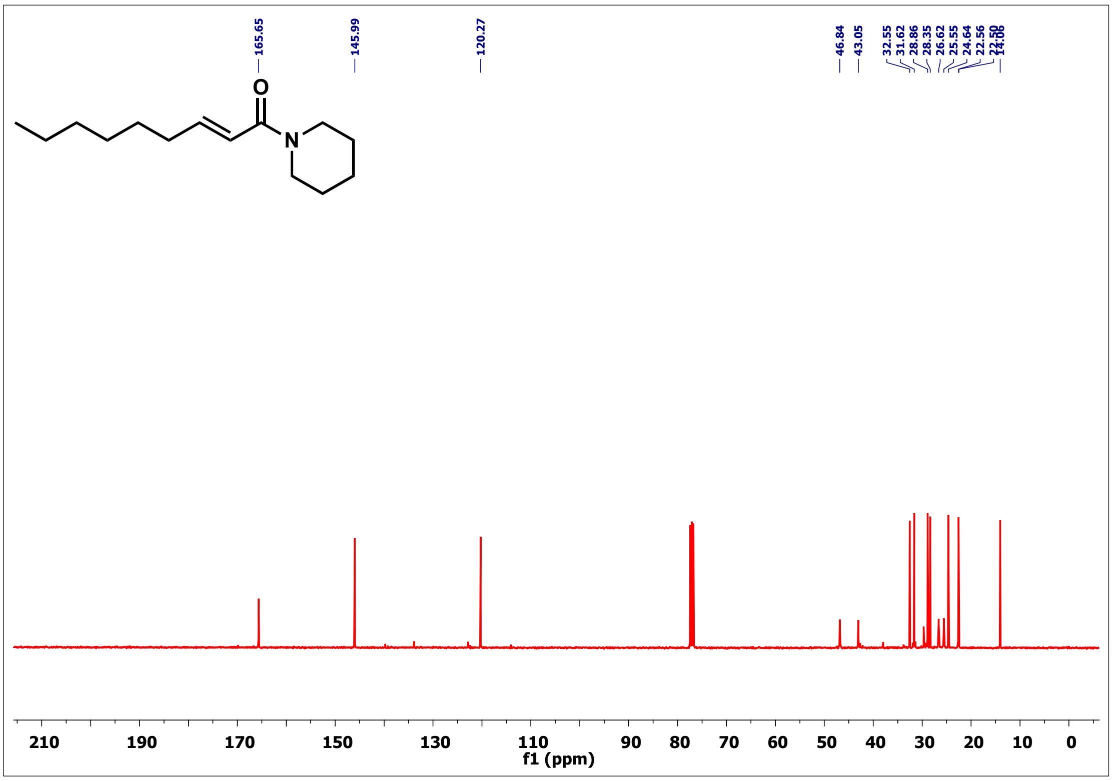

Figure 20: 100 MHz ^{13}C NMR spectrum of **CP-296** in $CDCl_3$

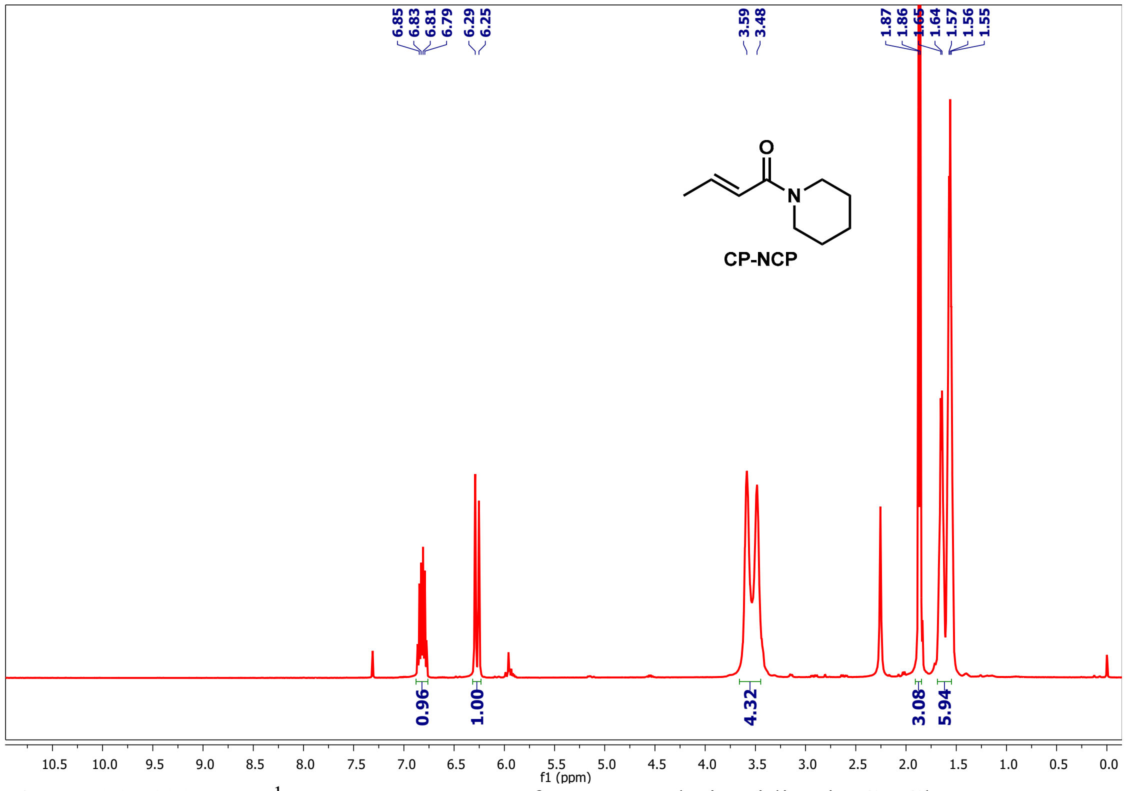

Figure 21: 400 MHz ^{1}H NMR spectrum of N-crotonyl piperidine in $CDCl_3$

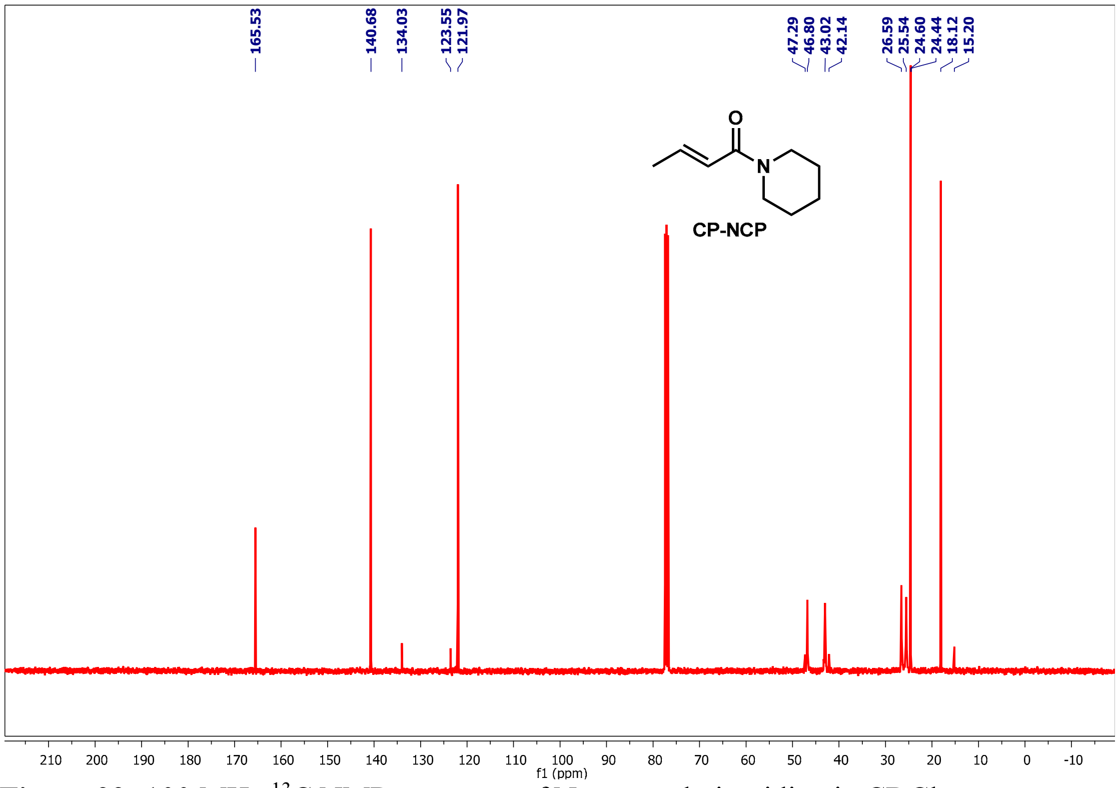

Figure 22: 100 MHz ^{13}C NMR spectrum of N-crotonyl piperidine in $CDCl_3$

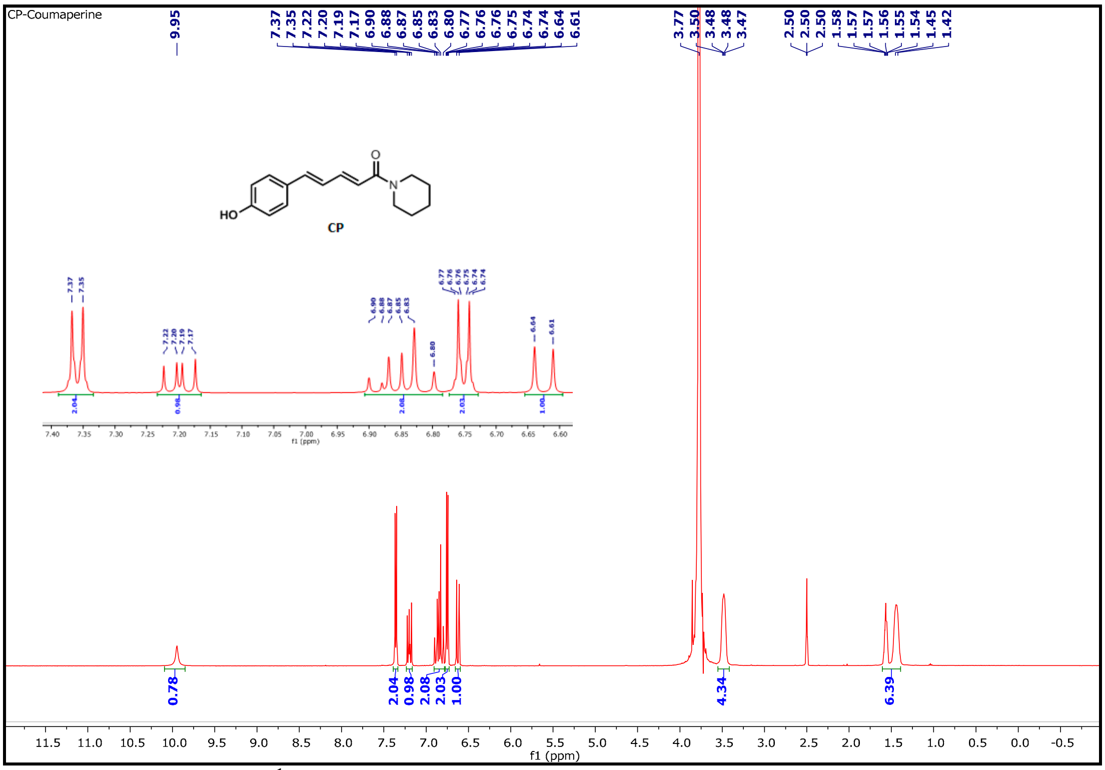

Figure 23: 500 MHz ^{1}H NMR spectrum of Coumaperine (**CP**) in DMSO-d_6

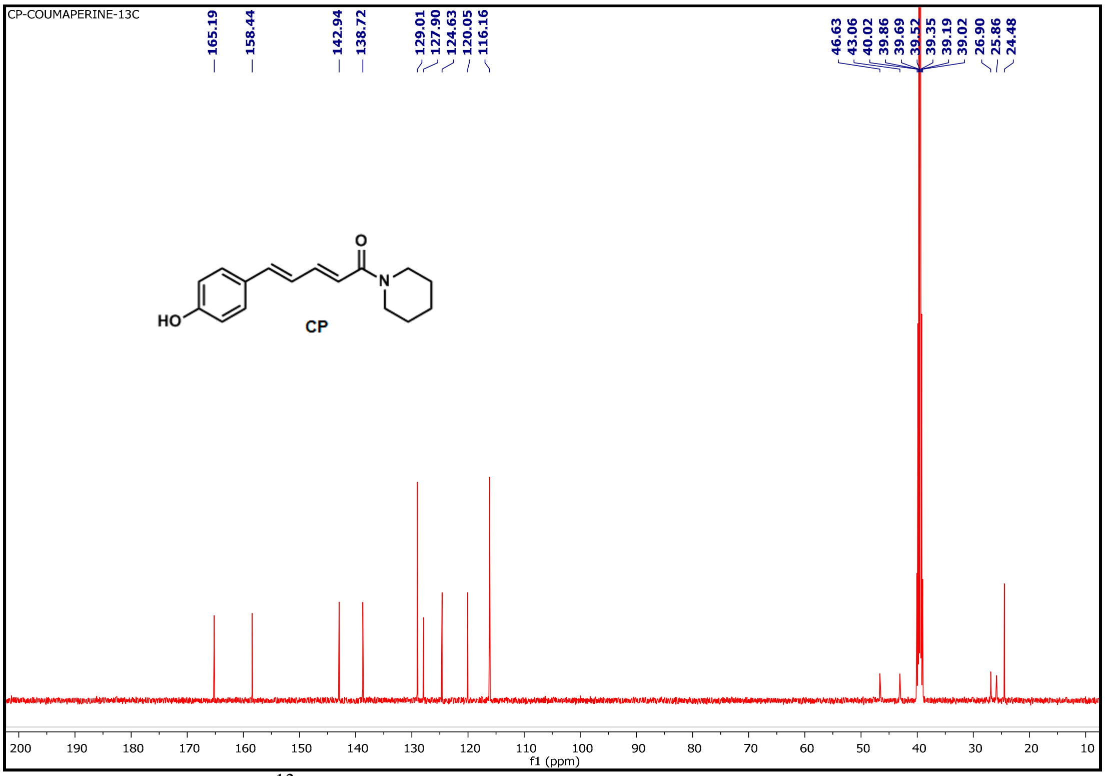

Figure 24: 125 MHz ^{13}C NMR spectrum of **Coumaperine** (**CP**) in DMSO-d_6

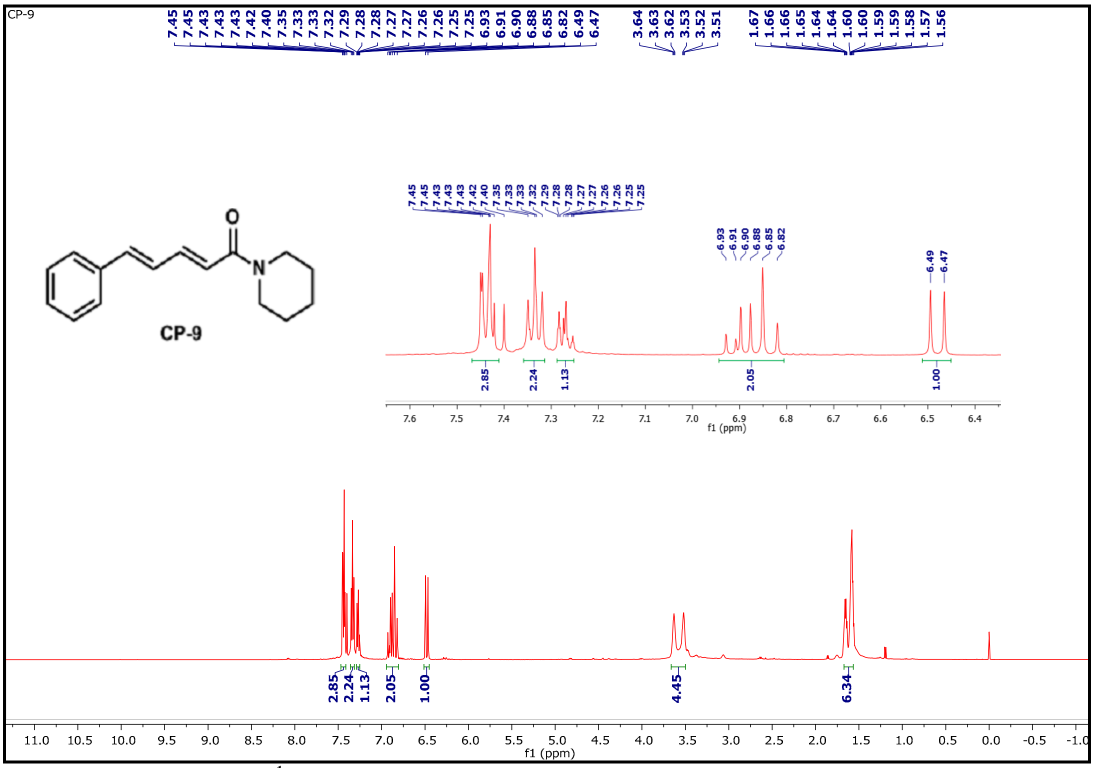

Figure 25: 500 MHz ^{1}H NMR spectrum of **CP-9** in $CDCl_3$

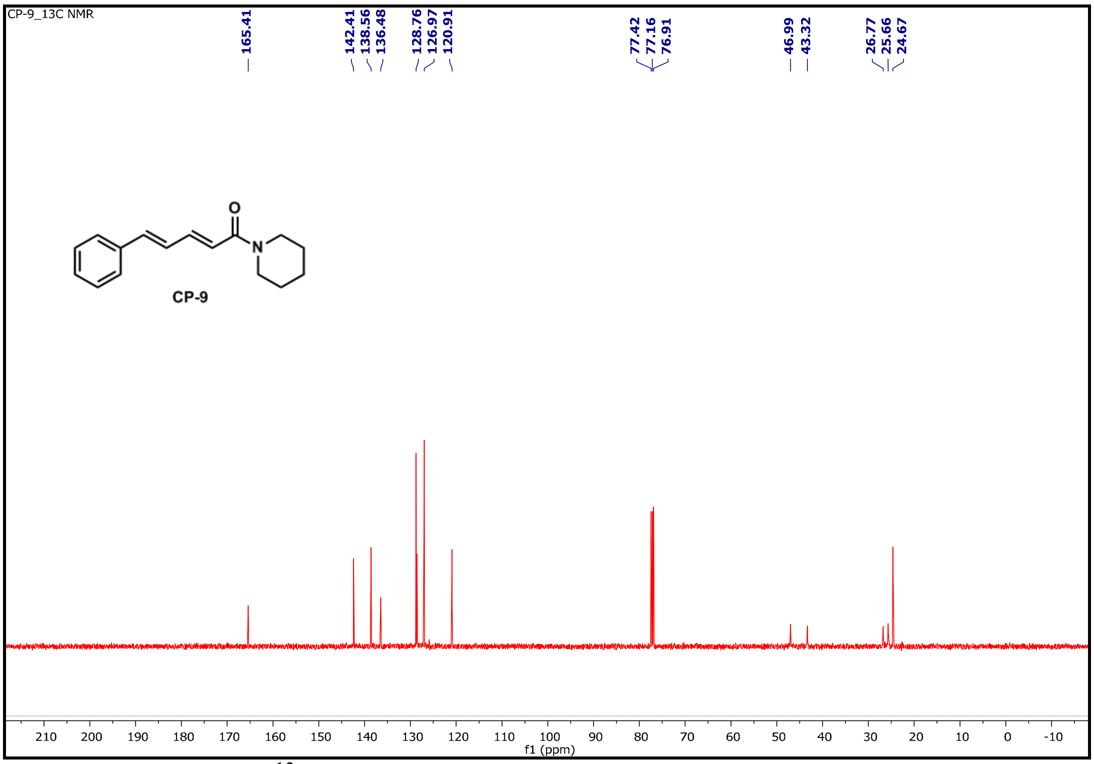

Figure 26: 125 MHz ^{13}C NMR spectrum of **CP-9** in $CDCl_3$

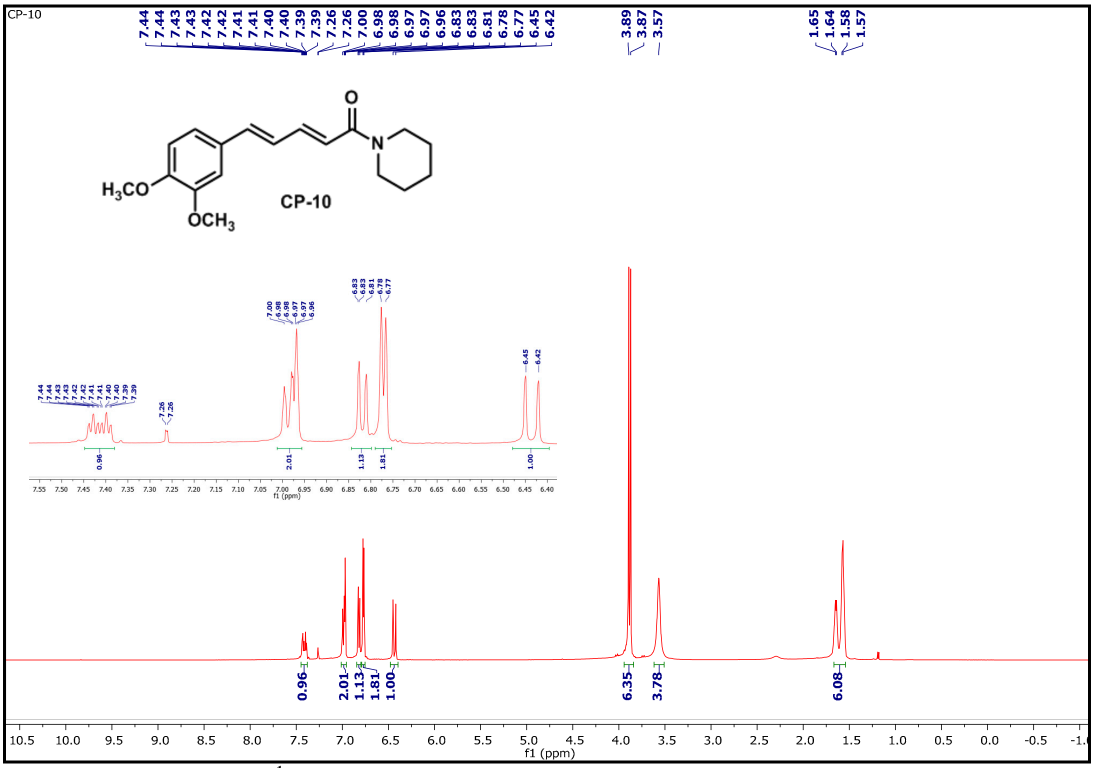

Figure 27: 500 MHz ^{1}H NMR spectrum of **CP-10** in $CDCl_3$

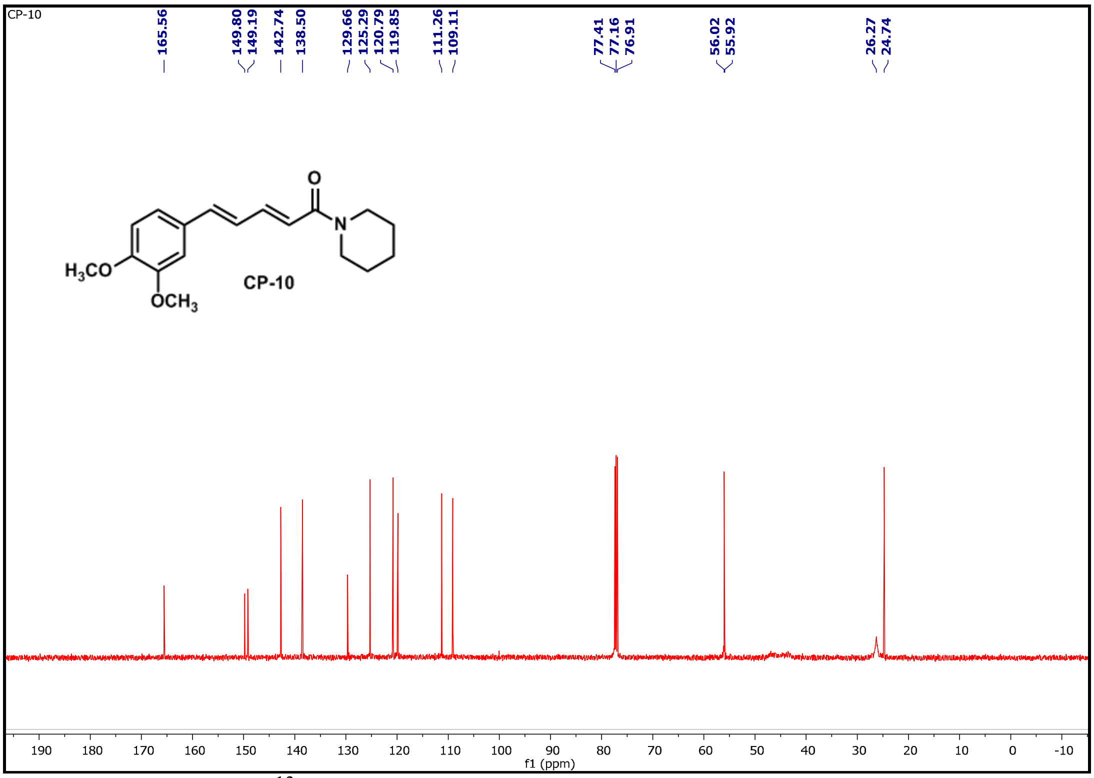

Figure 28: 125 MHz ^{13}C NMR spectrum of **CP-10** in $CDCl_3$

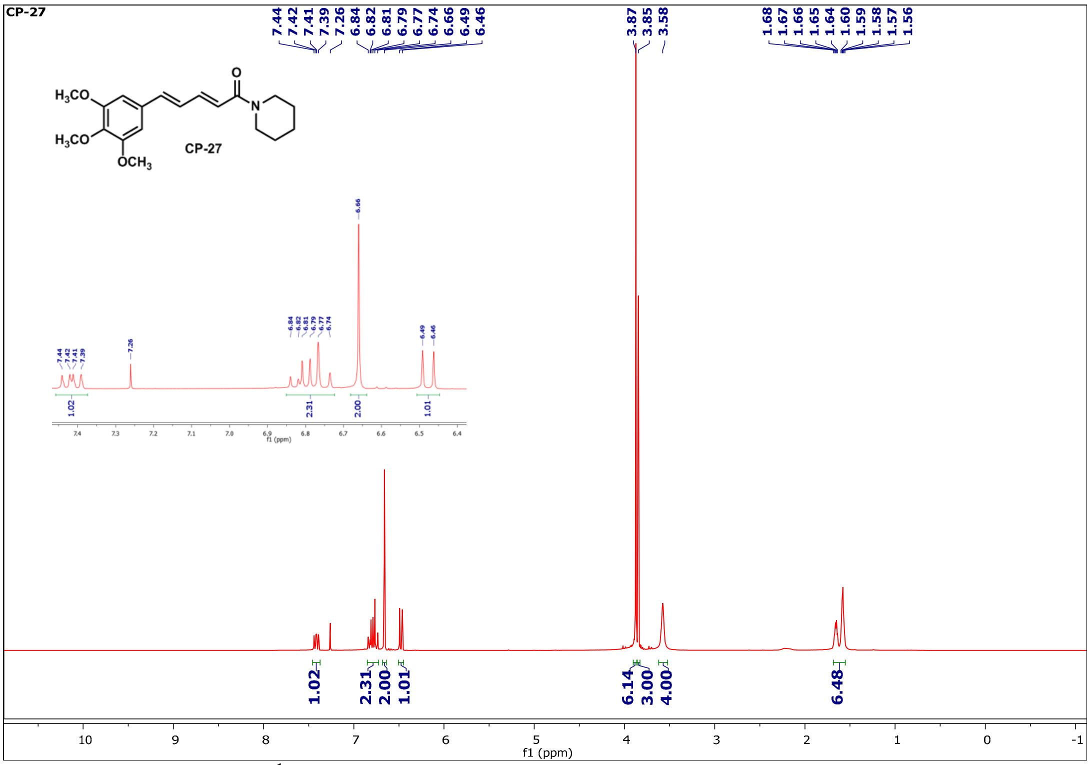

Figure 29: 500 MHz ^{1}H NMR spectrum of **CP-27** in $CDCl_3$

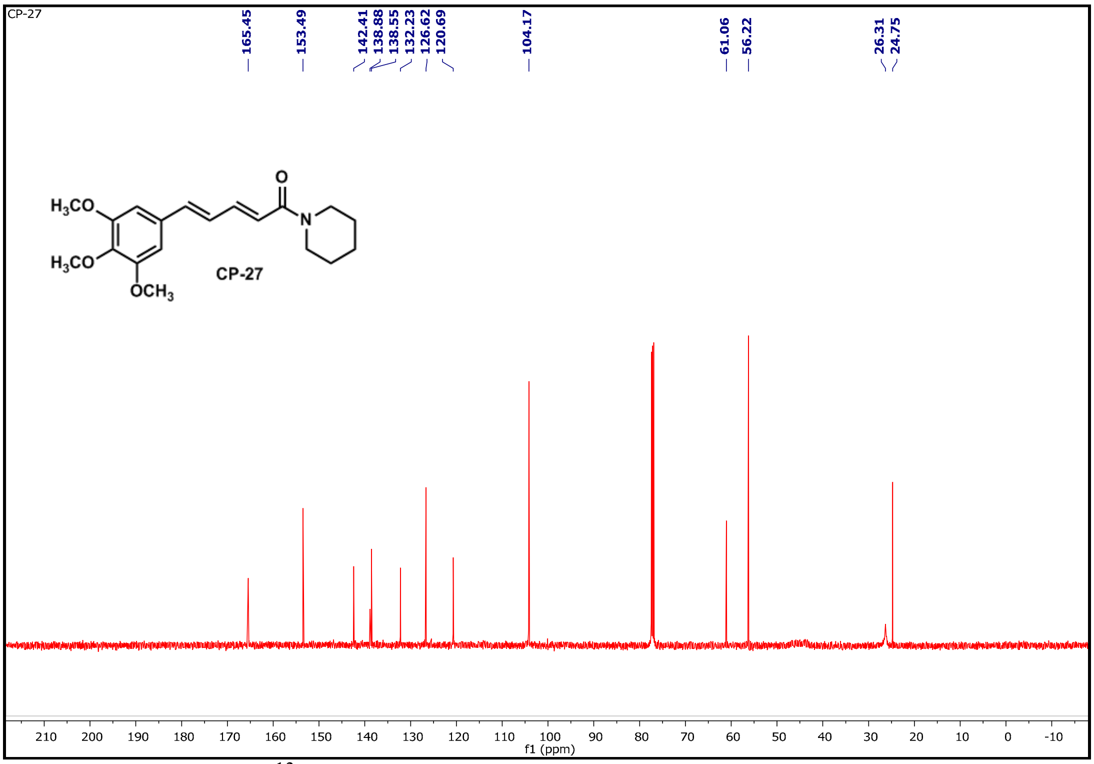

Figure 30: 125 MHz ^{13}C NMR spectrum of **CP-27** in $CDCl_3$

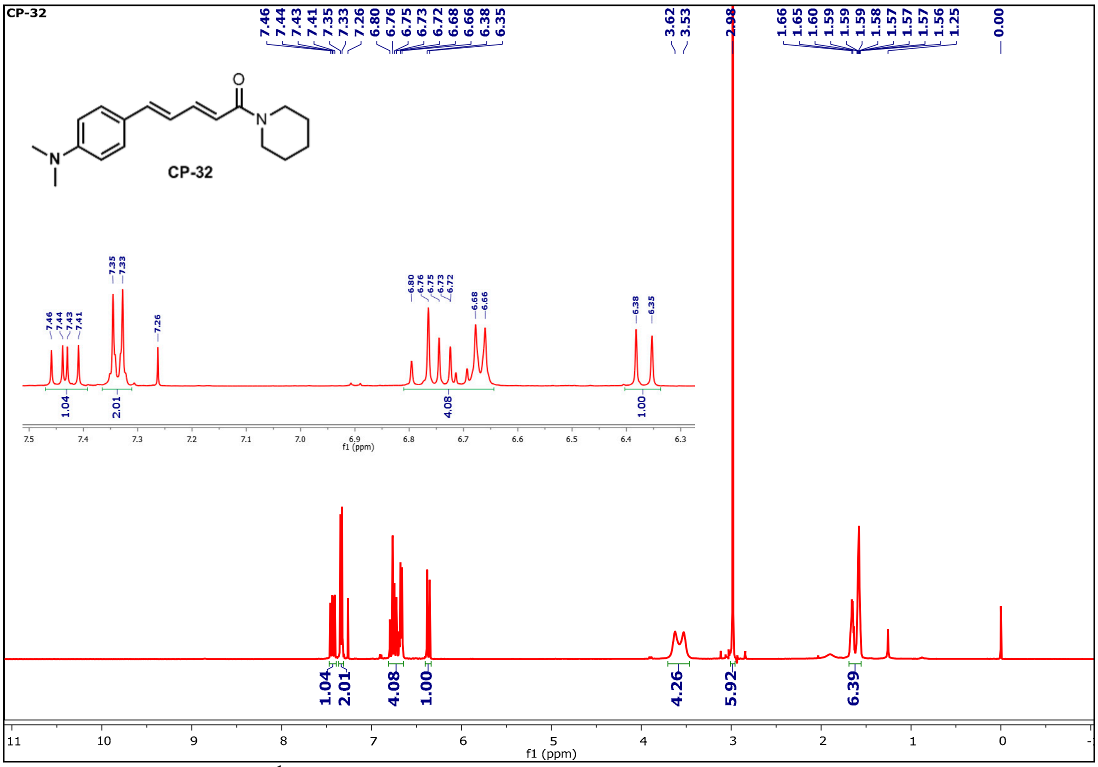

Figure 31: 500 MHz ^{1}H NMR spectrum of **CP-32** in $CDCl_3$

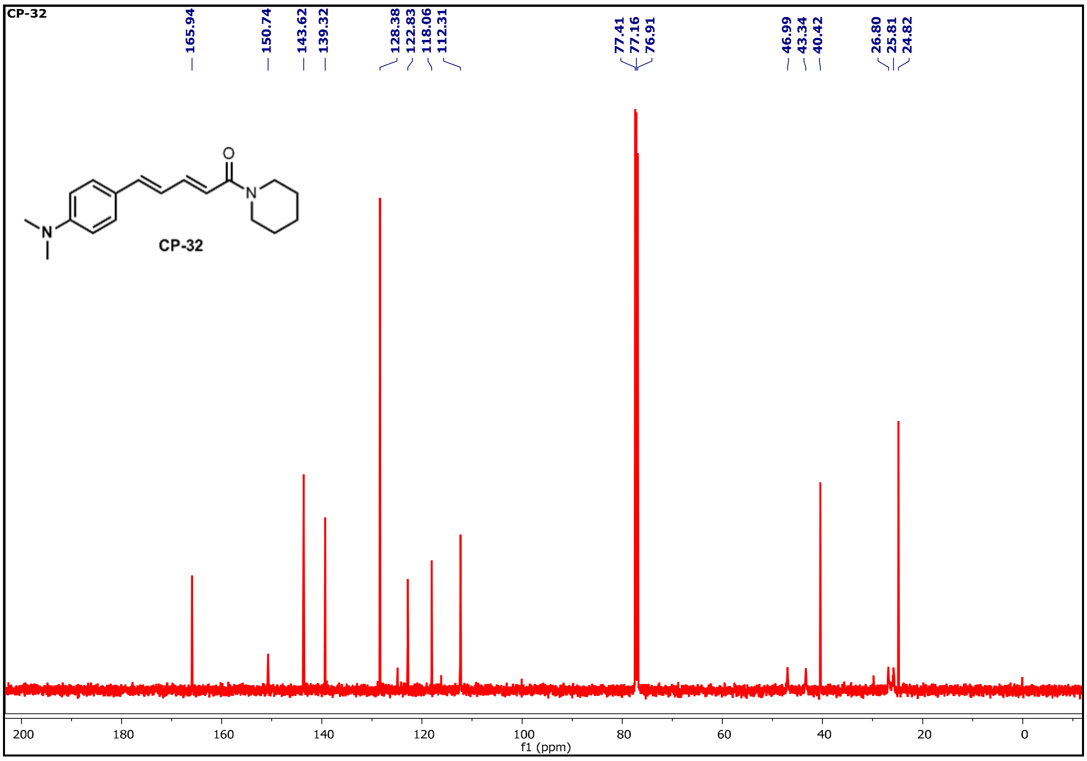

Figure 32: 125 MHz ^{13}C NMR spectrum of **CP-32** in $CDCl_3$

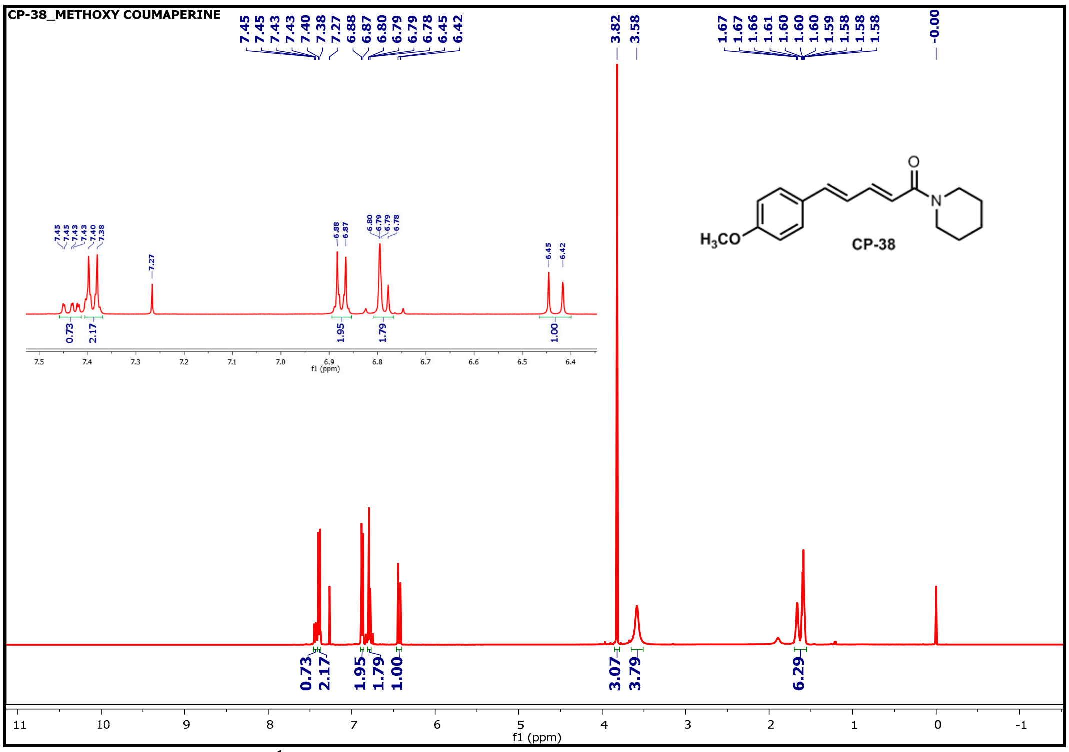

Figure 33: 500 MHz ^{1}H NMR spectrum of **CP-38** in $CDCl_3$

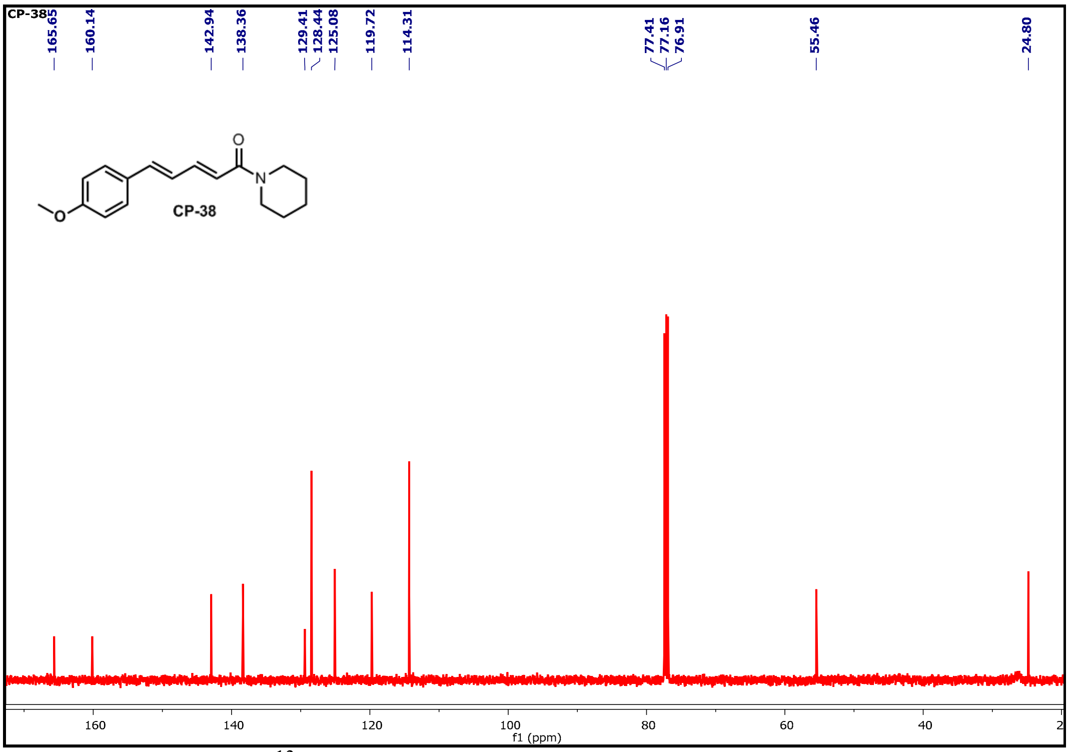

Figure 34: 125 MHz ^{13}C NMR spectrum of **CP-38** in $CDCl_3$

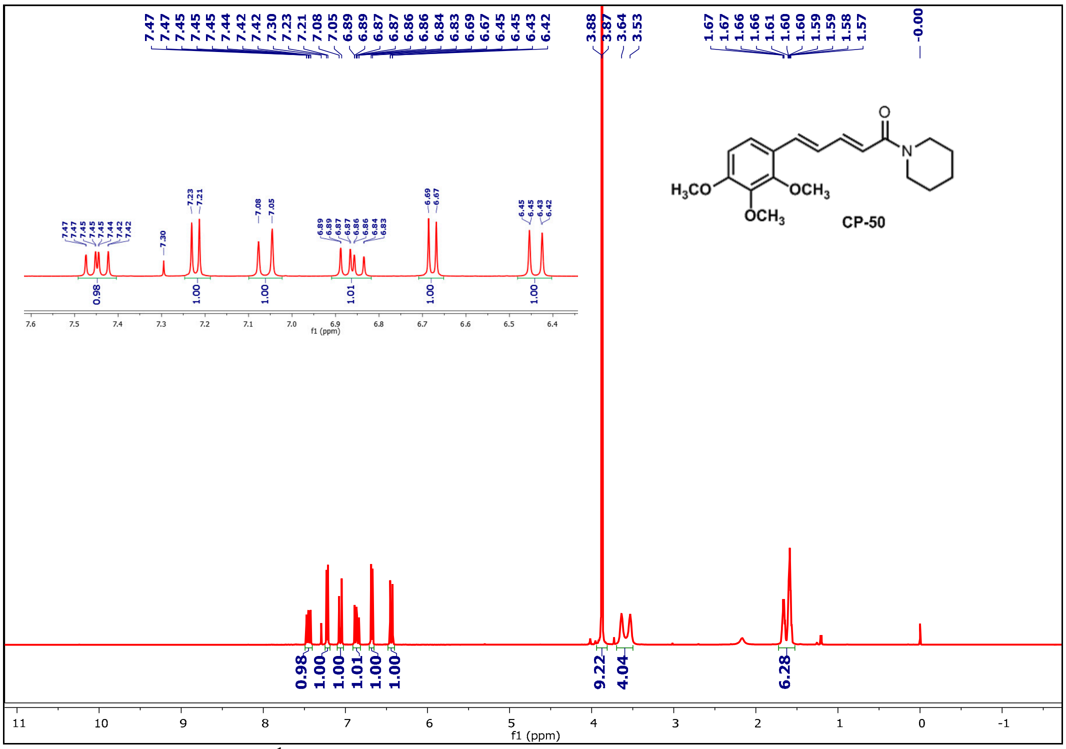

Figure 35: 500 MHz ^{1}H NMR spectrum of **CP-50** in $CDCl_3$

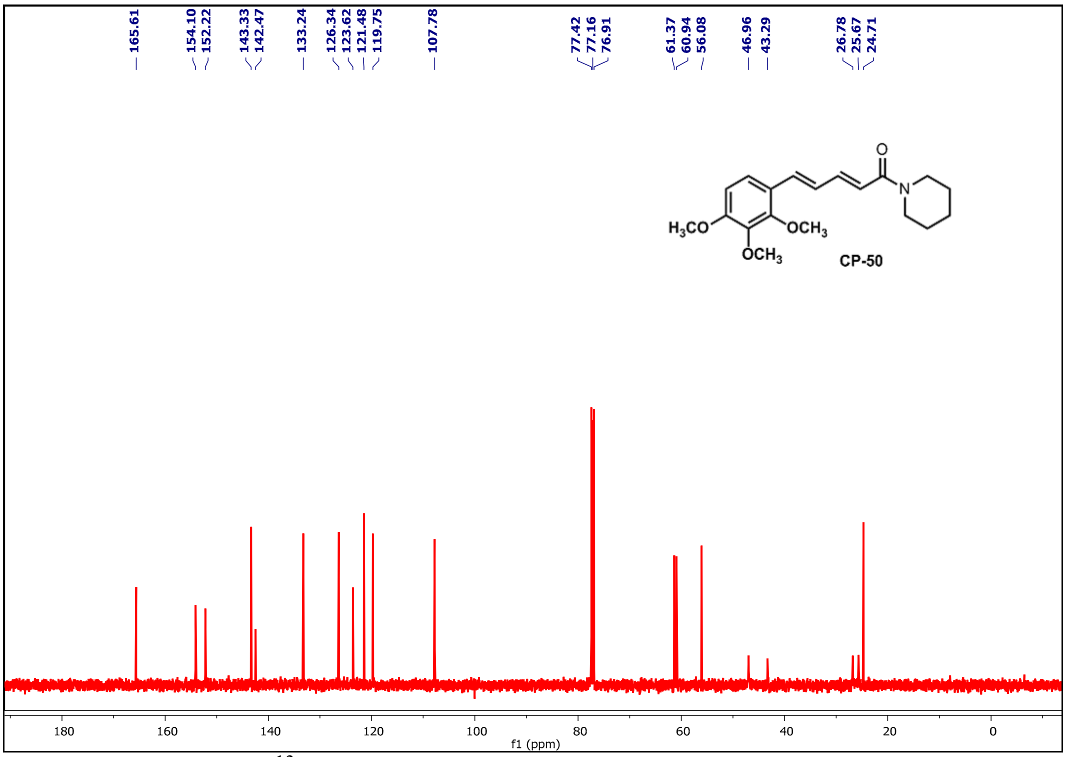

Figure 36: 125 MHz ^{13}C NMR spectrum of **CP-50** in $CDCl_3$

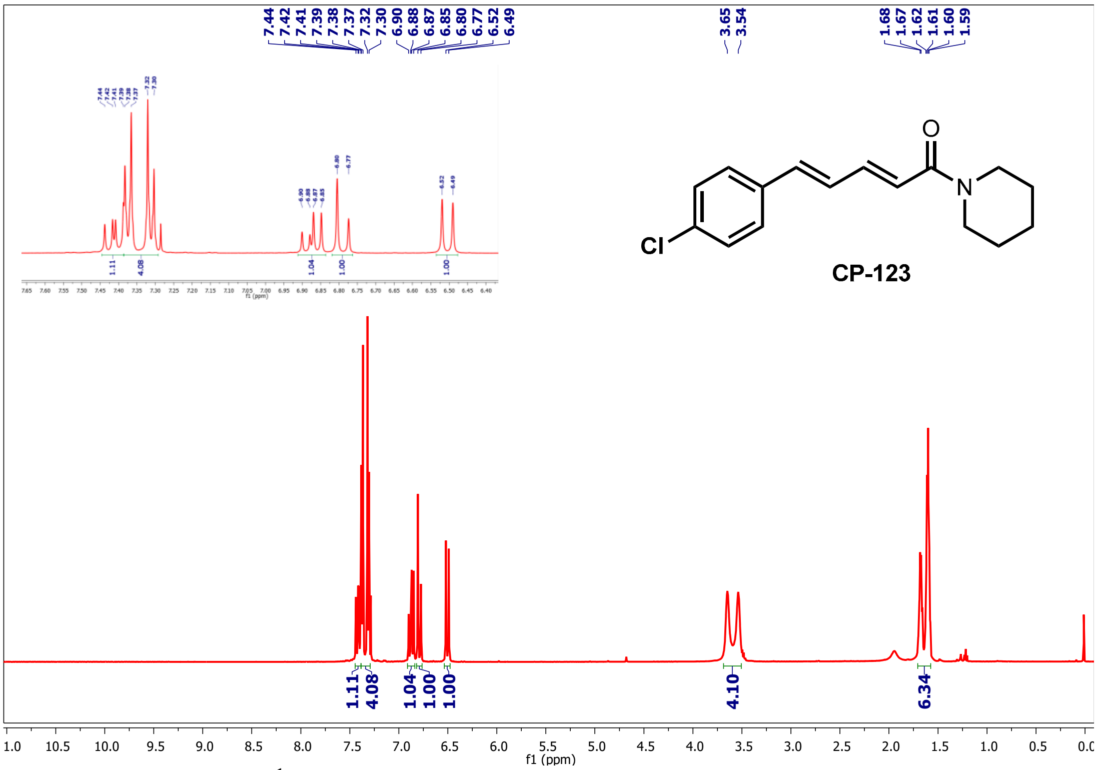

Figure 37: 500 MHz ^{1}H NMR spectrum of **CP-123** in $CDCl_3$

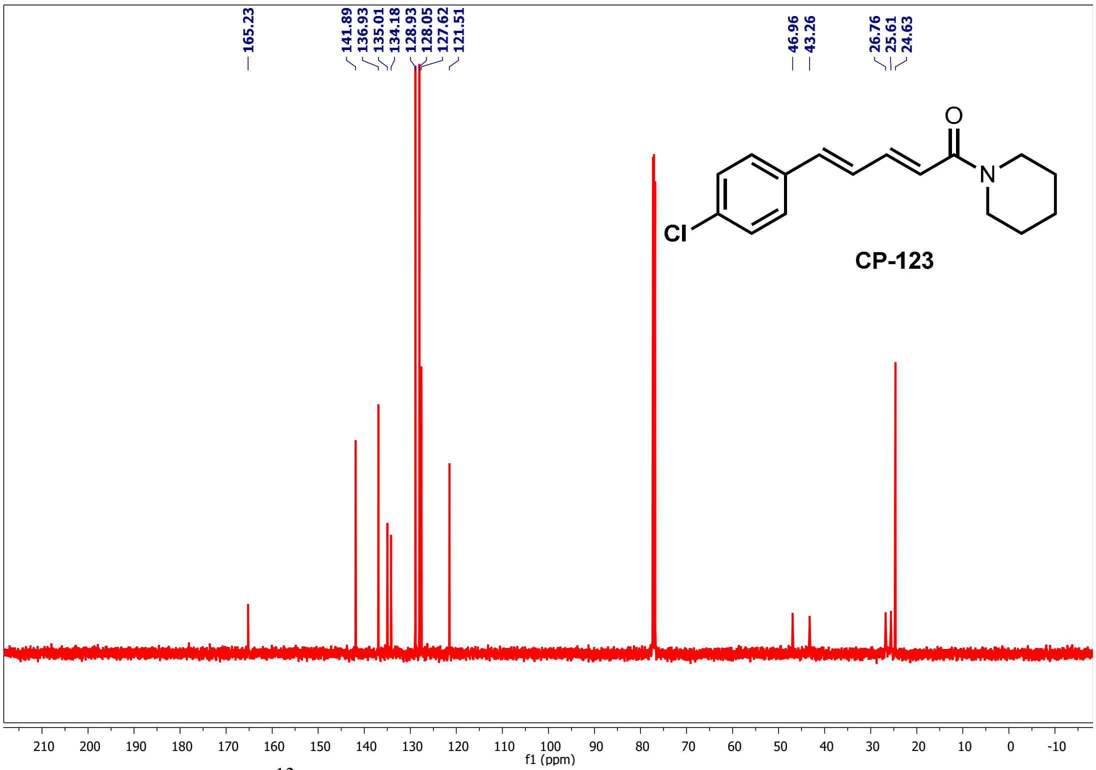

Figure 38: 120 MHz ^{13}C NMR spectrum of **CP-123** in $CDCl_3$

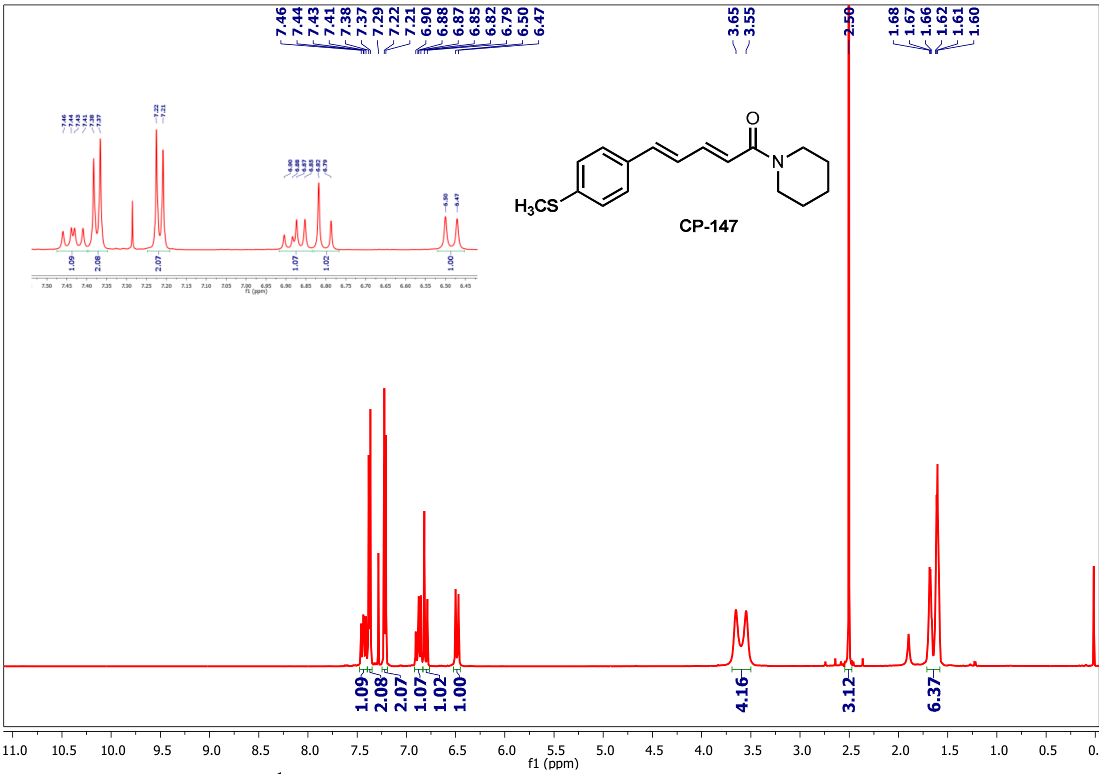

Figure 39: 500 MHz ^{1}H NMR spectrum of **CP-147** in $CDCl_3$

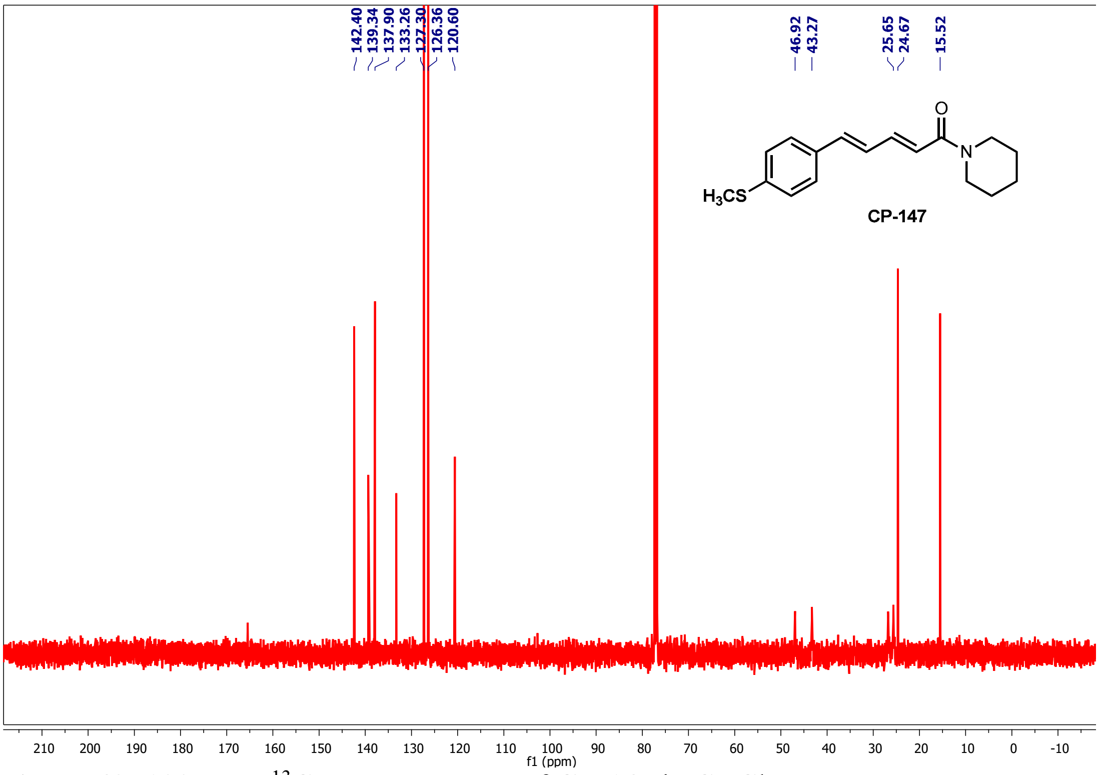

Figure 40: 100 MHz ^{13}C NMR spectrum of **CP-147** in $CDCl_3$

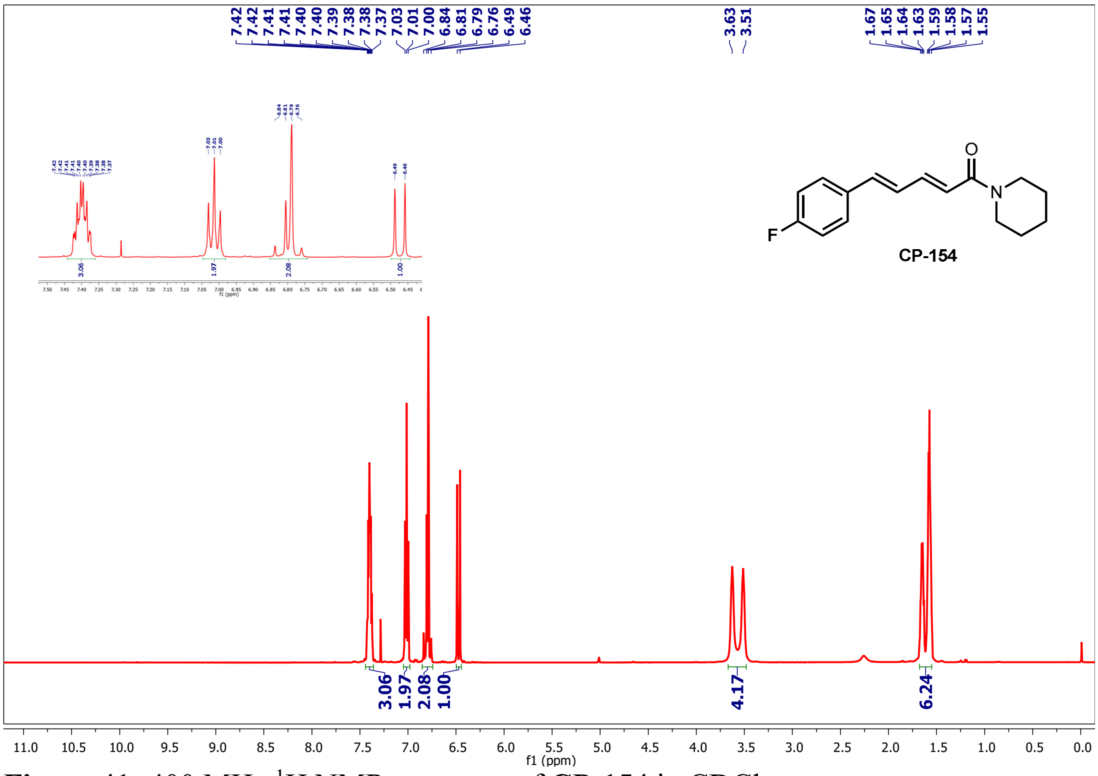

Figure 41: 400 MHz ^{1}H NMR spectrum of **CP-154** in $CDCl_3$

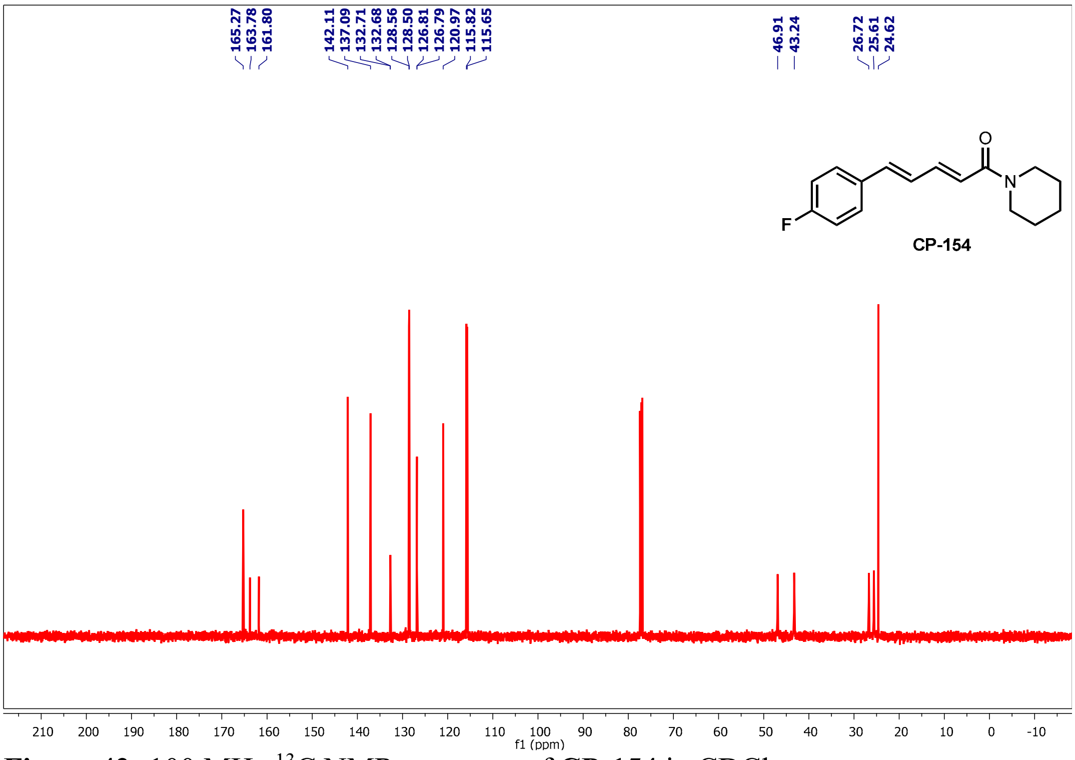

Figure 42: 100 MHz ^{13}C NMR spectrum of **CP-154** in $CDCl_3$

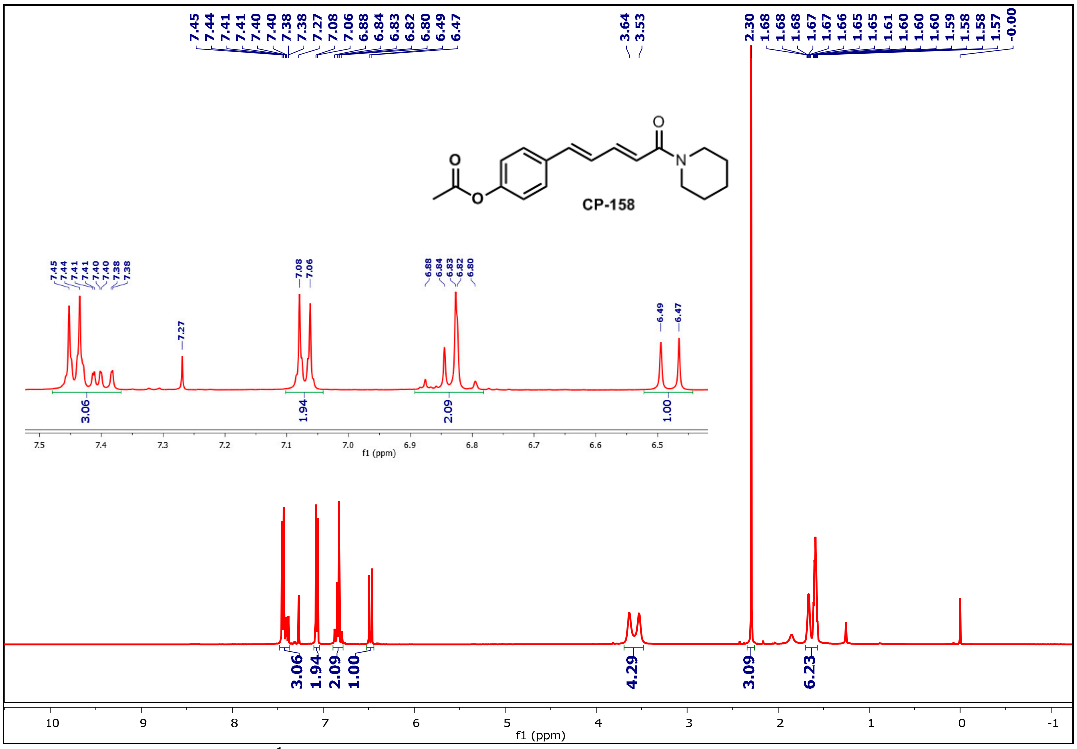

Figure 43: 500 MHz ^{1}H NMR spectrum of **CP-158** in $CDCl_3$

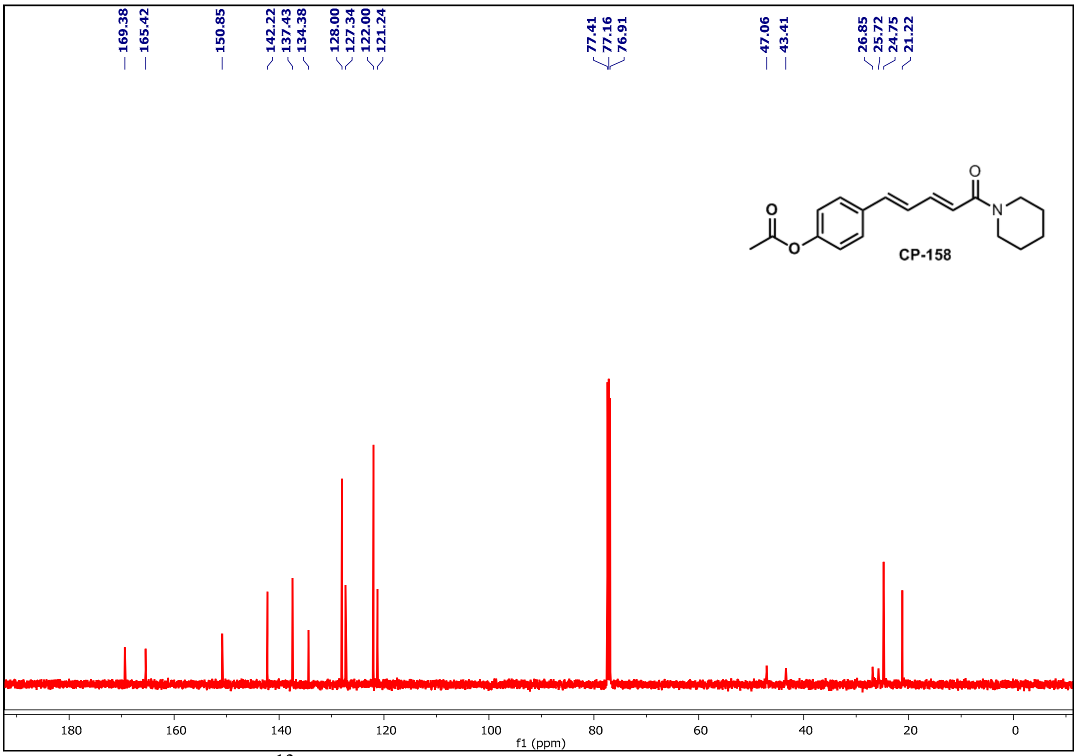

Figure 44: 125 MHz ^{13}C NMR spectrum of **CP-158** in $CDCl_3$

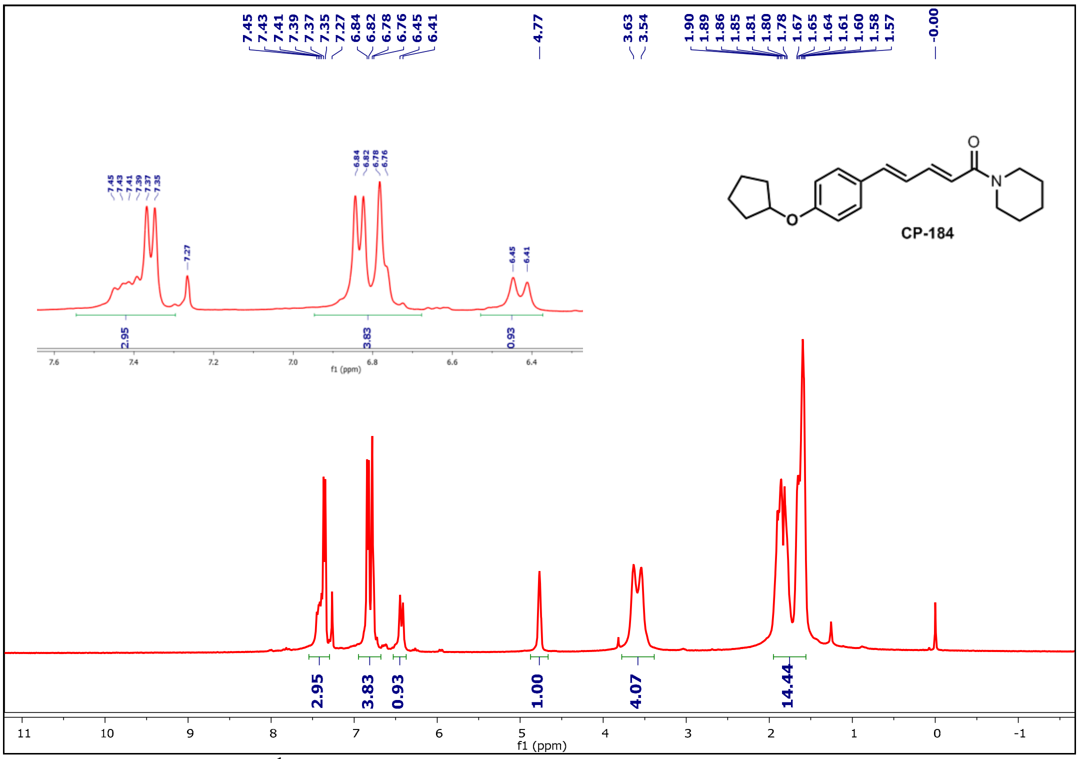

Figure 45: 400 MHz ^{1}H NMR spectrum of **CP-184** in $CDCl_3$

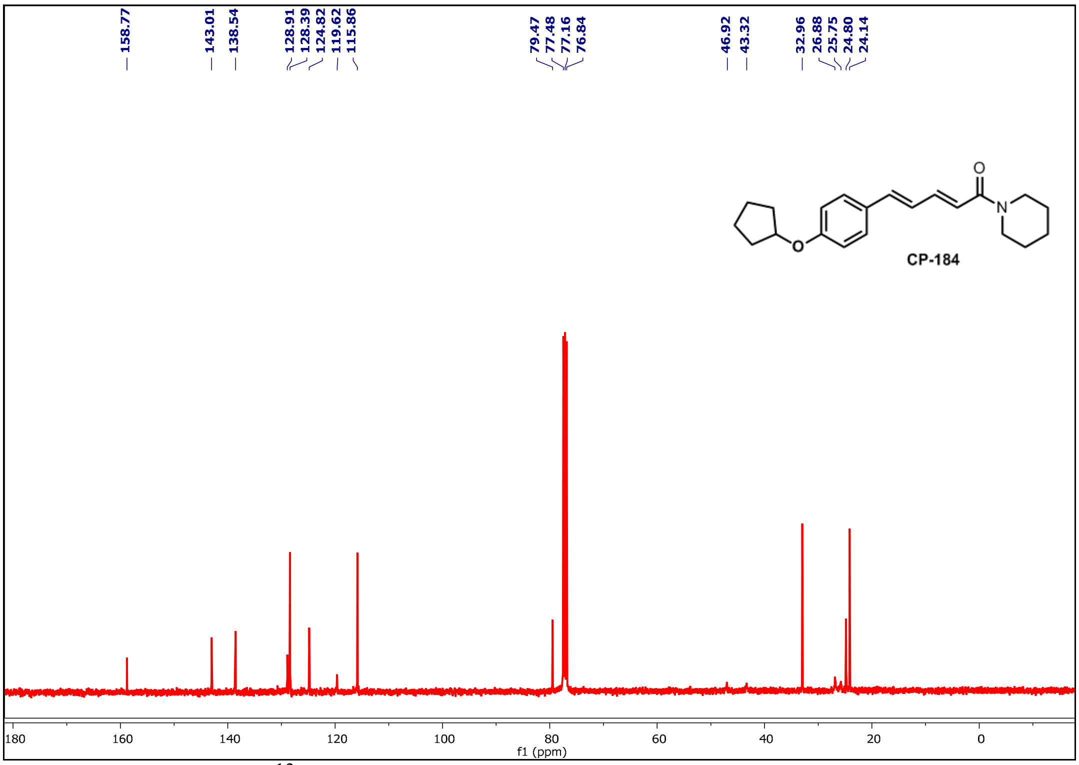

Figure 46: 100 MHz ^{13}C NMR spectrum of **CP-184** in $CDCl_3$

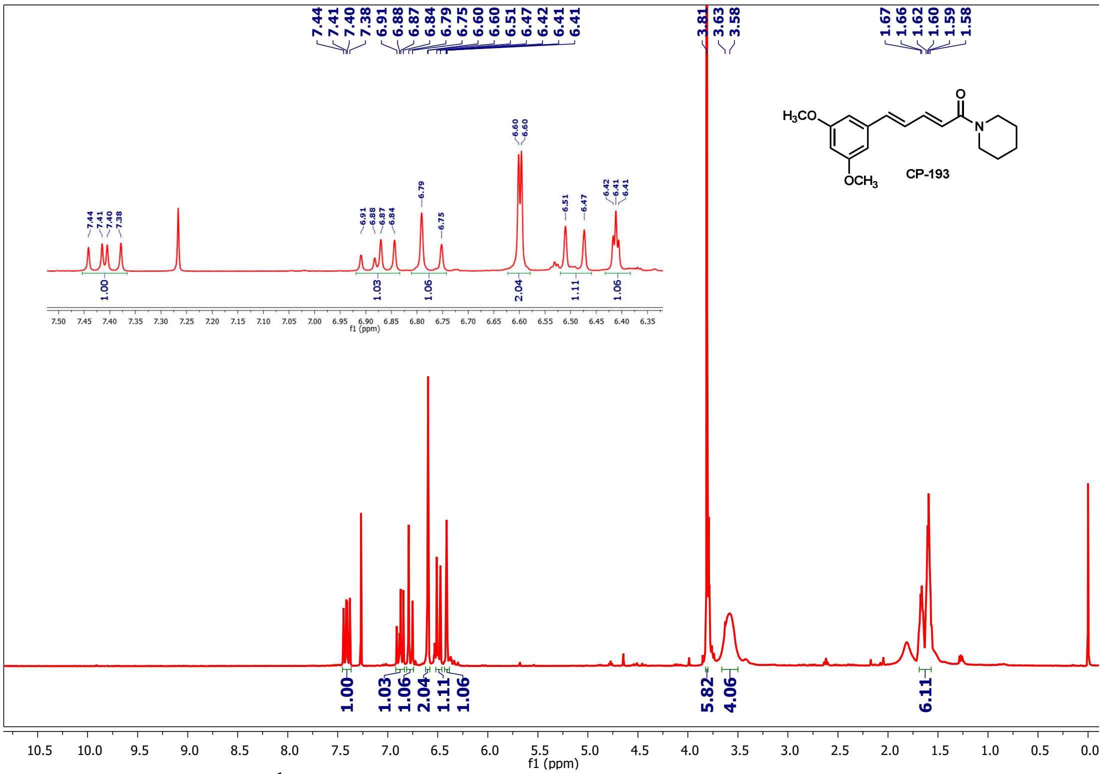

Figure 47: 400 MHz ^{1}H NMR spectrum of **CP-193** in $CDCl_3$

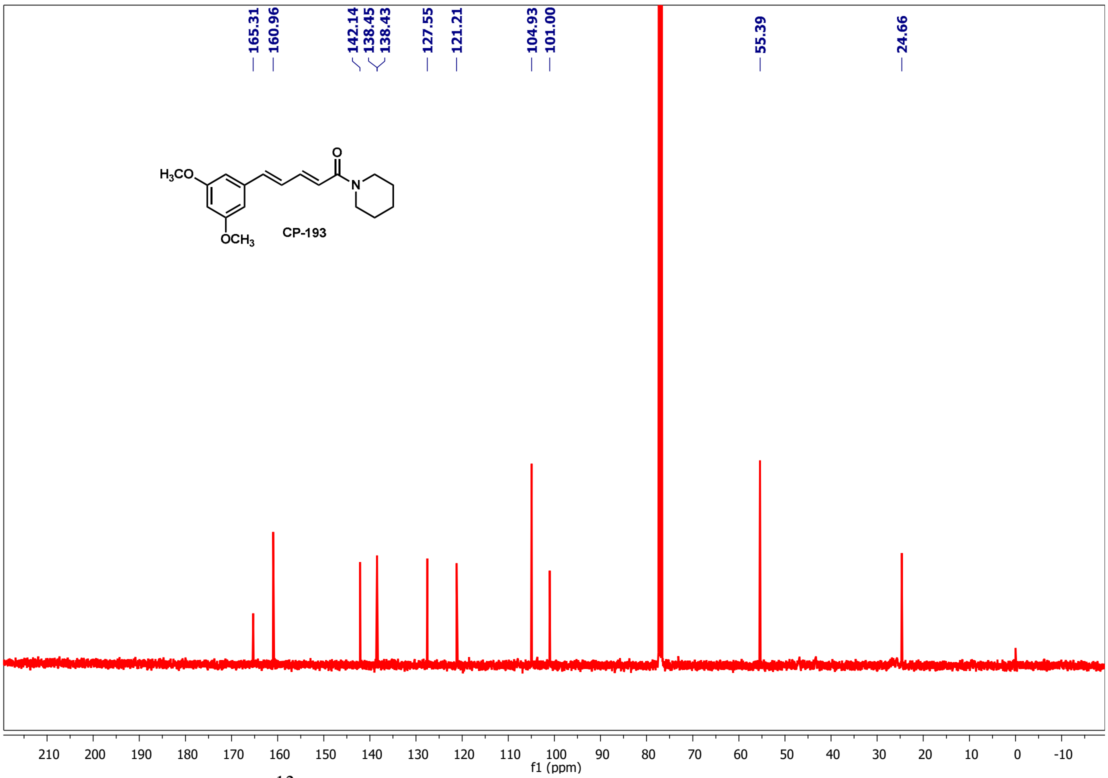

Figure 48: 100 MHz ^{13}C NMR spectrum of **CP-193** in $CDCl_3$

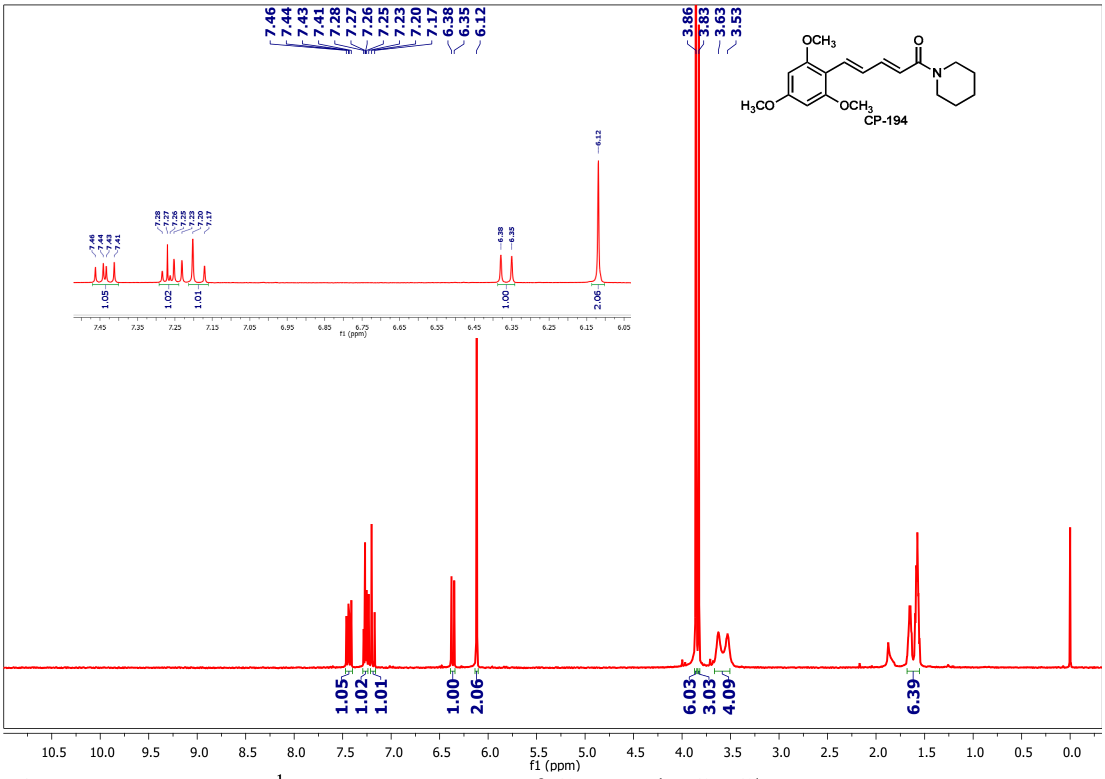

Figure 49: 500 MHz ^{1}H NMR spectrum of **CP-194** in $CDCl_3$

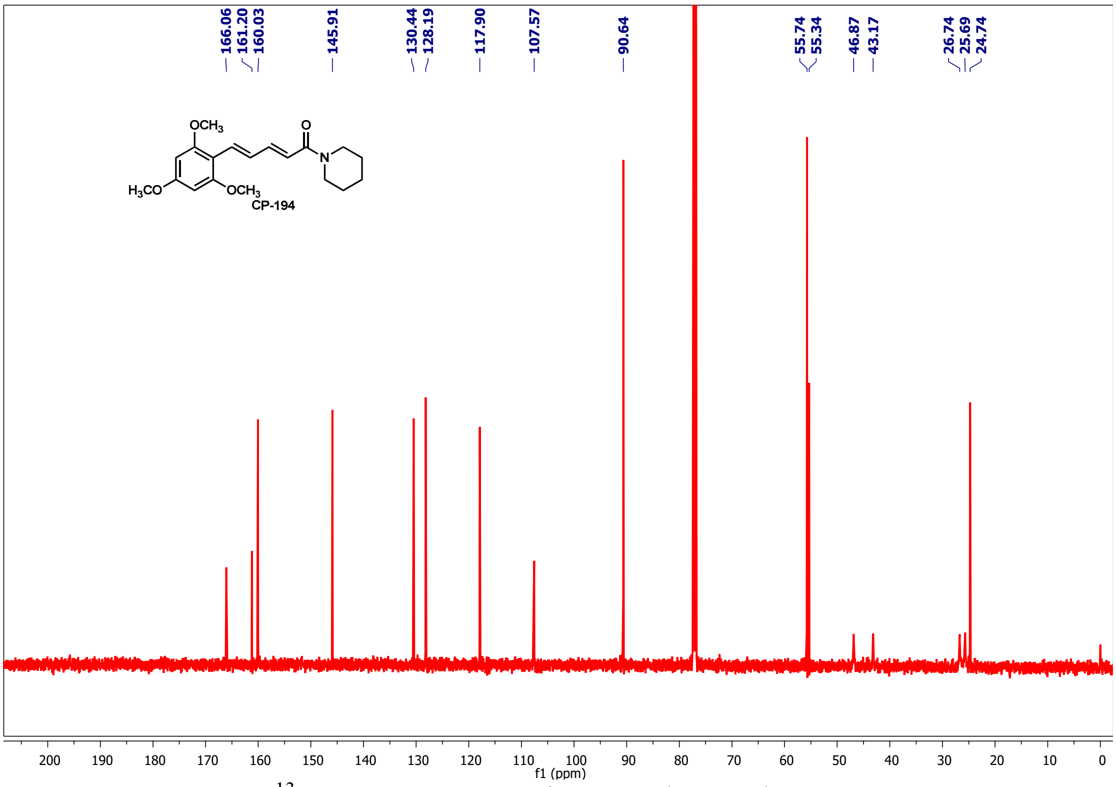

Figure 50: 125 MHz ^{13}C NMR spectrum of **CP-194** in $CDCl_3$

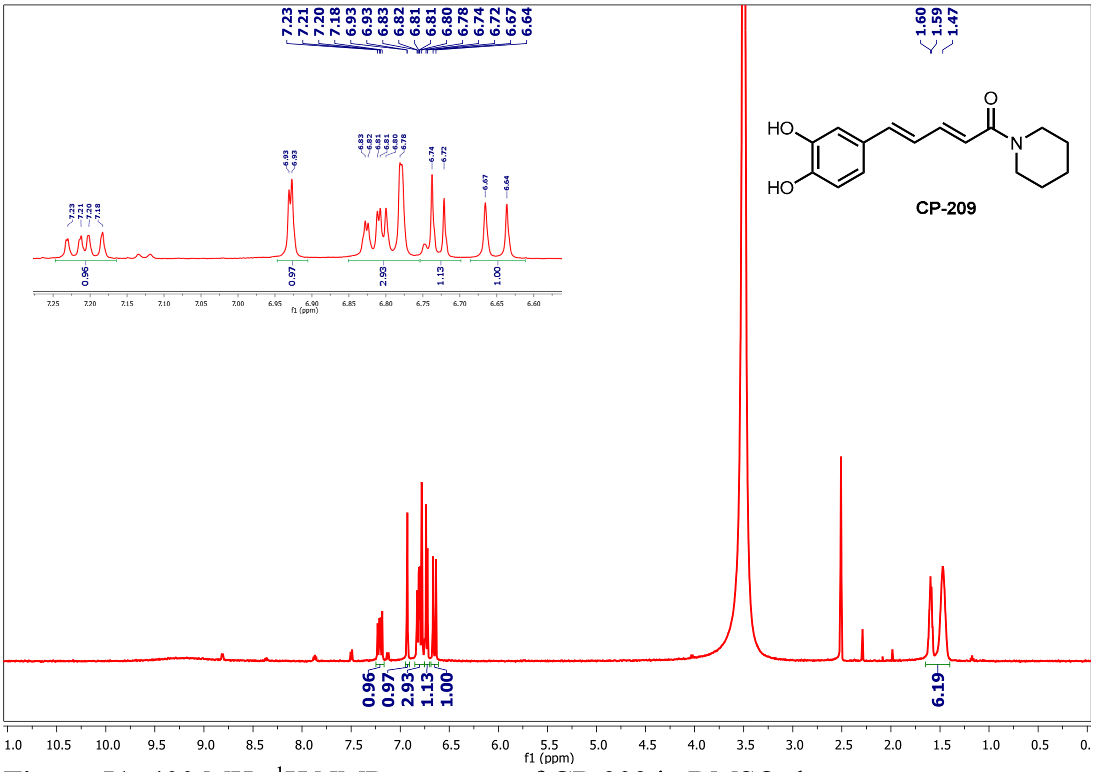

Figure 51: 400 MHz ^{1}H NMR spectrum of **CP-209** in DMSO-d_6

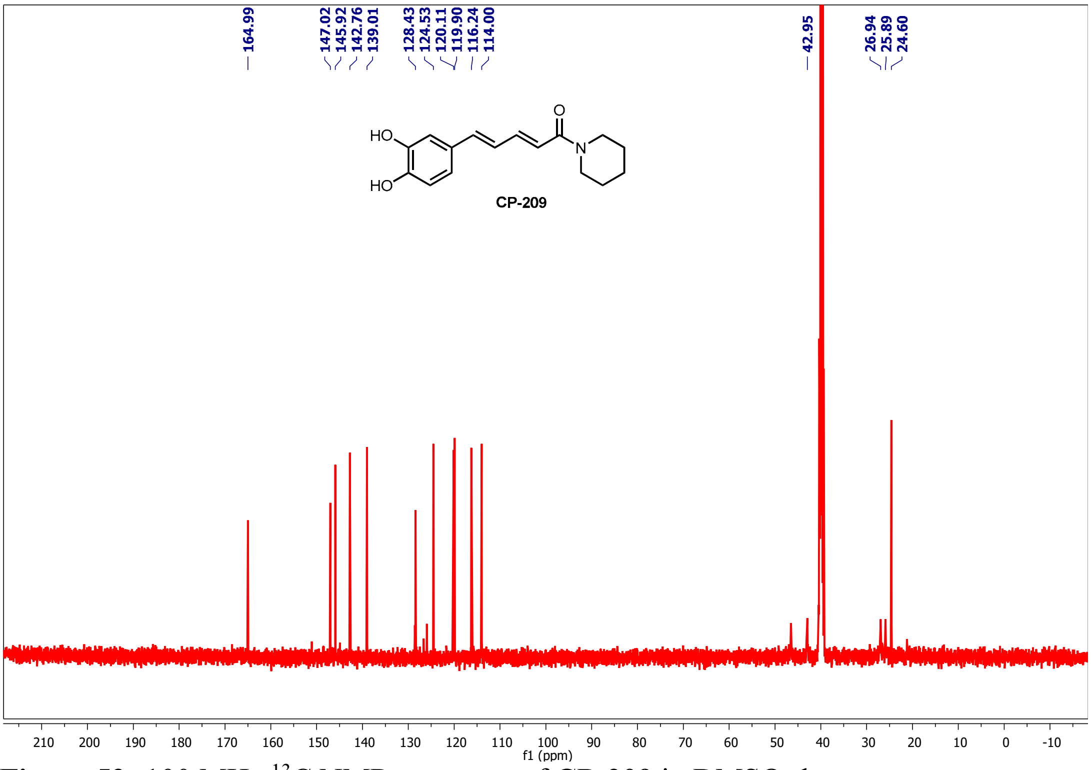

Figure 52: 100 MHz ^{13}C NMR spectrum of **CP-209** in DMSO-d_6

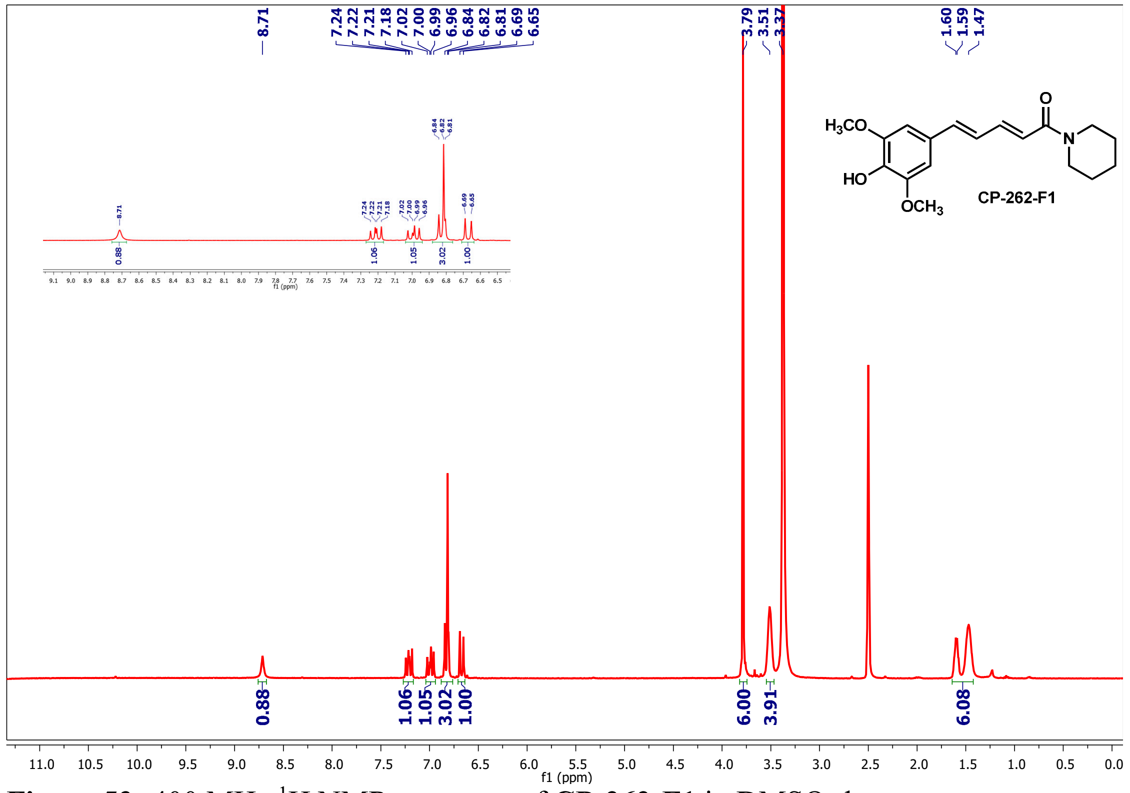

Figure 53: 400 MHz ^{1}H NMR spectrum of **CP-262-F1** in DMSO-d6

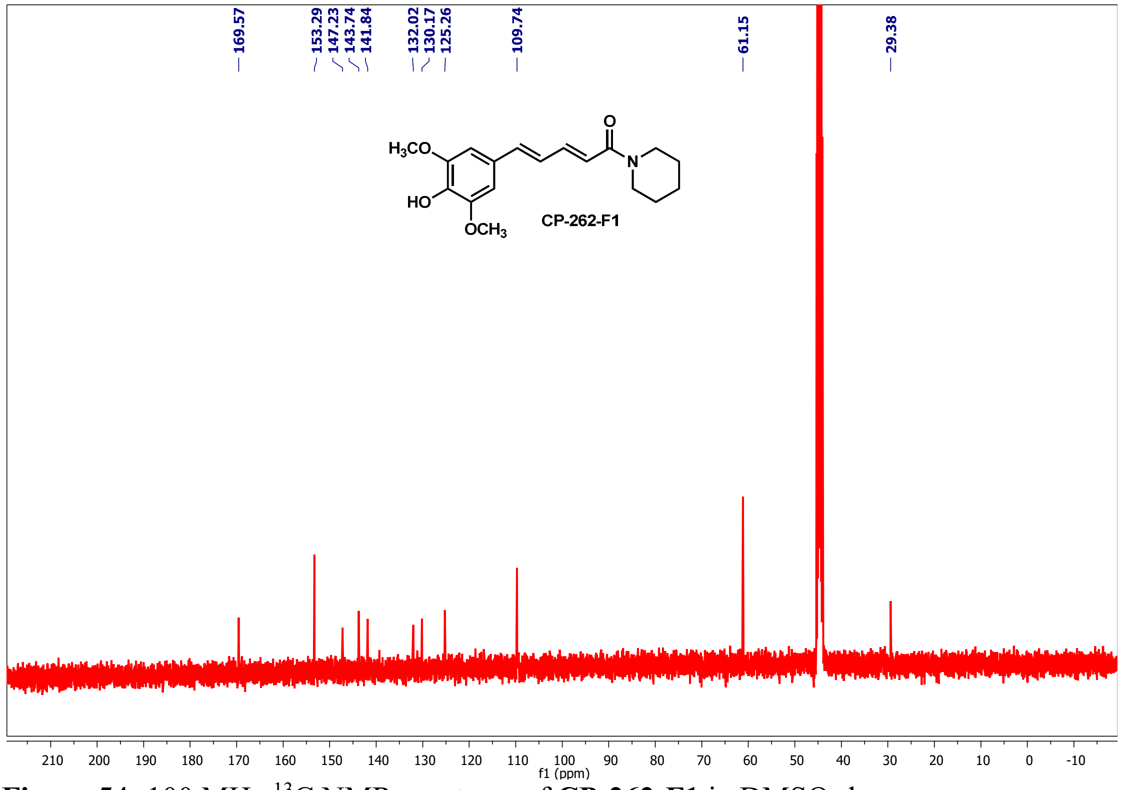

Figure 54: 100 MHz ^{13}C NMR spectrum of **CP-262-F1** in DMSO-d6

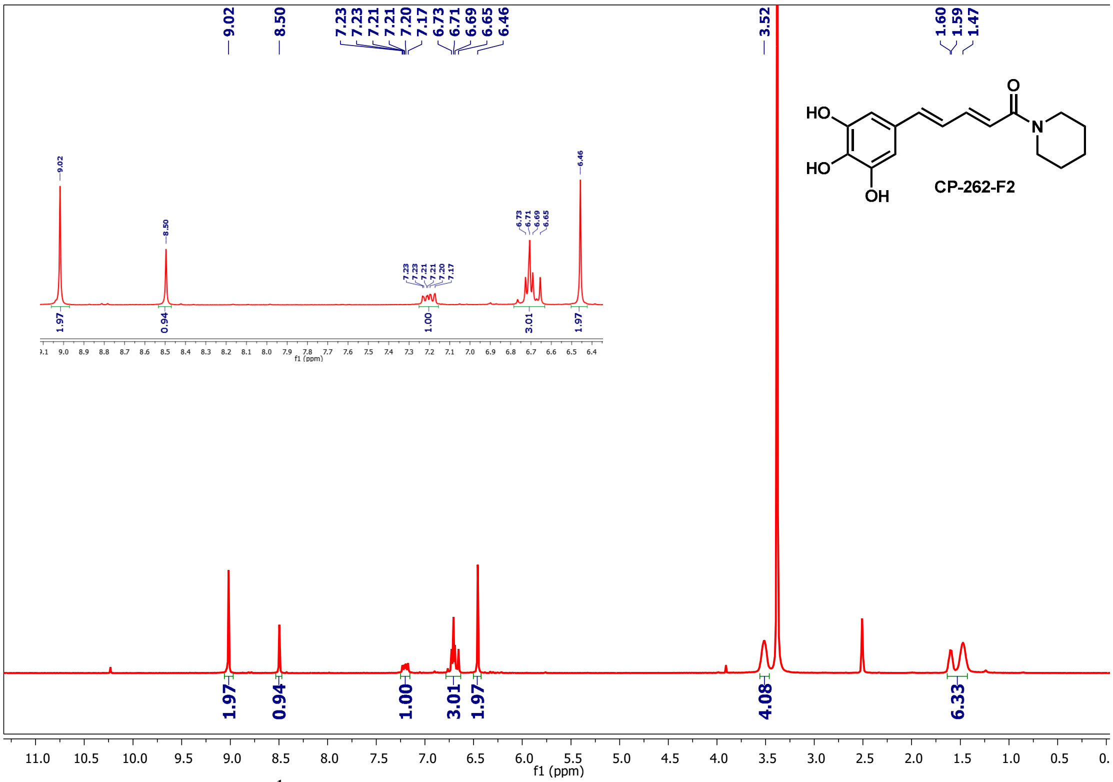

Figure 55: 400 MHz ^{1}H NMR spectrum of **CP-262-F2** in DMSO-d_6

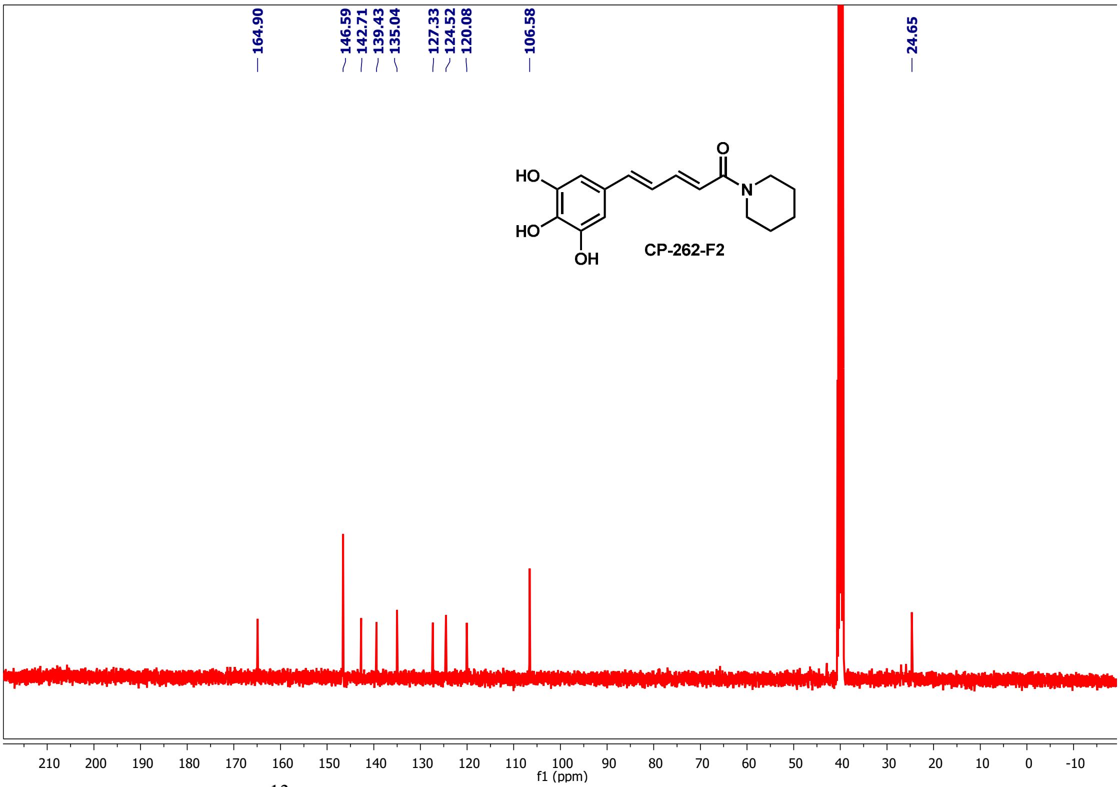

Figure 56: 100 MHz ^{13}C NMR spectrum of **CP-262-F2** in DMSO-d_6

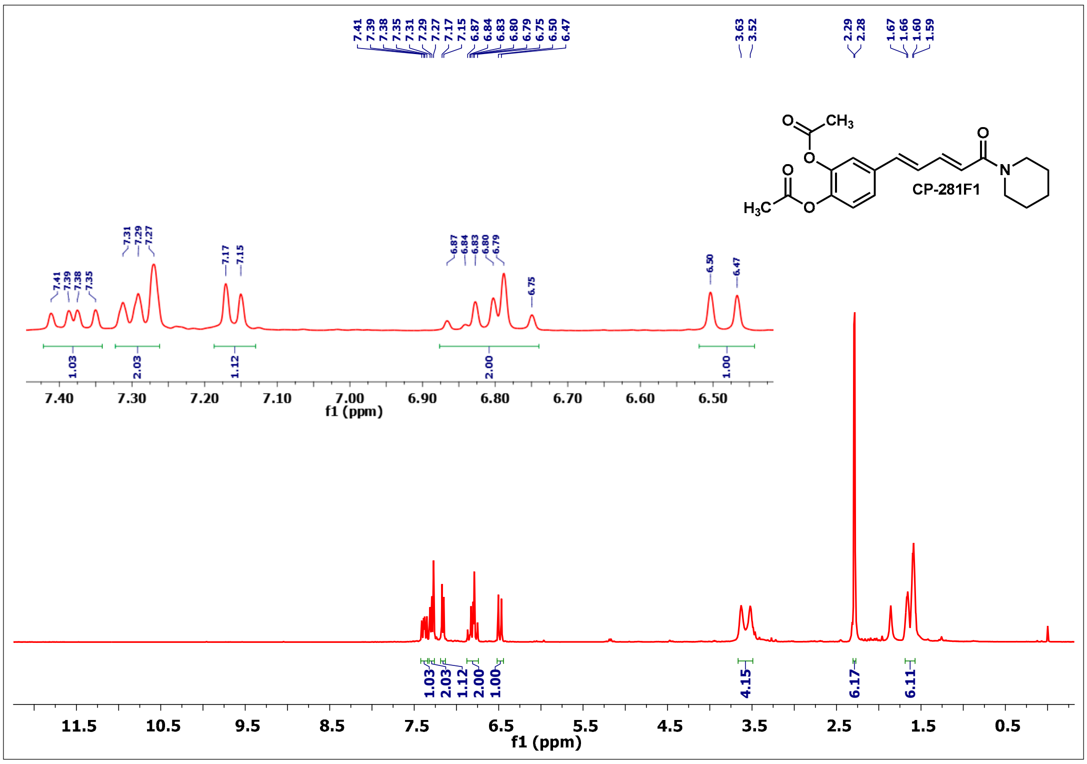

Figure 57: 400 MHz ^{1}H NMR spectrum of **CP-281-F1** in $CDCl_3$

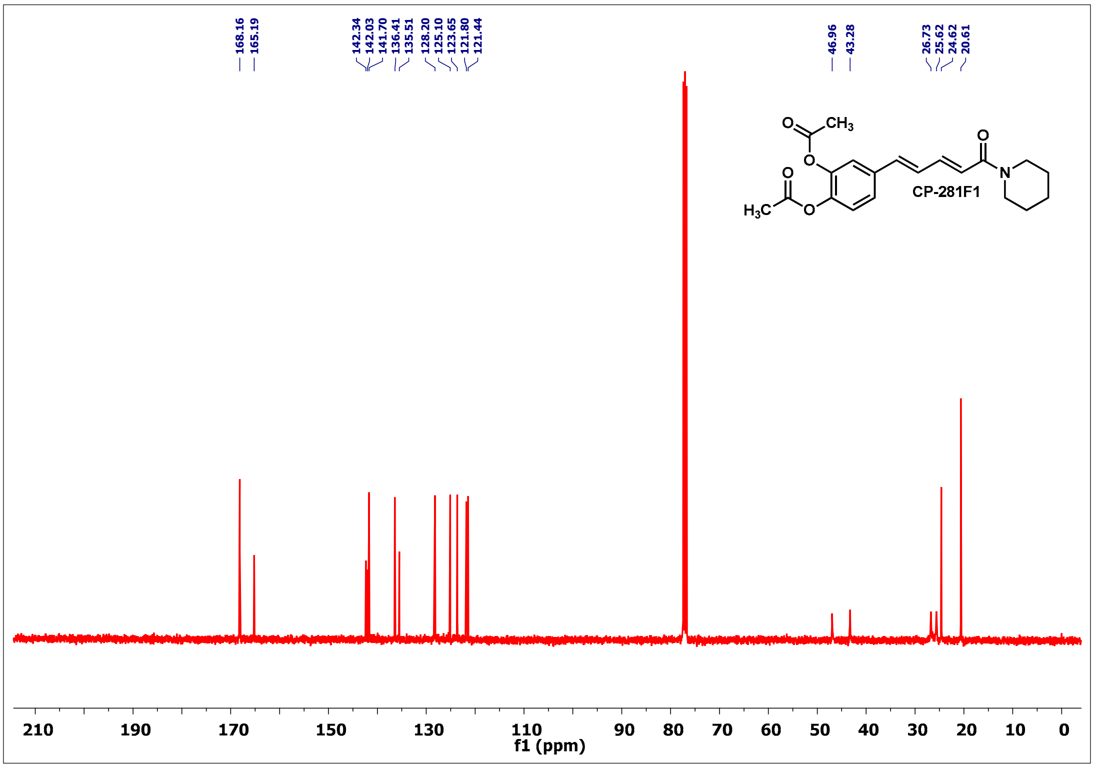

Figure 58: 100 MHz ^{13}C NMR spectrum of **CP-281-F1** in $CDCl_3$

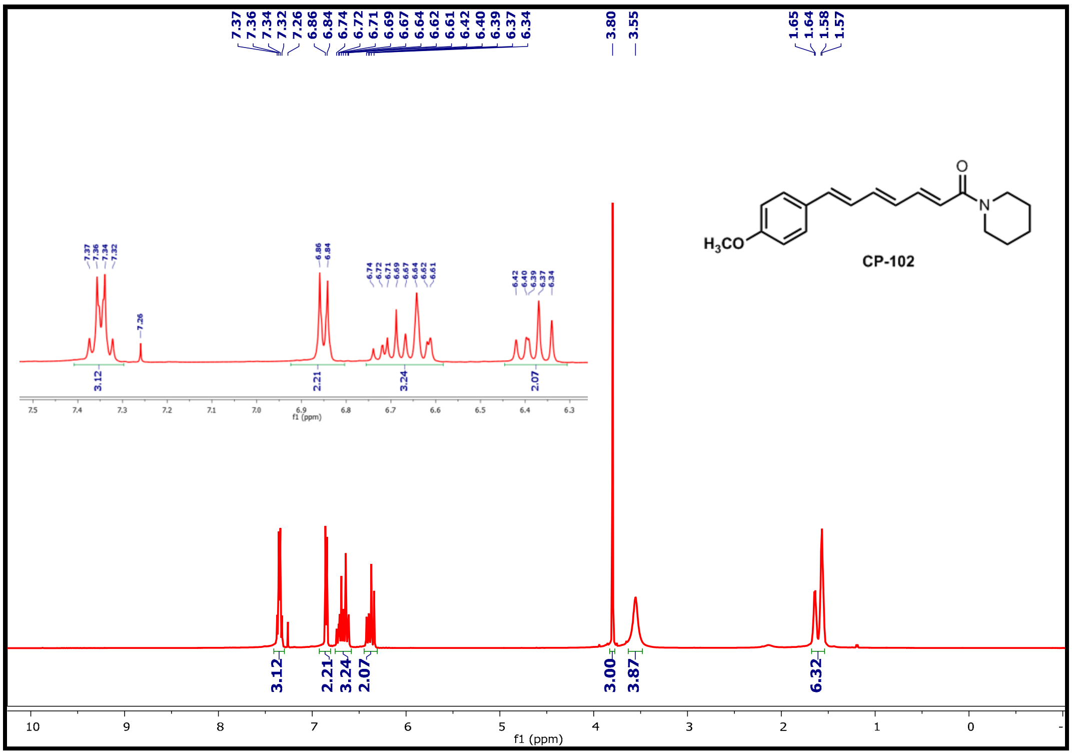

Figure 59: 500 MHz ^{1}H NMR spectrum of **CP-102** in $CDCl_3$

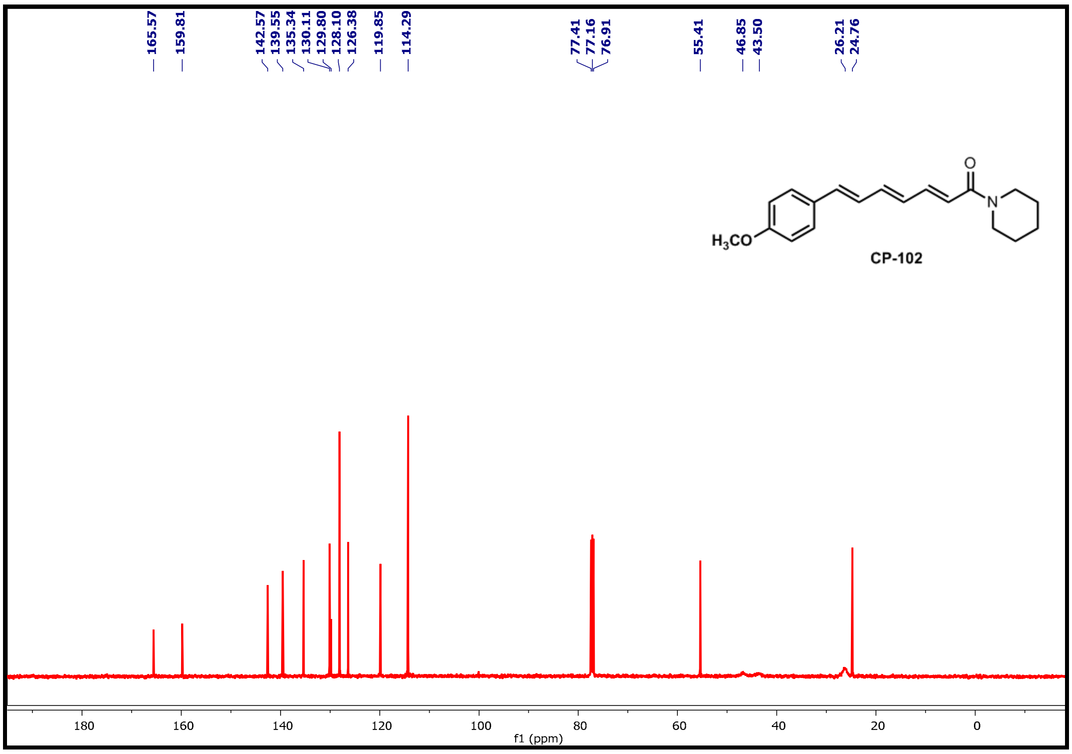

Figure 60: 125 MHz ^{13}C NMR spectrum of **CP-102** in $CDCl_3$

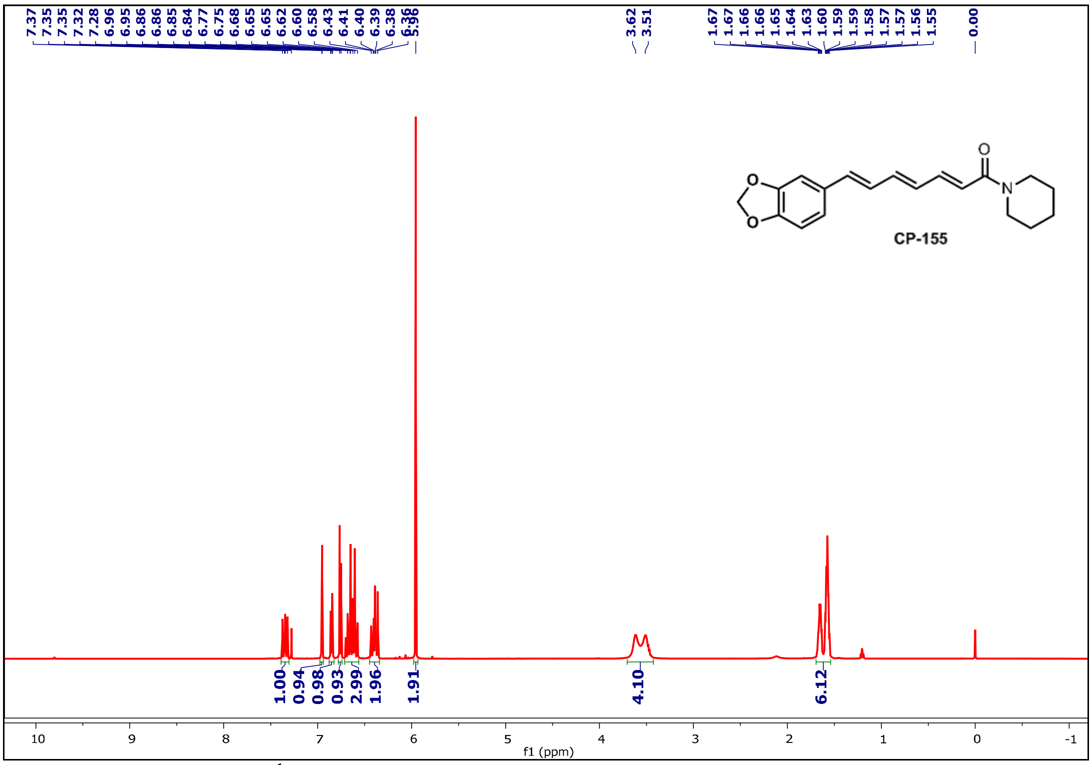

Figure 61: 500 MHz ^{1}H NMR spectrum of **CP-155** in $CDCl_3$

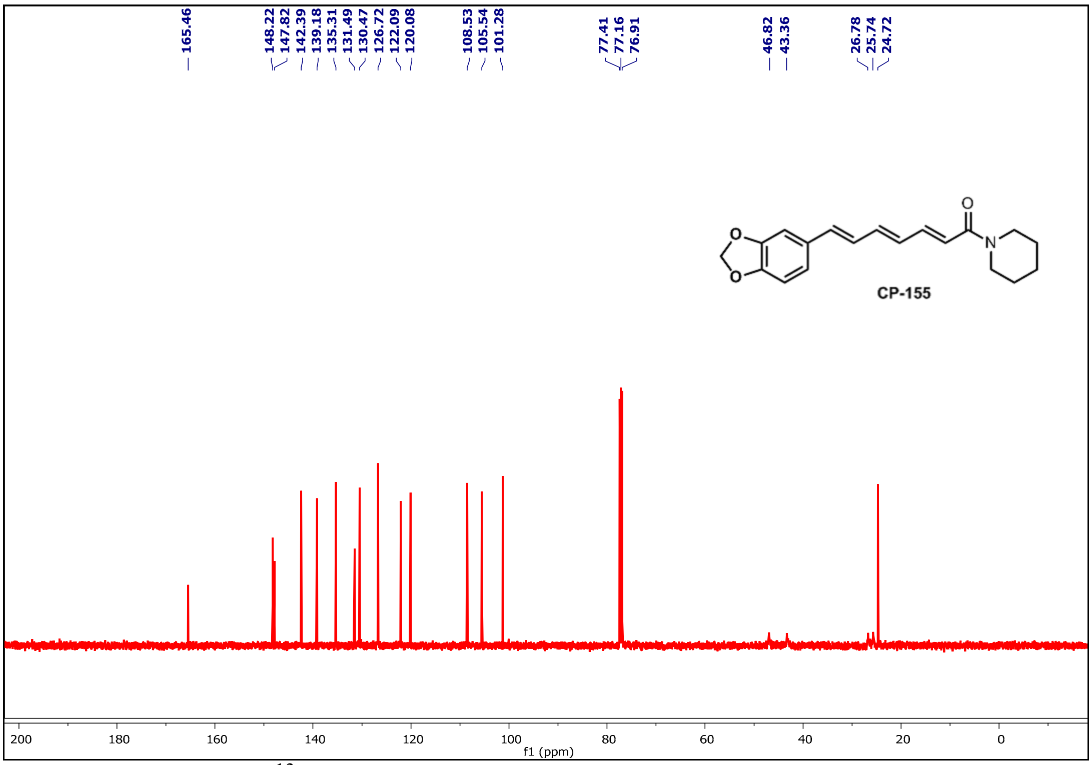

Figure 62: 125 MHz ^{13}C NMR spectrum of **CP-155** in $CDCl_3$

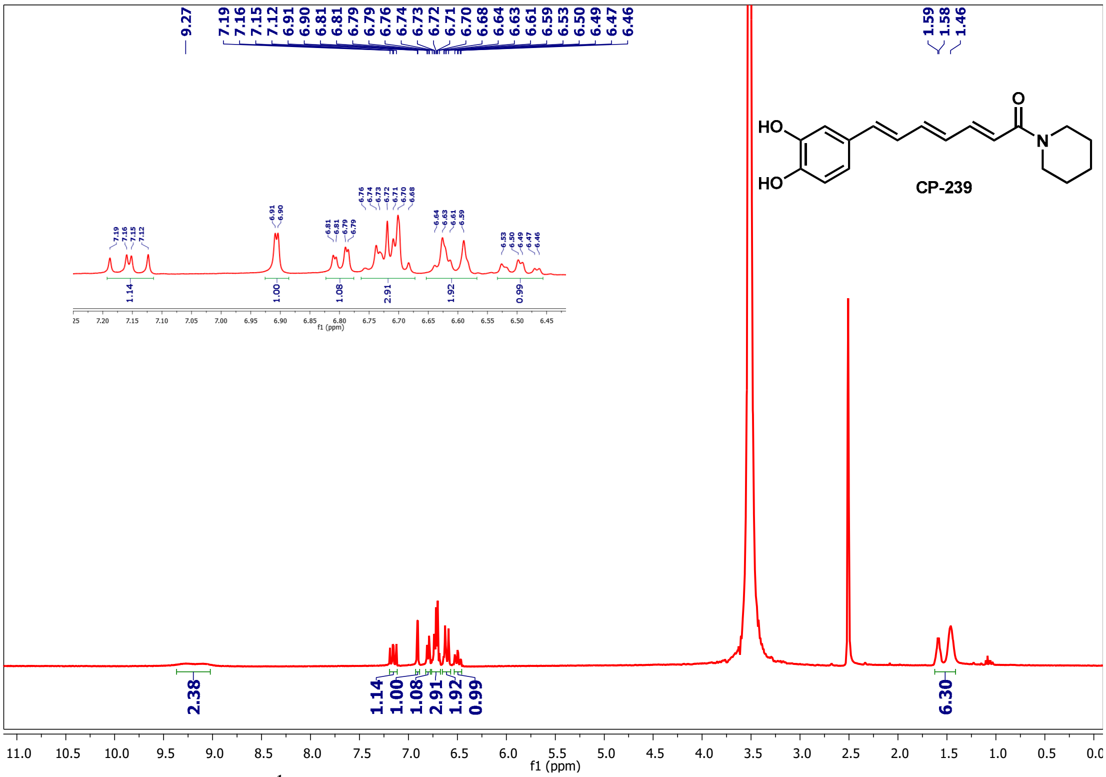

Figure 63: 400 MHz ^{1}H NMR spectrum of **CP-239** in DMSO-d_6

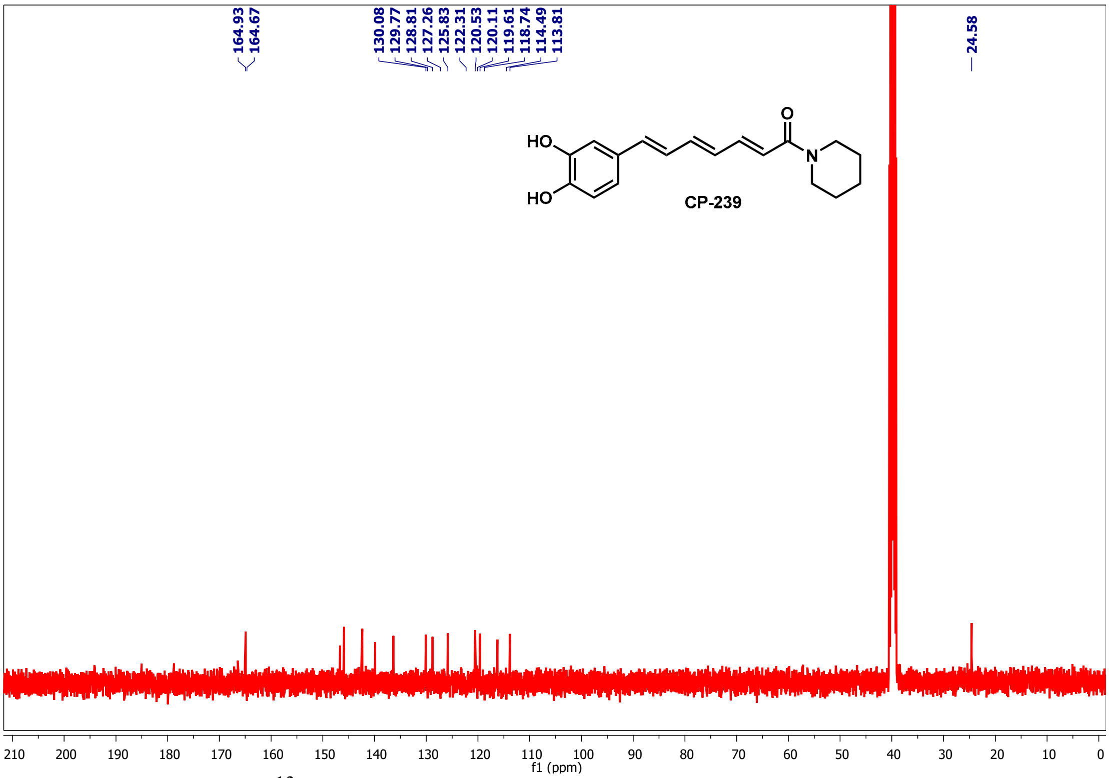

Figure 64: 100 MHz ^{13}C NMR spectrum of **CP-239** in DMSO-d_6

Quorum sensing inhibition assay

Table 1: Degree of Quorum Sensing Inhibition (QSI)- low active and non-active derivatives

Two tests were carried out by using the reporter bacteria: Agrobacterium tumefaciens (KYC55) and Chromobacterium violaceum (CV026). Bacteria were grown overnight in soft agar with the appropriate auto-inducer on a TLC plate where the different compounds were dried on (40mM in ~20µl). Their degree of QSI was measure, semi quantitative of pigment intensity (– no inhibition, + low inhibition, ++ medium inhibition, +++ strong inhibition). Experiments were repeated three times result was determine in 2 out of 3 experiments with the same result.

Entry	Monoconjugated -CP	Degree of QSI of CV026	Degree of QSI of KYC55
1.	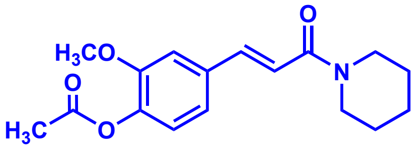 CP-291	-	+
2.	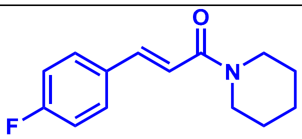 CP-273	+	-
Entry	Diconjugated-CP	Degree of QSI of CV026	Degree of QSI of KYC55
3.	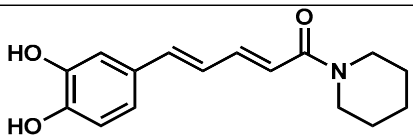 CP-209	+	+
4.	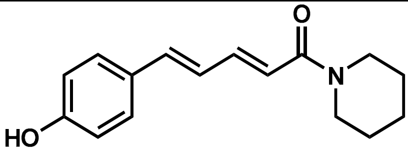 CP	+	-
5.	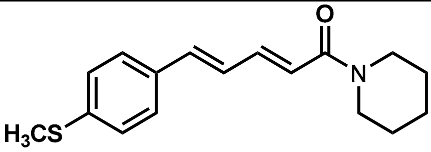 CP-147	+	-

6.	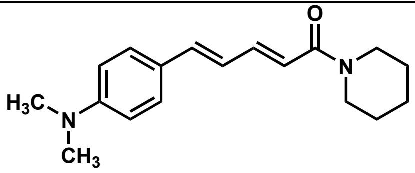 CP-32	+	-
7.	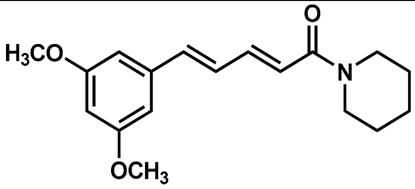 CP-193	+	-
8.	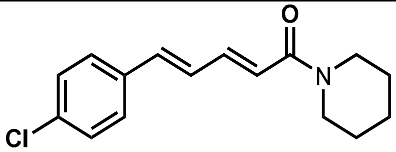 CP-123	-	+
9.	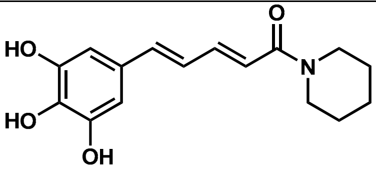 CP-262 F2	-	+
10.	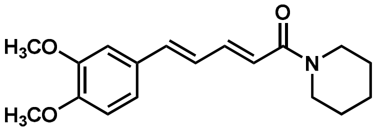 CP-10	-	-
11.	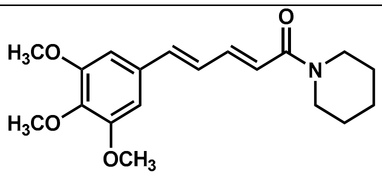 CP-27	-	-
12.	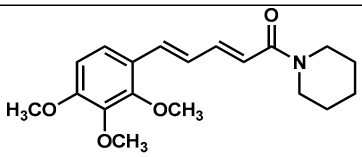 CP-50	-	-
13.	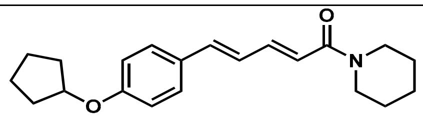 CP-184	-	-
14.	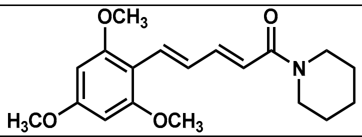	-	-

	CP-194		
15.	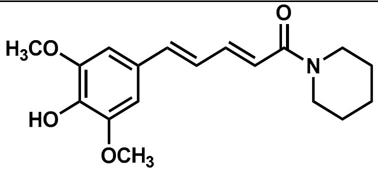 CP-262 F1	-	-
16.	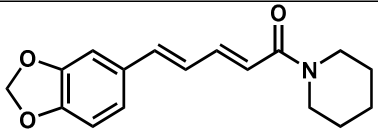 PIP	-	-
Entry	Triconjugated-CP	Degree of QSI of CV026	Degree of QSI of KYC55
17.	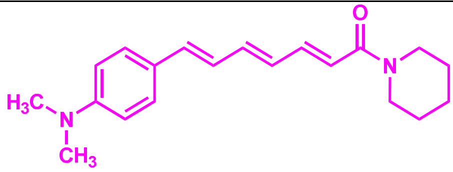 CP-146	-	-
18.	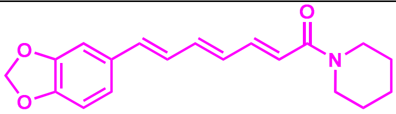 CP-155	-	-

Cell viability assay

Table 2: XTT viability test of coumaperine derivatives.

Compounds code	LC50 (uM)
CP-215	Non-toxic
CP-273	Non-toxic
CP-296	Non-toxic
CP-281F1	Non-toxic
CP-286	Non-toxic
CP-289	Non-toxic
CP-270	Non-toxic
CP-282	Non-toxic
CP-295	408.27
CP-158	285.38
CP-38	47.44
CP-154	46.78

CP-9	24.70

*L428 cells were incubated with the different compounds with QSI activity for 48 hours. Cell viability was determined by the followed by the XTT cell test. Absorbance (450nm) was determined in triplicate in at least two independent tests. The results were normalized to vehicle (DMSO) treated cells, (100% viability). LC50 concentrations are shown
